# Habitat Heterogeneity Drives Intertidal Macrobenthic Community Differentiation via Environmental Filtering, Species Turnover, and Ecological Network Reorganization

**DOI:** 10.3390/ani16162630

**Published:** 2026-08-21

**Authors:** Jiujiang Wang, Wuhan Lin, Junyan Cai, Zhiyang Tang, Qiyun Zhang, Yanping Chen, Ziming He, Wenhua Liu, Zonghang Zhang

**Affiliations:** 1 Guangdong Provincial Key Laboratory of Marine Biotechnology, Institute of Marine Sciences, Shantou University, Shantou 515063, China; 23jjwang@stu.edu.cn (J.W.); gdlinwuhan@163.com (W.L.); 24jycai@stu.edu.cn (J.C.); 24zytang@stu.edu.cn (Z.T.); 21qyzhang@stu.edu.cn (Q.Z.); 25ypchen@stu.edu.cn (Y.C.); 25zmhe1@stu.edu.cn (Z.H.); whliu@stu.edu.cn (W.L.); 2 International Joint Research Center for Marine Ecological Protection and Disaster Prevention, Shantou University, Shantou 515063, China; 3 College of Fisheries, Guangdong Ocean University, Zhanjiang 524088, China

**Keywords:** intertidal ecosystem, macrobenthos, habitat heterogeneity, alpha diversity, beta diversity, community structure

## Abstract

Intertidal habitat heterogeneity can shape macrobenthic communities, but its links to assembly processes and network stability remain uncertain. Macrobenthos were surveyed at 22 sites across muddy sediment (MA), oyster farming (OF), eastern rocky reef (EA), and southern rocky reef (SA) habitats around Nan’ao Island. Habitat type explained 25.5% of compositional variation, whereas observed richness and evenness showed no consistent among habitat differences. Mean between-habitat Bray-Curtis dissimilarity (0.730) and turnover (0.623) exceeded their within-habitat values (0.634 and 0.555), indicating that species replacement, rather than nested species loss, dominated community differentiation. Functional richness differed among habitats, whereas most other functional indices remained comparatively stable. Neutral-model fits were stronger in EA and SA (R^2^ = 0.501 and 0.475, respectively) than in MA and OF, and null-model partitioning indicated undominated processes in MA and OF but greater homogenizing dispersal in rocky habitats. SA contained the largest co-occurrence network (24 nodes, 81 edges), although robustness was highest in OF. Exploratory PLS-SEM showed positive associations between habitat heterogeneity and environmental filtering (β = 0.591, *p* = 0.004) and between turnover and potential network stability (β = 0.593, *p* = 0.022), whereas the assembly–stability association was nonsignificant (β = 0.044, *p* = 0.856). These cross-sectional associations support a conceptual pathway linking habitat-related environmental gradients, species turnover, assembly shifts, and network reorganization.

## Simple Summary

Intertidal zones are important coastal habitats where many bottom-dwelling animals live, feed and help maintain ecosystem functions. However, different intertidal habitats may support different animal communities. This study investigated macrobenthic animals from four types of intertidal habitats around Nan’ao Island, including muddy sediments, oyster farming areas and rocky shores. Habitat differences did not simply change the number of species. Instead, each habitat supported different species groups and ecological roles. Environmental conditions such as water depth, temperature, nutrients and sediment characteristics helped explain these differences. Rocky shore habitats also showed more complex community organization, suggesting that habitat structure can influence how species are arranged within communities. Overall, this study shows that protecting a mosaic of different intertidal habitats is important for maintaining regional biodiversity and the ecological functioning of coastal ecosystems.

## 1. Introduction

Intertidal zones are highly dynamic coastal ecosystems that support intensive exchanges of matter, energy, and nutrients at the land–sea interface, thereby contributing to benthic-pelagic coupling and coastal biogeochemical cycling [1,2]. Macrobenthic organisms are integral components of intertidal food webs, functioning as consumers, prey resources, and ecosystem engineers that can mediate trophic linkages and modify habitat structure [3,4]. Through feeding, burrowing, bioturbation, and sediment reworking, macrobenthos regulate organic matter decomposition, nutrient regeneration, and benthic-pelagic coupling, thereby contributing substantially to the functioning of shallow coastal ecosystems [5]. Because many macrobenthic species have limited mobility and respond sensitively to substrate alteration, organic enrichment, hypoxia, and other environmental stressors, changes in their abundance, diversity, and community composition have long been used to assess benthic habitat quality and ecological degradation [6]. Clarifying the mechanisms underlying the spatial differentiation of macrobenthic communities is therefore essential for understanding the structure, functioning, and environmental responses of intertidal ecosystems.

Habitat heterogeneity is a fundamental driver of biodiversity maintenance and spatial community differentiation. Heterogeneous habitats increase niche availability, alter the spatial distribution of resources and refuges, and modify dispersal and colonization processes, thereby creating contrasting environments for species with different ecological requirements [7,8]. According to environmental filtering theory, local conditions favor species whose ecological requirements and functional traits match the environment, while restricting the establishment and persistence of unsuitable species [9]. In intertidal ecosystems, substrate type, sediment grain size, attachment space, microtopographic complexity, hydrodynamic exposure, nutrient availability, and anthropogenic disturbance jointly constitute multidimensional environmental templates. Such contrasts are particularly pronounced around islands, where natural muddy sediments and rocky shores may coexist with aquaculture-modified habitats over relatively short spatial distances. These habitats differ in substrate stability, food availability, attachment opportunities, and disturbance regimes, thereby favoring species with different feeding strategies, mobility, burrowing behavior, attachment capacity, and stress tolerance. Studies of marine and intertidal macrobenthos have shown that environmental and anthropogenic gradients are associated with shifts in functional-trait composition, while trait-based approaches provide a useful framework for linking community structure with ecosystem functioning [10,11]. At the community level, sediment properties, natural environmental variation, and human activities can jointly shape the spatial distribution and composition of coastal macrobenthic assemblages [12,13]. Habitat-specific environmental filtering may therefore produce distinct assemblages, even when local species richness remains broadly comparable among habitats.

Despite increasing recognition of habitat-driven variation in intertidal macrobenthos, three linked gaps remain. First, compositional differences are rarely partitioned into turnover and nestedness, limiting inference about species replacement versus ordered species loss. Second, taxonomic, functional, and assembly responses are often examined separately, although similar richness can mask substantial shifts in species identity and occupy trait space [14]. Third, habitat-specific co-occurrence organization is commonly described without testing whether its stability-related properties covary with turnover or inferred assembly. Resolving these gaps is important because conservation decisions based only on local richness may overlook complementary habitats that sustain distinct taxa, functions, and responses to disturbance. Co-occurrence patterns are treated here as statistical associations, not direct biological interactions, and network size or connectivity is not assumed to imply stability [15,16,17].

An integrated framework was therefore used to connect taxonomic and functional diversity, beta-diversity partitioning, environmental gradients, assembly proxies, and co-occurrence network properties within a single intertidal landscape around Nan’ao Island, including muddy sediment area (MA), oyster farming area (OF), eastern rocky reef area (EA), and southern rocky reef area (SA). This framework was designed to determine whether habitat differentiation primarily alters species identity and occupied trait space, to distinguish species replacement from nested species loss, and to evaluate whether habitat-related environmental gradients and turnover are associated with community assembly and potential network stability. Specifically, four predictions were tested: (a) habitat type would affect community composition more strongly than local alpha diversity; (b) functional responses would be selective, rather than uniform, across indices; (c) among-habitat beta diversity would be dominated by species turnover; and (d) habitat-related environmental gradients and turnover would be associated with assembly proxies and potential network stability. Structural equation modelling was used only as an exploratory integration of these hypothesized associations, because the single-season observational design does not permit causal inference [18].

## 2. Materials and Methods

### 2.1. Study Area and Sampling Design

**Figure 1:**
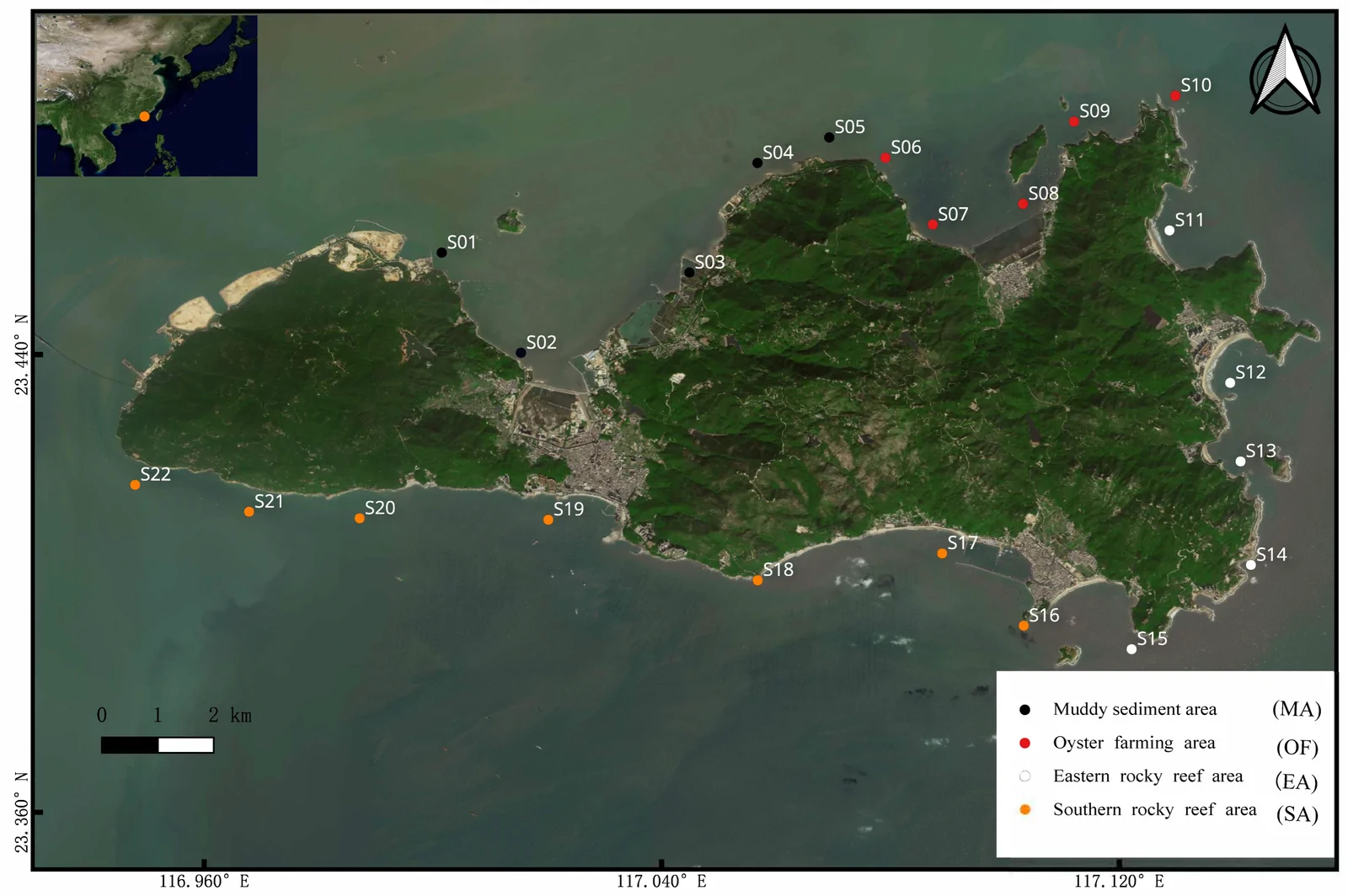
Map of the study area and sampling sites around Nan’ao Island, China.

This study was conducted around Nan’ao Island, Guangdong Province, China (116.9–117.2° E, 23.3–23.5° N), on the northern continental shelf of the South China Sea [19]. The 22 sites spanned the main island’s approximately 77 km coastline and encompassed contrasting soft-sediment, aquaculture-modified, and rocky intertidal shores (Figure 1). The region has a subtropical monsoon climate, complex tidal dynamics, and heterogeneous coastal geomorphology.

Field sampling was conducted in May 2024. Sites were selected to maximize habitat representativeness and spatial coverage subject to safe field access and were classified a priori as MA (S01–S05; *n* = 5 sites), OF (S06–S10; *n* = 5), EA (S11–S15; *n* = 5), or SA (S16–S22; *n* = 7). The design was therefore spatially extensive but temporally restricted to one survey period, and slightly unbalanced among habitat groups; both features were considered when interpreting inferential and model-based results.

At each sampling site, macrobenthic samples were collected from the high, middle and low intertidal zones to cover the vertical tidal gradient. These tidal zones represent differences in inundation duration, aerial exposure and desiccation stress. Unless otherwise stated, habitat type was used as the primary grouping factor in subsequent analyses, and the resulting dataset was analyzed using a species abundance table.

### 2.2. Field Sampling

#### 2.2.1. Collection and Processing of Macrobenthic Samples

Macrobenthic samples were collected using a transect-quadrat approach, following standard procedures for quantitative surveys of soft-sediment and rocky intertidal communities [20,21,22]. Along each transect, samples were collected from the high, middle and low intertidal zones.

For muddy, sandy and mixed muddy-sandy substrates, quadrats of 0.25 m × 0.25 m were sampled to a depth of 0.30 m. Two replicate quadrats were collected from the high intertidal zone, and three replicate quadrats were collected from both the middle and low intertidal zones. For rocky shore habitats, two 0.25 m × 0.25 m quadrats were sampled in each tidal zone. In areas with high organism density, smaller 0.10 m × 0.10 m quadrats were used for supplementary sampling.

Samples were rinsed with in situ seawater and then filtered through a sieve with a mesh size of 500 μm. Macrobenthic organisms were preliminarily sorted, separated by major taxonomic groups, fixed in 5% formalin solution, and labelled with sampling time, site, tidal zone and substrate type [22,23]. All samples were transported to the laboratory for taxonomic identification, enumeration and biomass measurement. Species identification was conducted to the lowest possible taxonomic level under a stereomicroscope using regional taxonomic monographs and identification guides for Chinese marine benthic fauna, including *Fauna Sinica* volumes for Polychaeta and Mollusca, *Atlas of Marine Mollusks in China, Checklist of Marine Biota of China Seas*, *The Classification System of Benthos from Coastal Waters of China Seas*, and *Checklist of Common Benthic Species from Coastal Waters of China Seas* [24,25,26,27,28,29,30,31]. Scientific names, taxonomic hierarchy and synonymies were further checked and updated according to the World Register of Marine Species (WoRMS), with the GBIF Backbone Taxonomy and Catalogue of Life used as supplementary references for unresolved or recently revised names [32,33,34].

#### 2.2.2. In Situ Water Properties and Depth

To characterize habitat heterogeneity, environmental variables were measured or compiled across multiple dimensions, including water physicochemical properties, nutrient conditions, sediment characteristics, coastal structural complexity, hydrodynamic conditions and anthropogenic/spatial context.

In situ water properties, including temperature (T, °C), salinity (S), pH and dissolved oxygen (DO, mg/L) were measured using a multiparameter water-quality analyzer (HI98194, Hanna Instruments, Woonsocket, RI, USA). Water depth was measured ten times at each site using a portable depth sounder (SM-5A, Laylin/Speedtech, Unionville, VA, USA), and the mean value was recorded as depth. The coefficient of variation of the ten measurements was calculated as depthCV. Surface seawater temperature and salinity were measured near each transect during low tide.

#### 2.2.3. Water Sampling and Laboratory Chemistry

Water samples were collected and transported to the laboratory under refrigerated conditions for chemical analyses. Chlorophyll a (Chl-a) was determined spectrophotometrically following the Specifications for Oceanographic Survey—Part 6: Marine Biological Survey [35]. Nutrient variables, including nitrite nitrogen (NO_2_-N), nitrate nitrogen (NO_3_-N), ammonium nitrogen (NH_4_-N), total nitrogen (TN), phosphate phosphorus (PO_4_-P), silicate silicon (SiO_3_-Si) and suspended solids (SS), were analyzed according to the *Specifications for Oceanographic Survey—Part 4: Survey of Chemical Parameters in Sea Water* [36].

#### 2.2.4. Sediment Collection and Grain-Size Analysis

Sediment samples were collected for grain-size analysis where field conditions allowed. Intertidal sediment collection was unsuccessful at several sites, as some rocky-reef sites did not contain enough unconsolidated sediment to perform standardized grain-size sampling. To ensure consistency in substrate background among all sites, nearby subtidal sediment samples were used as site-level proxies. These sediment data should thus be interpreted as indicators of regional substrate conditions, instead of direct measurements for each intertidal quadrat. Grain-size analysis was conducted using a Beckman Coulter LS 13320 laser diffraction particle-size analyzer (Beckman Coulter Inc., Brea, CA, USA), following general methodological recommendations for sediment particle-size characterization [37]. Derived sediment indices included mean, median, mode, standard deviation, coefficient of variation and percentile particle sizes (D10, D25, D50, D75 and D90).

#### 2.2.5. Coastal Structural Complexity

Coastal structural complexity was quantified using images taken from the upper, middle and lower intertidal belts with a fixed photo box (Figure S3). The mean fractal dimension (D) of the three tidal levels was calculated for each site as an index of habitat complexity. Fractal dimension has been widely used as a quantitative metric of spatial complexity and habitat heterogeneity [38].

#### 2.2.6. Anthropogenic, Spatial, and Hydrodynamic Proxies

Anthropogenic and spatial proxy variables were compiled at the site level. The distance from each sampling site to the Hanjiang River estuary was calculated using a Geographic Information System (GIS). Distance-weighted GDP and population indices were calculated based on adjacent town centers using GDP and population data from the *2024 Statistical Bulletin of National Economic and Social Development of Nan’ao County*. Hydrodynamic variables, including eastward current velocity, northward current velocity and sea-surface height, were extracted retrospectively from the MyOcean Viewer based on site coordinates and the corresponding sampling period using the nearest grid cell [39].

### 2.3. Statistical Analyses

All analyses were conducted in R 4.3.1. The species abundance matrix was the basis for taxonomic and functional diversity, beta diversity, assembly, and co-occurrence analyses. Distributional assumptions were checked before parametric tests; otherwise, permutation or rank-based procedures were used. Pairwise *p*-values were adjusted by the Benjamini–Hochberg method. Because only 22 sites were available and habitat groups were slightly unbalanced, effect sizes, permutation results, bootstrap confidence intervals, and cross-validation were emphasized over isolated nominal significance. Complex models were treated as exploratory, and were not interpreted as causal evidence.

#### 2.3.1. Taxonomic Diversity, Functional Diversity and Beta Diversity

Taxonomic diversity was calculated from the species abundance table to assess within-sample diversity of macrobenthic communities. Six indices were calculated: observed richness (Sobs/Observed), Chao1, ACE, Shannon diversity, Simpson diversity (1-D) and Pielou’s evenness. Sobs represents the observed number of species; Chao1 and ACE estimate richness with emphasis on rare species; Shannon and Simpson indices reflect both richness and dominance structure; and Pielou’s evenness measures the evenness of species abundances. Diversity analyses were performed using the original species abundance matrix, without rare-species filtering, to avoid biasing richness estimation. Group differences among habitats were tested using one-way ANOVA when assumptions of normality and homogeneity of variance were satisfied; otherwise, Kruskal-Wallis tests were used. Pairwise comparisons were performed using Wilcoxon rank-sum tests.

Functional traits for all macrobenthic species were compiled from publicly available trait databases and taxon-specific literature. Online databases included the WoRMS Marine Species Traits [40], BIOTIC (Biological Traits Information Catalogue) [41], and Polytraits, a database for biological traits of marine polychaetes [42,43]. The selected traits spanned morphological features, habitat utilization, mobility, trophic ecology, ecosystem functioning, and environmental tolerance, comprising body size, living position, substrate preference, locomotion, feeding mode, trophic group, bioturbation potential, larval development, dispersal potential, salinity tolerance, and desiccation tolerance. Species-level trait data were adopted wherever possible. Where species-specific information was absent, genus- or family-level traits were cautiously assigned, based on the most closely related taxa and supporting literature. Of the 24 taxa in the trait-matching dataset, 18 (75.0%) obtained direct species-level matches, while the remaining six taxa (25.0%) relied on taxonomic proxies (Table S13). Such proxy assignments can introduce uncertainties into trait-derived estimates, which were therefore taken into account when interpreting functional-diversity patterns.

A species-by-trait matrix was constructed, and Gower distance was calculated to accommodate mixed trait types. Functional richness (FRic), functional evenness (FEve) and functional divergence (FDiv) were calculated following Villéger et al. (2008) [44]. Functional dispersion (FDis) was calculated following Laliberté and Legendre (2010) [45], and Rao’s quadratic entropy (RaoQ) was calculated following Botta-Dukát (2005) [46]. Functional redundancy (FRed) was calculated to quantify overlap in functional roles among coexisting species, following Mouillot et al. (2014) and Ricotta et al. (2016) [47,48]. Differences in functional diversity among habitats were evaluated using Kruskal-Wallis tests and pairwise Wilcoxon rank-sum tests.

Beta diversity was assessed using Bray-Curtis dissimilarity based on the species abundance matrix. Principal coordinates analysis (PCoA) and non-metric multidimensional scaling (NMDS) were used to visualize community compositional differences among habitats. Group-level differences were tested using PERMANOVA [49] and ANOSIM. Beta dispersion was evaluated to determine whether differences among habitats were associated with unequal within-group dispersion [50]. In addition, total beta diversity was partitioned into turnover and nestedness components using the presence–absence species matrix under the Baselga framework [51].

The R packages stats (v4.3.1), vegan (v2.6-6), FD (v1.0-12) and betapart (v1.6) were used for these analyses. Specifically, stats was used for ANOVA, Kruskal–Wallis tests and pairwise Wilcoxon rank-sum tests; vegan was used for diversity estimation, Bray-Curtis dissimilarity, NMDS, PERMANOVA, ANOSIM and beta-dispersion; FD was used for calculating FRic, FEve, FDiv, FDis and RaoQ; and betapart was used for beta-diversity partitioning.

#### 2.3.2. Screening of Environmental Drivers

To identify key environmental drivers of habitat-level community differentiation, environmental and community datasets were matched at the site level. Environmental variables were first screened using univariate permutation-based tests, and variables with permutation *p* < 0.10 were retained as candidate predictors. To reduce multicollinearity, variance inflation factor (VIF) analysis was then applied iteratively until all retained variables had VIF < 10.

Based on the non-redundant environmental dataset, principal component analysis (PCA) was used to describe environmental separation among habitat groups. Forward stepwise multinomial logistic regression was performed to identify core predictors of habitat differentiation, and model improvement was evaluated using AIC, likelihood-ratio tests and McFadden’s R^2^. A random forest classifier was also constructed using the same variable set to rank nonlinear variable importance [52]. Leave-one-out cross-validation (LOOCV) was used to compare model performance using accuracy, balanced accuracy, macro-F1 and Cohen’s K. 

To quantify relationships between environmental gradients and species-level community composition, redundancy analysis (RDA) and distance-based redundancy analysis (dbRDA) were conducted using the selected environmental variables. dbRDA was performed following the framework of Legendre and Anderson (1999) [53].

These analyses were conducted using vegan (v2.6-6) for permutation tests and constrained ordination, car (v3.1-2) for VIF analysis, nnet (v7.3-19) for multinomial logistic regression, MASS (v7.3-60) for stepwise model selection, lmtest (v0.9-40) for likelihood-ratio tests, pscl (v1.5.5) for McFadden’s R^2^, randomForest (v4.7-1.2) for random forest classification, caret (v6.0-94) for LOOCV, and yardstick (v1.3.0) for model performance evaluation. All base statistical operations relied on stats (v4.3.1). 

#### 2.3.3. Community Assembly Analysis

To infer community assembly processes of macrobenthic assemblages across habitats, neutral-model and abundance-based null-model approaches were applied to the species abundance table. The Sloan neutral community model (NCM) was fitted to estimate the migration parameter (m) and model fit (R^2^), following Sloan et al. (2006) [54]. Species were classified as occurring within, above or below the 95% neutral prediction interval, and the proportions of these categories were calculated for each habitat.

In parallel, abundance-based null-model analyses were used to infer the relative contributions of deterministic and stochastic processes. Because this study was based on species abundance data, rather than ASV/OTU sequences or a phylogenetic tree, βNTI-related results were interpreted as approximate null-model evidence of assembly processes, rather than strict phylogenetic inference. Null-model randomizations were repeated 1000 times for βNTI-related metrics and 500 times for Raup-Crick metrics. Assembly processes were classified according to βNTI and Raup-Crick thresholds, following the general framework of Stegen et al. (2013, 2015) [55,56].

Geographic- and environmental-distance matrices were also calculated to evaluate distance–decay relationships. Geographic distance was calculated from site coordinates, and environmental distance was calculated from standardized environmental variables. Linear regression was used to assess relationships between community similarity and geographic or environmental distance. Levin’s niche breadth and the proportion of shared species based on Jaccard similarity were also calculated, to characterize habitat-level differences in niche occupation and species sharing.

These analyses were conducted using pctax (v0.1.10) for the Sloan neutral community model, iCAMP (v1.5.2) for null-model analyses, vegan (v2.6-6) for Bray–Curtis and environmental-distance calculations, geosphere (v1.5-18) for geographic-distance calculation and stats (v4.3.1) for linear regression.

#### 2.3.4. Species Co-Occurrence Network Analysis and Stability Metrics

Species co-occurrence networks were constructed separately for each habitat group using species abundance data. Prior to network construction, low-abundance species were filtered within each habitat group to reduce noise and improve the reliability of correlation estimates. Species with total abundance greater than 50 within each habitat group were retained. Pearson correlation coefficients were then calculated for all species pairs. *p*-values were adjusted using the Benjamini–Hochberg false discovery rate correction (Benjamini and Hochberg, 1995) [57]. Only species pairs with |r| > 0.8 and BH-adjusted *p* < 0.01 were retained as network edges.

Undirected species co-occurrence networks were constructed for each habitat group. Nodes represented macrobenthic species, and edges represented significant positive or negative abundance correlations between species. Major topological properties were calculated, including number of nodes, number of edges, average degree, network density, average path length, clustering coefficient, diameter and modularity. These metrics were used to characterize network size, connectivity, complexity and compartmentalization.

To evaluate stability-related network properties, robustness, vulnerability, average variation degree (AVD) and cohesion were calculated. Robustness was estimated based on the abundance-weighted mean interaction strength (wMISi) and the proportion of nodes remaining after simulated disturbance, following Li et al. (2023) [58]. Vulnerability was calculated from changes in global network efficiency after node removal. Positive and negative cohesion values were used to characterize the overall balance of positive and negative species associations, following Xun et al. (2021) [59]. Because correlation-based networks do not directly represent true biological interactions, these network metrics were interpreted as indicators of potential species association structure and stability-related properties, rather than direct evidence of trophic, competitive or facilitative interactions.

Pearson correlation analysis and BH correction were performed using the stats (v4.3.1) package, network construction and topological analysis were conducted using igraph (v1.4.3), and robustness, vulnerability, AVD and cohesion were calculated using custom R scripts written in base R (v4.3.1).

#### 2.3.5. Partial Least Squares Structural Equation Modelling

Partial least squares structural equation modelling (PLS-SEM) was used to integrate habitat heterogeneity, environmental filtering, beta-diversity turnover, community assembly and network stability into an integrated conceptual framework of hypothesized associations. PLS-SEM was selected because it is suitable for exploratory ecological models involving multiple interrelated variables and relatively small sample sizes [18]. All variables were centered and standardized, prior to modelling.

Based on the study hypotheses and final analytical framework, two PLS-SEM models were constructed. The first was a core assembly model linking habitat heterogeneity, environmental filtering, beta-diversity turnover and community assembly. The second was a stability extension model that further incorporated network stability. Habitat heterogeneity was represented by habitat grouping and related habitat variables. Environmental filtering was represented by the retained key environmental predictors identified through environmental screening and constrained ordination. Beta-diversity turnover was represented by the turnover component derived from beta-diversity partitioning. Community assembly was represented by neutral-model- and null-model-derived assembly metrics. Network stability was represented by stability-related network indices, including robustness, vulnerability, AVD and cohesion.

Path coefficients were estimated from the final models, and their significance was evaluated using non-parametric bootstrap resampling with 2000 iterations. Paths whose 95% bootstrap confidence intervals did not include zero were considered statistically supported. Model performance was evaluated using the coefficient of determination (R^2^) for endogenous variables and goodness-of-fit (GOF). PLS-SEM analyses were conducted in R using plspm (v0.4.9), lavaan (v0.6-16) and psych (v2.3.6) packages under R v4.3.1.

## 3. Results

### 3.1. Community Structure of Macrobenthic Assemblages

**Figure 2:**
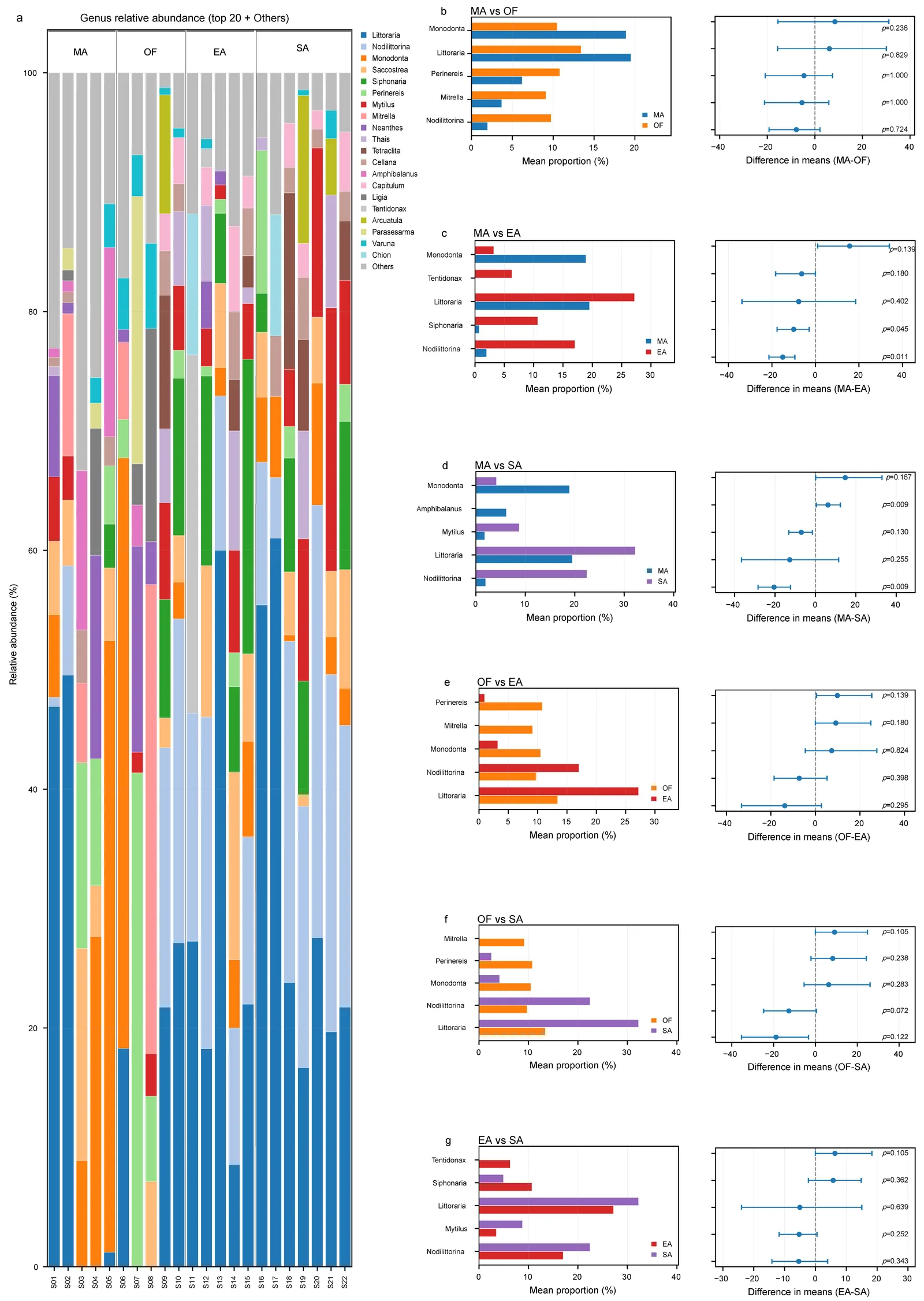
Habitat-specific species composition and differential taxa of intertidal macrobenthos. (**a**) Relative abundance of the top 20 macrobenthic taxa based on the species abundance table across four intertidal habitats: muddy sediment area (MA), oyster farming area (OF), eastern rocky reef area (EA), and southern rocky reef area (SA). Taxa outside the top 20 were grouped as “Others”. (**b**–**g**) Mean relative proportions and differences in mean abundance of the main differential taxa in pairwise habitat comparisons. Points and horizontal bars indicate mean differences and confidence intervals, respectively.

Overall, macrobenthic community composition differed markedly among the four intertidal habitats, with clear habitat-specific variation in dominant-taxon profiles (Figure 2a and Figures S1–S3; Note S1). MA supported the highest species richness and the greatest number of unique species, whereas SA had the highest total and mean site densities, but the lowest overall richness (Figure S4; Table S1). Mollusca, particularly Gastropoda, dominated the assemblages, with Littoraria, Nodilittorina, Monodonta, Siphonaria, and Mytilus as the principal genera and *Littoraria melanostoma* and *Nodilittorina pyramidalis* as the dominant species (Tables S2 and S3). Pairwise comparisons showed that the taxa contributing to community differences varied among habitat contrasts, indicating compositional shifts involving multiple taxa, rather than a single dominant species (Figure 2b–g). Genus-level screening identified nominal differences in Amphibalanus, Nodilittorina, and Varuna, but none remained significant after BH correction (Figure S5; Table S4). Selected comparisons further showed lower relative abundances of Nodilittorina and Siphonaria in MA than in EA (Table S5).

### 3.2. Patterns of Macrobenthic Taxonomic and Functional Diversity

**Figure 3:**
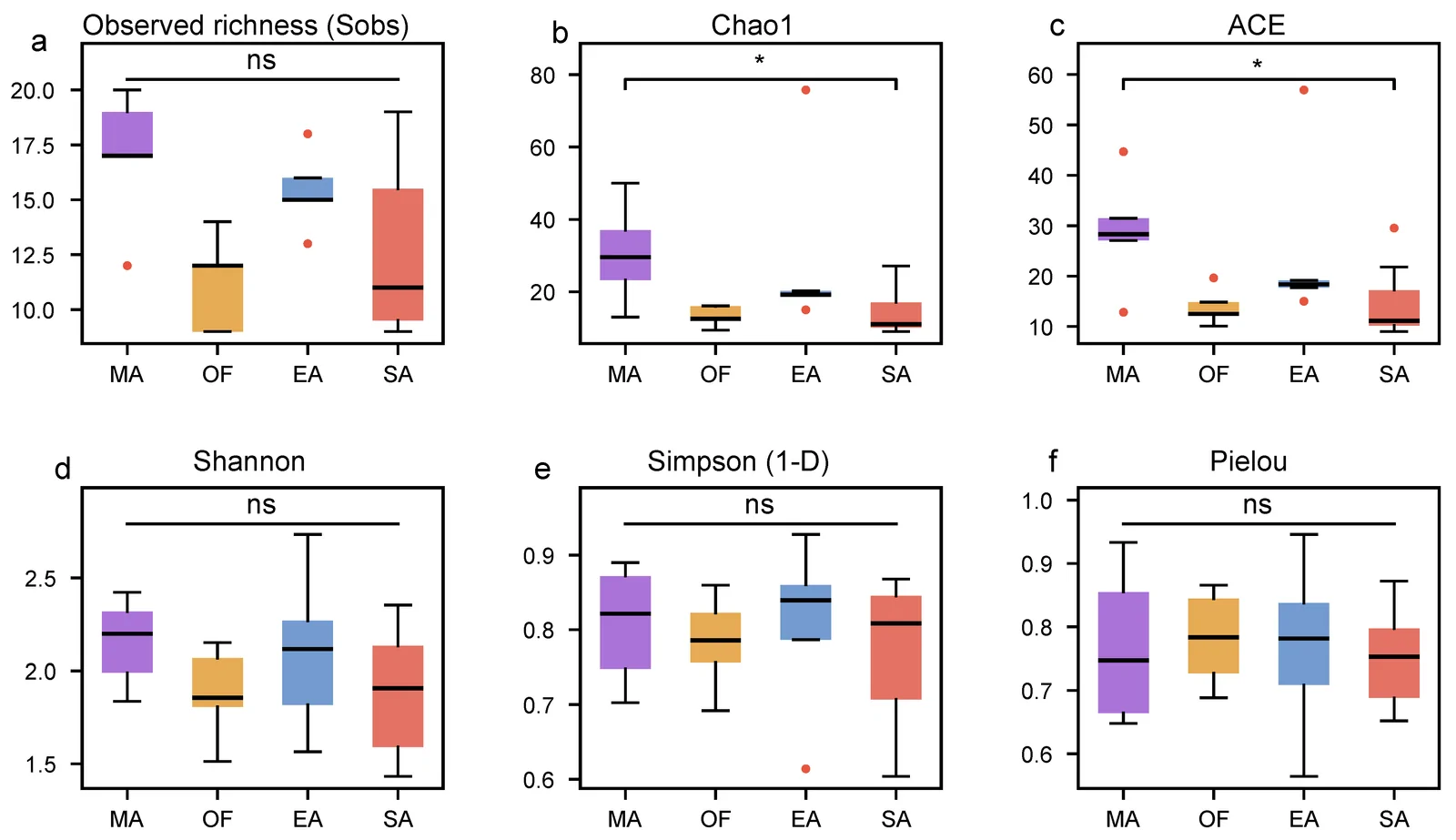
Taxonomic diversity patterns of macrobenthic communities across intertidal habitats based on species abundance data. (**a**) Observed richness (Sobs), (**b**) Chao1 richness estimator, (**c**) ACE richness estimator, (**d**) Shannon diversity, (**e**) Simpson diversity (1-D), and (**f**) Pielou’s evenness across the four habitat types: muddy sediment area (MA), oyster farming area (OF), eastern rocky reef area (EA), and southern rocky reef area (SA). Boxes represent the median and interquartile range, whiskers indicate data spread, and points indicate individual samples. Horizontal brackets denote overall among-habitat comparisons based on Kruskal–Wallis tests. Asterisks indicate significant differences among habitats (*p* < 0.05), whereas ns indicates no significant difference.

**Figure 4:**
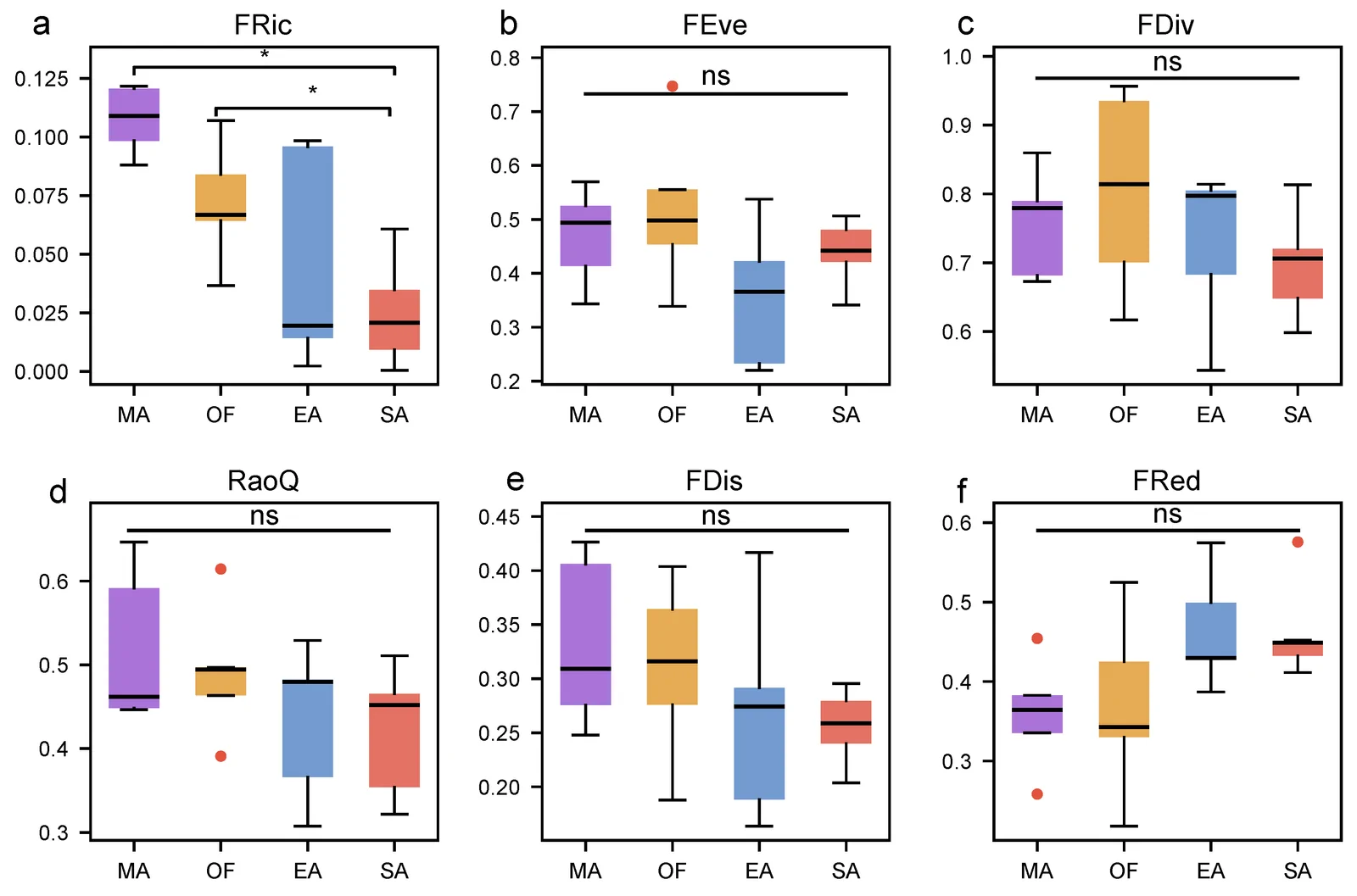
Functional diversity responses of macrobenthic communities to intertidal habitat heterogeneity based on species-level trait and abundance data. (**a**) Functional richness (FRic), (**b**) functional evenness (FEve), (**c**) functional divergence (FDiv), (**d**) Rao’s quadratic entropy (RaoQ), (**e**) functional dispersion (FDis), and (**f**) functional redundancy (FRed) across muddy sediment area (MA), oyster farming area (OF), eastern rocky reef area (EA), and southern rocky reef area (SA). Boxes represent the median and interquartile range, whiskers indicate data spread, and points indicate individual samples. Horizontal brackets denote overall among-habitat comparisons based on Kruskal–Wallis tests. Asterisks indicate significant differences among habitats (*p* < 0.05), whereas ns indicates no significant difference.

Overall, taxonomic and functional diversity showed different habitat-associated responses (Figure 3 and Figure 4; Note S2). Observed richness (Sobs), Shannon diversity, Simpson diversity, and Pielou’s evenness did not differ significantly among habitats, whereas Chao1 and ACE showed significant overall differences, with MA generally exhibiting higher estimated richness (Figure 3a–f; Tables S6 and S7). However, no pairwise alpha-diversity comparison remained significant after BH correction, and correlations between alpha-diversity indices and environmental variables were also nonsignificant after correction (Tables S8 and S9). Functional diversity showed a more selective response: FRic differed significantly among habitats, decreasing from MA and OF toward the rocky reef habitats, particularly SA, and remained significantly higher in MA and OF than in SA after BH correction (Figure 4a; Tables S10 and S11). In contrast, FEve, FDiv, RaoQ, FDis and FRed showed no significant habitat differences (Figure 4b–f; Tables S12 and S13).

### 3.3. Beta-Diversity Differentiation Among Intertidal Habitats

**Figure 5:**
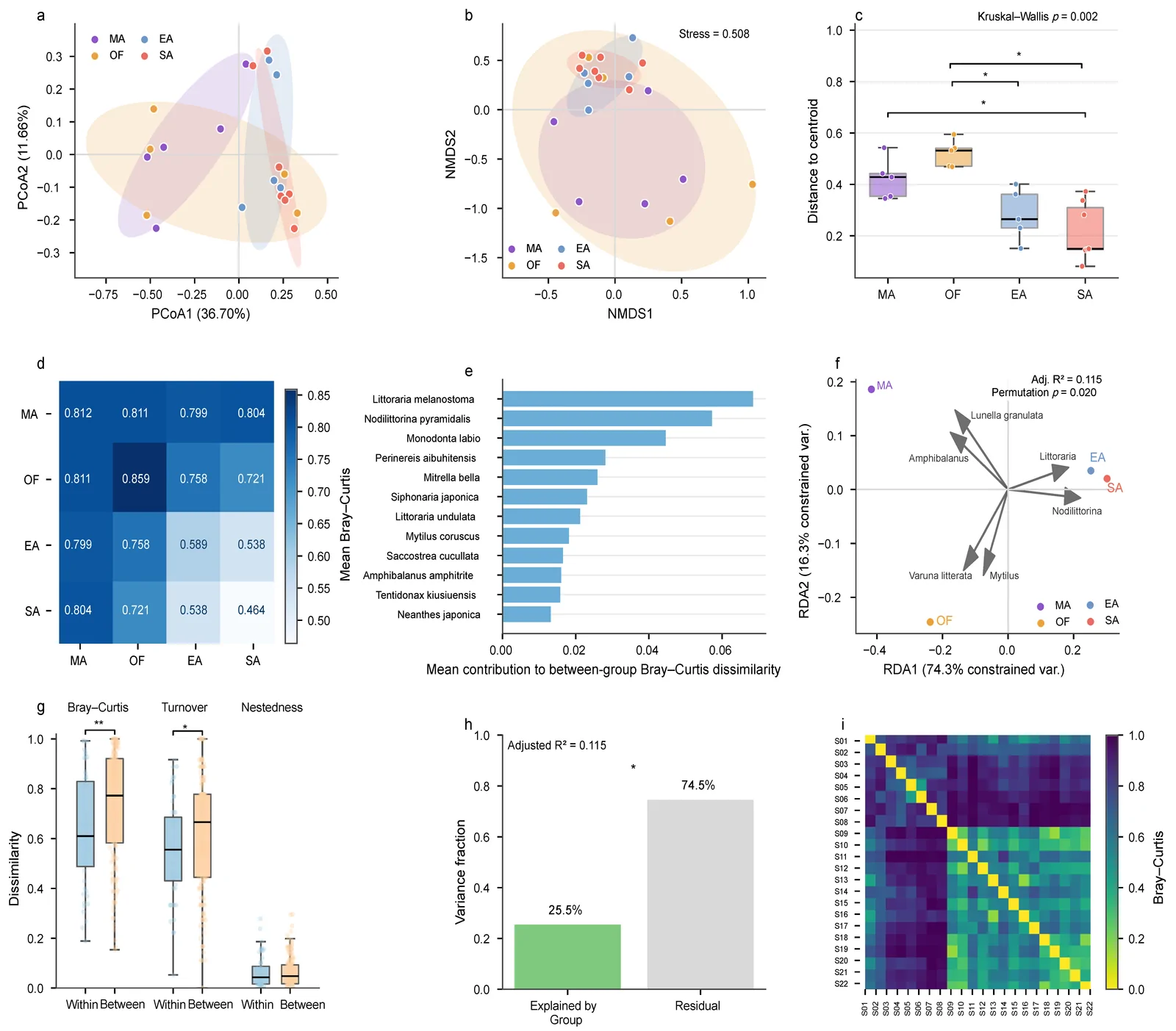
Habitat-driven beta-diversity differentiation of intertidal macrobenthic communities based on species abundance data. The four habitat types were muddy sediment area (MA), oyster farming area (OF), eastern rocky reef area (EA), and southern rocky reef area (SA). (**a**) Principal coordinate analysis (PCoA) based on Bray-Curtis dissimilarity. (**b**) Non-metric multidimensional scaling (NMDS) ordination based on Bray-Curtis dissimilarity. (**c**) Beta-dispersion analysis showing the distance of each sample to the group centroid. (**d**) Mean Bray-Curtis dissimilarity matrix among habitat types. (**e**) Major species contributing to between-habitat Bray-Curtis dissimilarity. (**f**) Redundancy analysis (RDA) showing community compositional variation constrained by habitat type. (**g**) Comparisons of within- and between-habitat total beta diversity, turnover, and nestedness components. (**h**) Variation in community composition explained by habitat type based on PERMANOVA. (**i**) Sample-wise Bray-Curtis dissimilarity heatmap. Shaded ellipses in panels (**a**,**b**) represent 95% confidence regions for the habitat groups. Horizontal lines indicate pairwise comparisons; * indicates *p* < 0.05 and ** indicates *p* < 0.01.

Overall, beta-diversity analyses revealed pronounced habitat-associated differentiation in macrobenthic community composition (Figure 5; Note S3). Bray-Curtis PCoA showed habitat-related separation, broadly supported by NMDS, although the latter had a high stress value and should therefore be interpreted cautiously (Figure 5a,b and Figure S8). Pairwise PERMANOVA identified a significant difference only between MA and SA after BH correction, while UPGMA showed partial, rather than complete, habitat-level clustering (Figures S6 and S7). Beta dispersion also differed significantly among habitats, with OF showing the greatest and SA the lowest within-habitat dispersion; corresponding site-level ordination scores were consistent with the PCoA and NMDS patterns (Figure 5c; Tables S14–S17). The habitat-level Bray Curtis dissimilarity matrix showed high compositional dissimilarity among habitats, with dominant and habitat-associated taxa contributing most to these differences (Figure 5d,e). Habitat-constrained RDA showed corresponding compositional structuring (Figure 5f), and PERMANOVA indicated that habitat grouping explained 25.5% of total community variation (Figure 5h; Table S20). Environmental associations with beta-diversity ordination were strongest for depth, total nitrogen, nitrate, temperature, silicate, and sediment grain-size variables (Figure S10). Beta-diversity partitioning further showed that between-habitat Bray-Curtis dissimilarity and turnover (0.730 and 0.623, respectively) exceeded within-habitat values (0.634 and 0.555), whereas nestedness contributed comparatively little, indicating that community differentiation was dominated by species replacement, rather than nested species loss or gain (Figure 5g and Figure S9; Table S18). The sample-wise Bray-Curtis heatmap further showed clear dissimilarity structure among samples from different habitats (Figure 5i).

### 3.4. Environmental Filtering and Key Drivers of Community Variation

**Figure 6:**
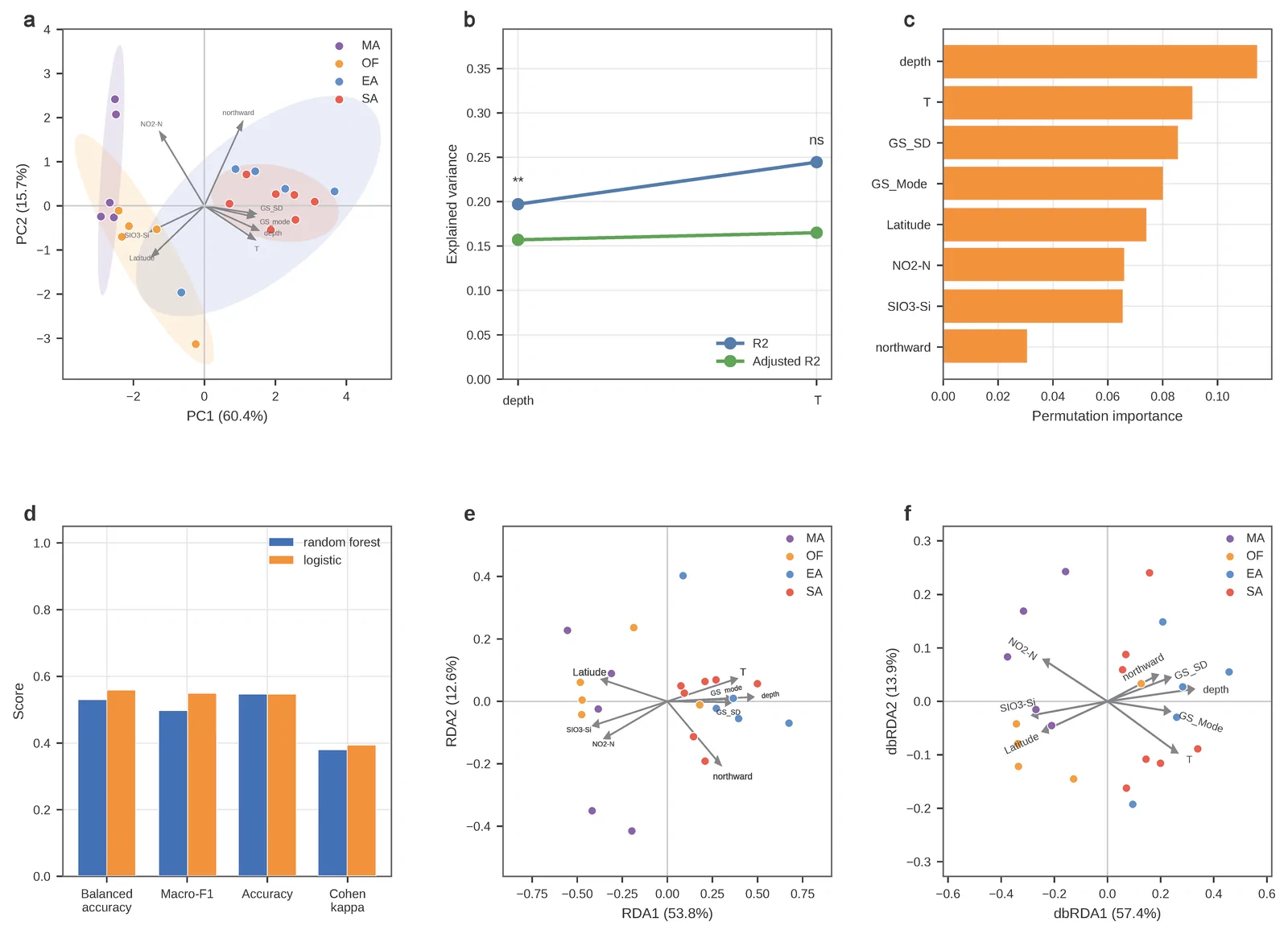
Environmental filtering and key environmental drivers of intertidal macrobenthic community variation based on species abundance data. The four habitat types were muddy sediment area (MA), oyster farming area (OF), eastern rocky reef area (EA), and southern rocky reef area (SA). (**a**) Principal component analysis (PCA) of the retained environmental variables, showing environmental differentiation among habitat types. (**b**) Forward stepwise selection of environmental predictors and changes in model explanatory power. (**c**) Random forest permutation importance of environmental variables associated with community variation. (**d**) Leave-one-out cross-validation (LOOCV) performance of the environmental classification models. (**e**) Redundancy analysis (RDA) showing community variation constrained by environmental gradients. (**f**) Distance-based redundancy analysis (dbRDA) based on Bray-Curtis dissimilarity showing community–environment relationships. In the ordination plots, points represent individual samples, colors indicate habitat types, and arrows indicate the direction and relative strength of environmental gradients. T represents water temperature, and depth represents water depth. The ns indicates non-significance, and the ** indicates *p* < 0.01. Other environmental variable abbreviations are defined in Section 2.

Overall, environmental conditions differed among habitats, and were associated with macrobenthic community variation (Figure 6; Note S4). Univariate screening identified depth, total nitrogen, silicate, temperature, nitrite, nitrate, height, and latitude as significant predictors, with depth explaining the largest individual fraction of variation (Figure S11; Table S19). Correlation analysis showed substantial covariation among sediment grain-size and nutrient variables (Figure S12); after collinearity control, eight non-redundant predictors with VIF < 10 were retained (Figures S11 and S13; Table S21), and PCA revealed environmental differentiation among MA, OF, EA, and SA (Figure 6a). Forward selection retained depth as the only significant predictor, whereas the additional contribution of temperature was nonsignificant (Figure 6b and Figure S14; Table S22). Consistently, random forest ranked depth as the most important variable, followed by temperature and sediment grain-size parameters (Figure 6c and Figure S15a; Table S23). LOOCV indicated moderate classification performance, with balanced logistic regression slightly outperforming balanced random forest in balanced accuracy and Macro-F1 (Figure 6d and Figure S15b; Table S24). RDA and dbRDA explained measurable fractions of community variation, but were nonsignificant in permutation tests, whereas habitat grouping remained significant in PERMANOVA (Figure 6e,f and Figure S11; Table S20). Axis-specific correlations further associated depth, temperature, nutrients, and sediment properties with Bray–Curtis PCoA structure (Figure S16).

### 3.5. Ecological Assembly Processes Across Habitats

**Figure 7:**
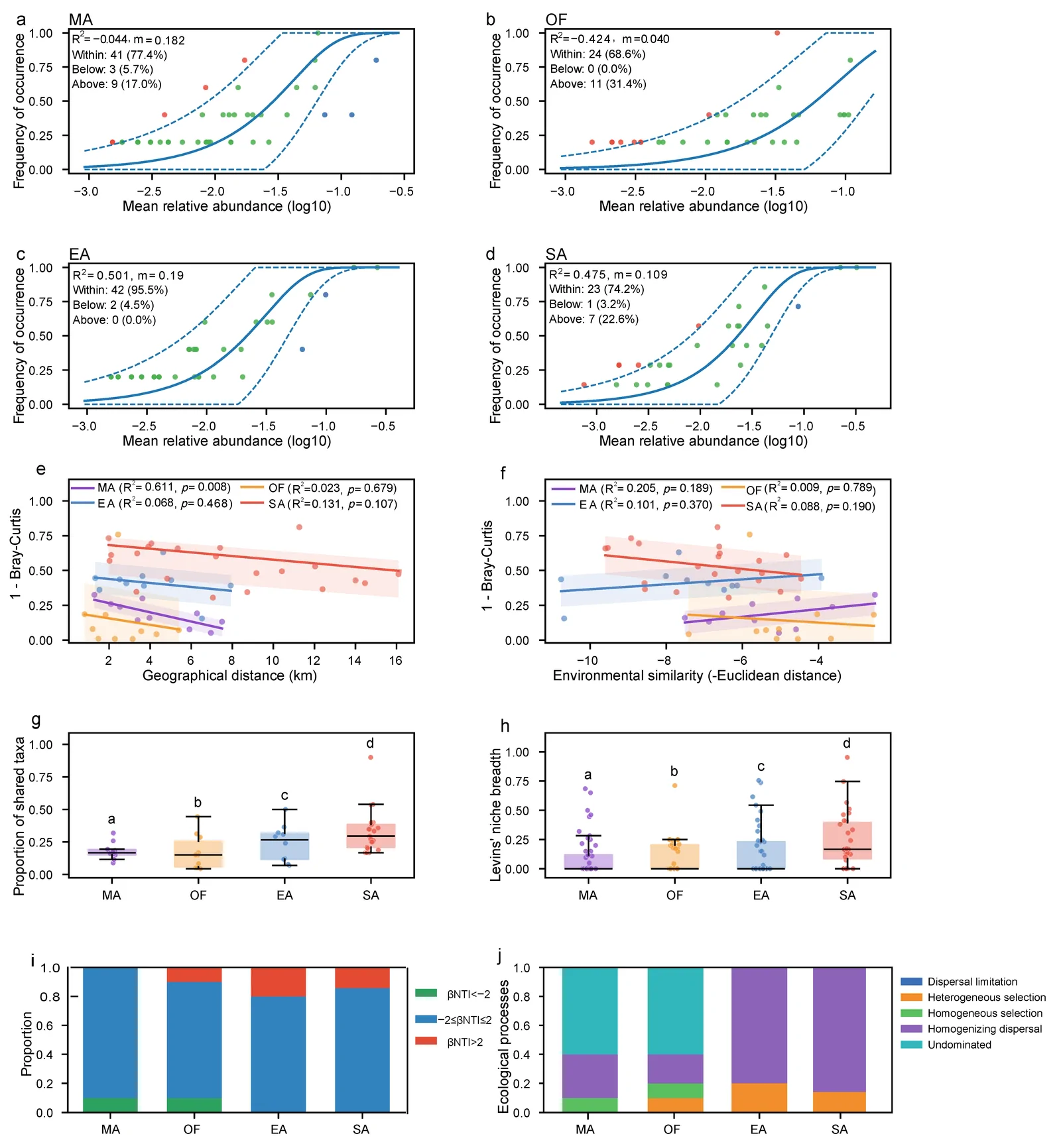
Ecological assembly mechanisms of intertidal macrobenthic communities inferred from species abundance data. The four habitat types were muddy sediment area (MA), oyster farming area (OF), eastern rocky reef area (EA), and southern rocky reef area (SA). (**a**–**d**) Neutral community model fits for MA, OF, EA, and SA, respectively, showing the relationship between species mean relative abundance and occurrence frequency. Solid blue lines indicate fitted neutral expectations, and dashed blue lines indicate 95% confidence intervals. The blue, red, and green points represent species occurring within, below, and above neutral expectations, respectively. The coefficient of determination (R^2^) and migration rate (m) are shown in each panel. (**e**) Geographic distance decay relationships between community similarity and geographical distance. (**f**) Environmental distance–decay relationships between community similarity and environmental similarity. Colored lines indicate fitted regressions, and shaded areas indicate 95% confidence intervals. (**g**) Proportion of shared taxa based on Jaccard similarity. (**h**) Levins’ niche breadth across habitat types. Different letters indicate significant differences among habitat types. (**i**) Relative proportions of βNTI categories. (**j**) Relative contributions of ecological assembly processes inferred from null-model partitioning based on βNTI and RCbray. Dispersal limitation is retained in the legend as one of the five process categories but had a zero contribution in all four habitats.

Overall, community assembly patterns differed among intertidal habitats (Figure 7; Note S5). Sloan neutral-model fits were stronger in EA and SA (R^2^ = 0.501 and 0.475, respectively) than in MA and OF, with OF showing the lowest estimated migration rate; all habitats contained taxa occurring both above and below neutral expectations, indicating contributions from both neutral and non-neutral processes (Figure 7a–d and Figure S17; Table S25). Geographic distance–decay relationships also varied among habitats: a significant relationship was detected only in MA (slope = −0.033, R^2^ = 0.611, *p* = 0.008), whereas those in OF, EA, and SA were nonsignificant, and environmental similarity was unrelated to community similarity in all habitats (Figure 7e,f; Table S26). Across all sample pairs, neither geographic distance nor environmental similarity showed a strong overall relationship with community similarity (Figure S21). Shared-taxon proportions and standardized Levins niche breadth were highest in SA (Figure 7g,h; Tables S27, S29 and S30), while *Littoraria melanostoma*, *Lunella granulata*, *Saccostrea cucullata*, and *Nodilittorina pyramidalis* exhibited the broadest niche breadths (Figure S21). Most taxonomic-proxy βNTI values fell between −2 and 2, consistent with a substantial contribution of stochastic processes (Figure 7i and Figure S18; Tables S31 and S32). Joint βNTI-proxy and RCbray partitioning further showed that undominated processes prevailed in MA and OF, whereas homogenizing dispersal dominated EA and SA (Figure 7j, Figures S19 and S20; Tables S27 and S28). Additional assembly diagnostics were consistent with these habitat-specific patterns (Figure S22).

### 3.6. Co-Occurrence Network Topology and Stability

**Figure 8:**
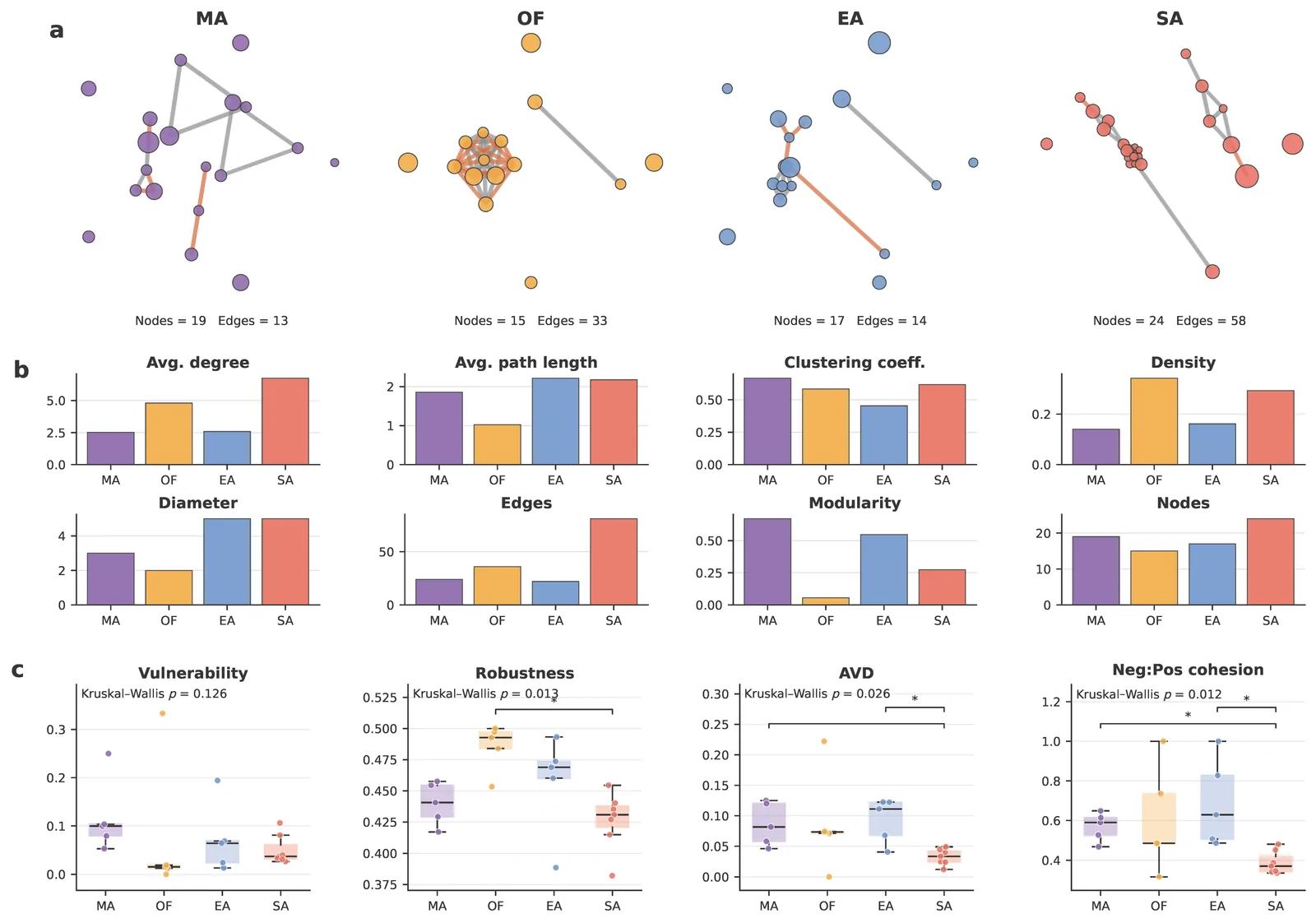
Habitat-specific co-occurrence network topology and stability of intertidal macrobenthic communities based on species abundance data. The four habitat types were muddy sediment area (MA), oyster farming area (OF), eastern rocky reef area (EA), and southern rocky reef area (SA). (**a**) Species co-occurrence networks of macrobenthic communities in MA, OF, EA, and SA. Nodes represent species, and edges represent significant pairwise associations inferred from species abundance data. Node colors correspond to habitat types (MA, purple; OF, orange; EA, blue; SA, red), while gray and orange edges indicate positive and negative associations, respectively. The numbers below each network indicate the total numbers of nodes and edges. (**b**) Comparisons of major network topological properties among habitat types, including average degree, average path length, clustering coefficient, density, diameter, edge number, modularity, and node number. (**c**) Comparisons of stability-related indices among habitat types, including vulnerability, robustness, average variation degree (AVD), and the negative-to-positive cohesion ratio (Neg:Pos cohesion). Points represent individual estimates. Overall among-habitat differences were evaluated using Kruskal-Wallis tests, and horizontal brackets indicate significant pairwise comparisons. * indicates *p* < 0.05.

Overall, species-level co-occurrence networks showed clear habitat-associated differences in topology and stability-related properties (Figure 8; Note S6). The MA, OF, EA, and SA networks contained 19, 15, 17, and 24 nodes and 13, 33, 14, and 58 edges, respectively, with SA forming the largest and most connected network and EA having the fewest edges; positive associations predominated in SA, whereas MA contained similar numbers of positive and negative edges (Figure 8a and Figure S23; Table S33). Network topology also varied among habitats: SA had the highest average degree and more highly connected nodes, OF the highest density, and MA the highest modularity and clustering coefficient (Figure 8b; Table S33). Most taxa were peripheral nodes, although SA contained more nodes with relatively high participation coefficients, and the identities and degrees of highly connected taxa differed among habitats (Figures S24, S26 and S27). Stability-related metrics also varied among habitats (Figure 8c): robustness, average variation degree (AVD), and the negative-to-positive cohesion ratio differed significantly, whereas vulnerability did not (Tables S34 and S35). Robustness was highest in OF, followed by SA, MA, and EA (Figure S25), with OF significantly exceeding MA and SA; AVD and the negative-to-positive cohesion ratio also differed significantly between SA and both MA and EA (Table S35). Overall, greater network complexity was not consistently associated with greater stability across all metrics.

### 3.7. Integrated PLS-SEM Reveals Pathways Linking Habitat Heterogeneity, Community Assembly and Network Stability

**Figure 9:**
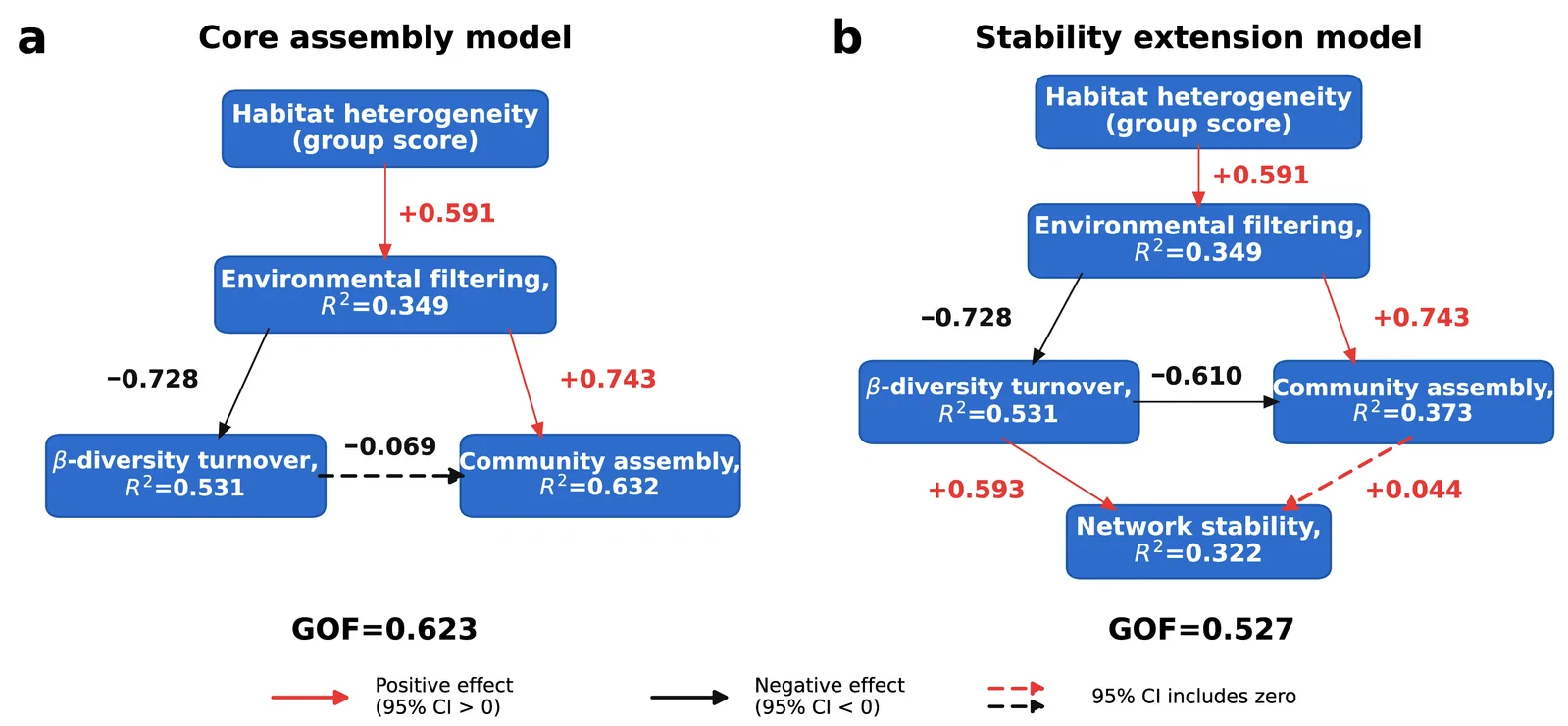
Partial least squares structural equation models linking habitat heterogeneity, environmental filtering, β-diversity turnover, community assembly and network stability. (**a**) Core assembly model showing the pathways from habitat heterogeneity to environmental filtering, β-diversity turnover and community assembly. (**b**) Stability extension model showing the additional pathways linking β-diversity turnover and community assembly to network stability. Numbers on arrows indicate standardized path coefficients, and R^2^ values in boxes indicate the proportion of variance explained for each endogenous variable. Red and black arrows indicate positive and negative effects, respectively. Solid arrows indicate paths whose 95% confidence intervals did not include zero, whereas dashed arrows indicate paths whose 95% confidence intervals included zero. GOF indicates the goodness-of-fit of each PLS-SEM model.

PLS-SEM identified associations linking habitat heterogeneity with community assembly and potential network stability through environmental filtering and β-diversity turnover (Figure 9; Note S7). In the core assembly model, habitat heterogeneity was positively associated with environmental filtering (β = 0.591, *p* = 0.004), which was positively associated with community assembly (β = 0.743, *p* = 0.002) and negatively associated with β-diversity turnover (β = −0.728, *p* < 0.001; Table S36); the direct association between turnover and assembly was nonsignificant. In the stability-extension model, turnover was negatively associated with community assembly (β = −0.610, *p* = 0.003) but positively associated with potential network stability (β = 0.593, *p* = 0.022), whereas community assembly showed no significant direct association with stability (β = 0.044, *p* = 0.856). Candidate-model comparison and predictive evaluation supported the selected structures, with the core model showing the highest overall goodness of fit (Figure S28; Tables S37 and S38), and bootstrap confidence intervals supported the significant direct pathways (Figure S29). Construct reliability, indicator loadings, and latent-score distributions varied among constructs and habitats (Table S39; Figures S30 and S31). Significant indirect associations further indicated that habitat heterogeneity was associated with turnover and assembly through environmental filtering, while environmental filtering was indirectly associated with potential network stability through β-diversity turnover (Figure S32; Table S40).

## 4. Discussion

Habitat heterogeneity around Nan’ao Island was expressed more strongly through community composition than through local alpha diversity. Similar observed richness and evenness across habitats co-occurred with marked changes in species identity, functional richness, assembly proxies, and co-occurrence organization. This distinction matters because local richness alone would portray the four habitats as relatively similar, while beta diversity showed substantial ecological complementarity. The results therefore support the proposed emphasis on abundance reorganization and species replacement, rather than a uniform habitat-driven increase in local diversity.

Substrate, hydrodynamics, tidal exposure, nutrients, and aquaculture disturbance can alter attachment space, burrowing conditions, food supply, and environmental stress [5,12,60,61]. Consistent with these mechanisms, dominant taxa varied among MA, OF, EA, and SA, while depth and correlated temperature, nutrient, and sediment gradients were associated with compositional structure. However, constrained ordinations were nonsignificant and habitat type retained explanatory power beyond the measured variables. The observed habitat separation should therefore be interpreted as a joint signal of measured environmental gradients, substrate identity, spatial context, and unmeasured habitat attributes, rather than the effect of any single driver.

Turnover dominated nestedness, indicating habitat-specific replacement, rather than a single gradient from species-rich to species-poor sites [51,62,63]. Muddy sediments favor burrowing and deposit-feeding taxa, rocky shores provide attachment surfaces and crevices, and oyster-farming areas combine biogenic structure, biodeposition, and disturbance. These contrasting filters can preserve different assemblages even when richness is comparable. Accordingly, the conservation value of the intertidal mosaic lies partly in regional complementarity: losing one habitat type may remove distinct taxa and ecological strategies without producing a large initial decline in richness at the remaining sites.

Assembly analyses provide compatible, but necessarily tentative, process evidence. Oyster farming represents an anthropogenically modified habitat in which biogenic shell structure, biodeposition, localized organic enrichment, and recurrent culture-related disturbance may simultaneously alter substrate complexity, food availability, and environmental filtering. In the present study, OF was characterized mainly by undominated assembly processes, suggesting that no single inferred process clearly prevailed. One possible explanation is that the combination of artificial structural complexity and spatially variable aquaculture disturbance generates multiple weak and interacting filters [64,65]. In contrast, the greater contribution of homogenizing dispersal in EA and SA may reflect stronger hydrodynamic exchange and recolonization among connected rocky substrates. The significant distance–decay relationship confined to MA further indicates that spatial structuring was habitat-dependent. These contrasts suggest that aquaculture modification and natural reef connectivity may be associated with different assembly regimes. However, because βNTI-related calculations were based on species abundance, rather than a phylogenetic tree, and because the study represents a single-season survey, these assembly categories should be regarded as species-level process proxies and the proposed mechanisms as hypotheses, rather than direct evidence of aquaculture effects or phylogenetic selection [55,56,66].

Functional responses were narrower than taxonomic responses. FRic changed among habitats, but FEve, FDiv, FDis, RaoQ, and FRed did not, suggesting that habitat differences altered the boundaries of occupied trait space more than its internal distribution. Taxonomic turnover can, therefore, coexist with partial functional buffering when replacement taxa perform similar feeding, mobility, or habitat-use roles [44,45,67]. This decoupling cautions against assuming that strong compositional change necessarily entails proportional loss of ecological functions.

Co-occurrence networks also reorganized across habitats, but topology and stability-related metrics did not vary in parallel. SA had the largest network and highest average degree, OF had the highest density and robustness, and MA had the highest modularity and clustering. Network size or connectivity is, therefore, not a sufficient proxy for stability [15,68,69,70]. Moreover, correlation edges can arise from shared environmental responses and do not establish competition, facilitation, or trophic interaction [16,71,72]. The positive PLS-SEM association between turnover and potential network stability is most reasonably interpreted as evidence that species replacement covaries with network reorganization, not that turnover causally increases stability.

The exploratory PLS-SEM synthesized these patterns into a testable conceptual pathway. Habitat grouping was associated with environmental filtering, filtering with assembly proxies and turnover, and turnover with potential network stability, whereas assembly had no direct association with stability. This result shifts attention from a simple ‘assembly determines stability’ narrative toward the reorganization of species identities across habitats [73,74]. Nevertheless, feedback and shared predictors cannot be separated with cross-sectional data; path signs and magnitudes are conditional on the specified model, and should guide future tests, rather than be treated as definitive mechanisms [18,75,76].

Several limitations constrain inference. First, one May 2024 survey cannot represent seasonal recruitment, mortality, monsoon forcing, extreme events, tidal variation, or changes in aquaculture activity; the results describe spatial structure during the sampled period only [13,77]. Second, 22 sites were divided unequally among four habitats, which limits power and increases uncertainty in multivariable models, within-habitat networks, and PLS-SEM. Resampling, cross-validation, effect sizes, and confidence intervals reduce, but do not eliminate, this limitation. Third, habitat type and geographic position were partly confounded, tidal-zone observations were pooled for habitat-level analyses, quadrat effort differed between soft and rocky substrates, and some sediment measurements used nearby subtidal proxies, rather than paired intertidal samples. Accordingly, sediment variables were treated only as broad site-level proxies for substrate background and should not be interpreted as direct measurements of intertidal sediment conditions. Differences in hydrodynamic exposure, sediment sorting, and resuspension between intertidal and subtidal environments may, therefore, introduce uncertainty into sediment-related associations. Fourth, correlation networks do not identify biological interactions, and abundance-based βNTI proxies lack phylogenetic resolution. Seasonal replication, balanced sampling, standardized effort, paired sediment collection, phylogenetic information, and independent interaction data are needed to test the temporal stability and causal direction of the proposed framework.

## 5. Conclusions

Intertidal habitat heterogeneity around Nan’ao Island was associated primarily with species replacement, selective functional change, assembly shifts, and network reorganization, rather than consistent changes in local richness. Management should therefore retain muddy, aquaculture-modified, and rocky shores as distinct, but complementary, planning units; avoid using alpha diversity alone to prioritize sites; and include beta-turnover, FRic, and stability-related network indicators in coastal monitoring. Restoration should preserve substrate and hydrodynamic gradients instead of homogenizing shoreline structure. Because the evidence comes from one season and a limited, unbalanced sample, repeated seasonal surveys and standardized long-term sampling are required before the inferred pathways are used for causal prediction.

## Data Availability

The original contributions presented in this study are included in the article/Supplementary Material. Further inquiries can be directed to the corresponding author.

## Supplementary Materials

The following supporting information can be downloaded at: https://www.mdpi.com/article/10.3390/ani16162630/s1.
