# Supplementary material for: Habitat Heterogeneity Drives Intertidal Macrobenthic Community Differentiation via Environmental Filtering, Species Turnover, and Ecological Network Reorganization
Source: Animals (Basel). 2026 Aug 21;16(16):2630. doi: 10.3390/ani16162630 (PMC13510014; doi:10.3390/ani16162630)
Supplement: Supplementary file 1 [file animals-16-02630-s001.zip › animals-4424921-supplementary.pdf]

Supplementary Materials for

Habitat Heterogeneity Drives Intertidal Macrobenthic Community Differentiation via Environmental Filtering, Species Turnover, and Ecological Network Reorganization

Jiujiang Wang [1,] Wuhan Lin [1], Junyan Cai 1, Zhiyang Tang [1], Qiyun Zhang [1], Yanping Chen [1], Ziming He [1], Wenhua Liu [1,2,3] and Zonghang Zhang [1,2,*]

1 Guangdong Provincial Key Laboratory of Marine Biotechnology, Institute of Marine Sciences, Shantou University, Shantou 515063, China; 23jjwang@stu.edu.cn (J.W.); gdlinwuhan@163.com (W.L.); 24jycai@stu.edu.cn (J.C.); 24zytang@stu.edu.cn (Z.T.); 21qyzhang@stu.edu.cn (Q.Z.); 25ypchen@stu.edu.cn (Y.C.); 25zmhe1@stu.edu.cn (Z.H.); whliu@stu.edu.cn (W.L.)

2 International Joint Research Center for Marine Ecological Protection and Disaster Prevention, Shantou University, Shantou 515063, China

3 College of Fisheries, Guangdong Ocean University, Zhanjiang 524088, China

* Correspondence: zhangzh@stu.edu.cn

This file includes:

Notes S1-7

Tables S1-S40

Figures S1-S32

Note S1. Community structure of macrobenthic assemblages.

Macrobenthic community composition differed among the four habitat groups (Figs. S1–S4; Table S1). MA had the highest species richness (53 species) and the greatest number of unique species (22), whereas SA showed the highest total and mean site densities but the lowest species richness (31 species). Mollusca and Gastropoda dominated all groups. The most abundant genera were *Littoraria, Nodilittorina, Monodonta, Siphonaria and Mytilus, while Littoraria melanostoma and Nodilittorina pyramidalis* were the two dominant species (Tables S2–S3). Species composition also showed clear spatial heterogeneity among sampling sites.

At the genus level, *Amphibalanus, Nodilittorina* and *Varuna* showed nominal differences among habitat groups before multiple-testing correction (Fig. S5; Table S4), although none remained significant after Benjamini–Hochberg correction. Pairwise comparisons indicated that *Nodilittorina* and *Siphonaria* had lower relative abundances in MA than in EA (Table S5), whereas most other dominant genera showed no significant pairwise differences.

Note S2. Patterns of macrobenthic taxonomic and functional diversity.

Alpha diversity showed limited variation among the four habitat types. Overall Kruskal–Wallis tests indicated significant differences in Chao1 (H = 9.146, P = 0.027) and ACE (H = 8.078, P = 0.044), whereas observed richness showed a marginal difference (P = 0.053). Shannon diversity, Simpson diversity and Pielou evenness did not differ significantly among habitats (Table S6). MA generally exhibited the highest observed and estimated richness, followed by EA, whereas OF and SA showed comparatively lower values (Table S7).

Despite the significant overall differences in Chao1 and ACE, no pairwise comparison remained significant after Benjamini–Hochberg correction (Table S8). Large effect sizes were nevertheless observed for several richness comparisons, particularly between MA and OF and between OF and EA. None of the relationships between alpha-diversity indices and environmental variables remained significant after multiple-testing correction. The strongest positive correlations were observed between NH_4–N and ACE (Spearman's ρ = 0.564) and between NH_4–N and Chao1 (ρ = 0.521), but both were nonsignificant after correction (Table S9).

Functional richness differed significantly among the four habitat types (FRic: Kruskal–Wallis H = 12.312, P = 0.006), whereas functional evenness, functional divergence, Rao's quadratic entropy, functional dispersion and functional redundancy showed no significant differences (Table S10). FRic was highest in MA, followed by OF, EA and SA, with median values of 0.1090, 0.0668, 0.0195 and 0.0207, respectively (Table S11). Functional redundancy tended to be higher in EA and SA, but the overall difference was not significant.

Pairwise comparisons showed that FRic was significantly higher in MA than in SA (BH-adjusted P = 0.015) and higher in OF than in SA (BH-adjusted P = 0.035), with large effect sizes. No significant pairwise differences were detected for the other functional-diversity indices (Table S12). Species traits were assigned using either exact taxonomic matches or proxy taxa when species-level trait information was unavailable (Table S13).

Note S3. Beta-diversity differentiation among intertidal habitats.

Pairwise PERMANOVA showed a significant difference in community composition only between MA and SA after Benjamini–Hochberg correction (adjusted $P = 0.036$; Fig. S6). UPGMA clustering indicated partial aggregation of sites with similar communities, but the four habitat types were not completely separated (Fig. S7). The NMDS Shepard plot had a high stress value of 0.508, suggesting that the two-dimensional ordination should be interpreted cautiously (Fig. S8). Jaccard-based PCoA explained 23.0% and 12.2% of the variation along the first two axes and showed considerable overlap among habitats. Between-habitat Bray–Curtis dissimilarity and turnover were higher than their corresponding within-habitat values, whereas nestedness was consistently low (Fig. S9).

Depth, total nitrogen and nitrate showed the strongest associations with beta-diversity ordination, followed by temperature, silicate and sediment grain-size variables (Fig. S10). Distances to group centroids were greatest in OF and lowest in SA (Table S14), with significant pairwise differences between MA and SA, OF and EA, and OF and SA (Table S15). Site-level NMDS and PCoA scores are provided in Tables S16 and S17. Overall, mean Bray–Curtis dissimilarity was higher between habitats than within habitats (0.730 vs. 0.634), mainly because of the greater turnover component (0.623 vs. 0.555), while nestedness differed little between the two comparison types (Table S18).

Note S4. Environmental filtering and key drivers of community variation.

Univariate screening identified depth, total nitrogen, silicate, temperature, nitrite, nitrate, height and latitude as significant predictors of community variation, with depth explaining the largest proportion of variation ($R^2 = 0.197$, $P = 0.003$; Table S19). However, the overall RDA and dbRDA models were not significant, whereas habitat type significantly explained 25.5% of community variation in PERMANOVA ($P = 0.019$; Table S20). After collinearity filtering, all retained variables had VIF values below 10 (Table S21). Forward selection retained depth as the primary significant predictor, while the additional contribution of temperature was not significant (Table S22). Random-forest analysis also ranked depth as the most important variable, followed by temperature and sediment grain-size parameters (Table S23). Both classification models showed moderate predictive performance, with balanced logistic regression slightly outperforming balanced random forest in balanced accuracy and Macro-F1 score (Table S24).

The correlation matrix revealed strong covariation among several sediment grain-size variables and moderate correlations among nutrient and physical variables (Fig. S11). Sequential filtering sharply reduced the maximum VIF to below the threshold of 10 (Fig. S12), while forward-selection results further confirmed the dominant explanatory contribution of depth (Fig. S13). Random-forest importance patterns were consistent with the univariate screening, although the confusion matrix indicated substantial classification overlap, particularly between some rocky-reef habitats (Fig. S14). Environmental variables showed distinct axis-specific correlations with the Bray–Curtis PCoA axes, with depth, temperature, nutrients and sediment characteristics contributing most strongly to community ordination patterns (Fig. S15).

Note S5. Ecological assembly processes across habitats.

The Sloan neutral model showed relatively good fits for EA and SA (R^2 = 0.501 and 0.475), whereas the fits for MA and OF were weak, with OF also exhibiting the lowest estimated migration rate (Table S25). A significant geographic distance–decay relationship was detected only in MA (slope = −0.033, R^2 = 0.611, P = 0.008), while environmental similarity was unrelated to community similarity in all groups (Table S26). Shared-taxon proportions and Levins' niche breadth were highest in SA, whereas undominated processes prevailed in MA and OF and homogenizing dispersal dominated EA and SA (Tables S27–S28). SA had significantly higher shared-taxon proportions than MA and OF (Table S29) and significantly greater niche breadth than all other groups (Table S30). Most pairwise taxonomic-proxy βNTI values occurred between −2 and 2, indicating that stochastic processes were generally important (Tables S31–S32).

Neutral-model patterns confirmed the high proportion of taxa within neutral expectations in EA and the weaker model fit in MA and OF (Fig. S16). The taxonomic-proxy βNTI and RCbray matrices revealed pronounced variation in assembly patterns among site pairs (Figs. S17–S18), while ecological-process classification showed that undominated processes were concentrated mainly in MA and OF and homogenizing dispersal was more frequent in EA and SA (Fig. S19). Across all site pairs, neither geographical distance nor environmental similarity showed a strong overall relationship with community similarity (Fig. S20). Littoraria melanostoma, Lunella granulata, Saccostrea cucullata and Nodilittorina pyramidalis exhibited the broadest standardized niche breadths (Fig. S21).

Note S6. Co-occurrence network topology and stability.

Co-occurrence network properties differed markedly among habitat types (Table S33). SA contained the largest and most highly connected network, with 24 nodes, 81 edges and the highest average degree (6.75), whereas EA had the fewest edges and the lowest robustness. OF exhibited the highest network density and robustness and the lowest vulnerability, while MA showed the highest modularity and clustering coefficient. Overall tests detected significant differences in robustness, average vulnerability degree (AVD) and the negative-to-positive cohesion ratio, but not in vulnerability (Table S34). Pairwise comparisons showed higher robustness in OF than in MA and SA, while AVD and the negative-to-positive cohesion ratio differed significantly between SA and both MA and EA (Table S35).

Positive and negative edges occurred in similar proportions in MA, whereas positive associations predominated strongly in SA, which also had the lowest negative-to-positive cohesion ratio (Fig. S22). Most taxa were classified as peripheral nodes, although SA contained more nodes with relatively high participation coefficients, suggesting stronger inter-module connectivity (Fig. S23). Network robustness was highest in OF, followed by SA, MA and EA (Fig. S24). The identities of highly connected taxa differed among habitats, with SA supporting taxa of substantially higher degree than the other groups (Fig. S25). Degree distributions further showed relatively sparse networks in MA and EA, a distinct low- and high-degree structure in OF, and the broadest distribution toward high connectivity in SA (Fig. S26).

Note S7. Integrated PLS-SEM reveals pathways linking habitat heterogeneity, community assembly and network stability.

PLS-SEM analyses showed that habitat heterogeneity positively affected environmental filtering ($\beta = 0.591$, $P = 0.004$), which was negatively associated with β-diversity turnover ($\beta = -0.728$, $P < 0.001$; Table S36). In the core assembly model, environmental filtering directly promoted community assembly ($\beta = 0.743$, $P = 0.002$), whereas the path from β-diversity turnover to assembly was nonsignificant. In the stability-extension model, β-diversity turnover negatively affected community assembly ($\beta = -0.610$, $P = 0.003$) but positively affected network stability ($\beta = 0.593$, $P = 0.022$); community assembly had no direct effect on stability. Candidate-model screening favored the model containing direct environmental and β-diversity pathways (Table S37), while the core assembly model showed the highest overall goodness of fit and predictive performance (Table S38). Construct reliability was generally highest for β-diversity turnover, although several constructs showed low internal-consistency estimates and should be interpreted cautiously (Table S39). Significant indirect effects included habitat heterogeneity → environmental filtering → β-diversity turnover, habitat heterogeneity → environmental filtering → community assembly, and environmental filtering → β-diversity turnover → network stability (Table S40).

Model comparisons and goodness-of-fit values supported the relative performance of the selected core and stability-extension models (Fig. S27). Bootstrap confidence intervals confirmed the significant paths from habitat heterogeneity to environmental filtering, from environmental filtering to β-diversity turnover and assembly, and from β-diversity turnover to assembly and network stability, while the direct assembly–stability pathway remained nonsignificant (Fig. S28). The standardized latent-variable scores varied markedly among habitat types, particularly for environmental filtering, β-diversity turnover, community assembly and network stability (Fig. S29). Indicator loadings differed in direction and magnitude among constructs, with consistently strong loadings for the β-diversity turnover indicators (Fig. S30). Bootstrap analysis of indirect effects further demonstrated that habitat heterogeneity primarily influenced turnover and assembly through environmental filtering, whereas environmental filtering affected network stability indirectly through β-diversity turnover (Fig. S31).

Table S1. Summary of macrobenthic taxonomic richness and abundance among the four habitat types.

Group	No. site	Total density	Total count	Species richness	Genus richness	Family richness	Mean site density	Mean site species richness
MA	5	6608	413	53	49	34	1321.600	17.000
OF	5	7504	469	35	31	24	1500.800	11.200
EA	5	8656	541	44	39	25	1731.200	15.400
SA	7	15440	965	31	28	21	2205.714	12.714

Table S2. The 10 most dominant macrobenthic genera across all sampling sites.

Overall rank within rank	Taxon	Density	Occurrence sites	Relative abundance percent
1	Littoraria	9728	18	25.461
2	Nodilittorina	6608	16	17.295
3	Monodonta	2688	15	7.035
4	Siphonaria	2624	11	6.868
5	Mytilus	2320	15	6.072
6	Saccostrea	2096	18	5.486
7	Tetraclita	1232	6	3.224
8	Perinereis	1168	13	3.057
9	Thais	1088	9	2.848
10	Cellana	784	13	2.052

Table S3. The 10 most dominant macrobenthic species across all sampling sites.

Overall rank within rank	Taxon	Density	Occurrence sites	Relative abundance percent
1	*Littoraria melanostoma*	8768	16	22.948
2	*Nodilittorina pyramidalis*	6608	16	17.295
3	*Monodonta labio*	2688	15	7.035
4	*Siphonaria japonica*	2480	11	6.491
5	*Mytilus coruscus*	2288	13	5.988
6	*Saccostrea cucullata*	1456	15	3.811
7	*Tetraclita japonica*	1232	6	3.224
8	*Thais luteostoma*	1088	9	2.848
9	*Perinereis aibuhitensis*	1072	13	2.806
10	*Littoraria undulata*	960	4	2.513

Table S4. Kruskal–Wallis test results for candidate genera differing among the four habitat types.

Taxon	H	P value	Padj	Epsilon squared	Mean relative abundance percent
Amphibalanus	12.046	0.007	0.304	0.503	1.560
Nodilittorina	10.246	0.017	0.348	0.403	13.696
Varuna	8.880	0.031	0.433	0.327	1.168
Neanthes	5.907	0.116	0.638	0.162	2.429
Ligia	5.551	0.136	0.638	0.142	1.494

Table S5. Selected pairwise comparisons of dominant genera between habitat types.

Comparison	Genus	Mean group1 percent	Mean group2 percent	Difference group1 minus group2 percent	ci95_low	ci95_high	P value
EA vs SA	Tentidonax	6.317	0.000	6.317	0.000	18.317	0.105
EA vs SA	Mytilus	3.518	8.798	-5.280	-11.788	0.528	0.252
EA vs SA	Nodilittorina	17.048	22.466	-5.418	-14.097	3.954	0.343
EA vs SA	Siphonaria	10.713	4.962	5.751	-2.289	14.843	0.362
EA vs SA	Littoraria	27.220	32.273	-5.053	-23.964	15.020	0.639
MA vs EA	Nodilittorina	1.989	17.048	-15.059	-21.387	-9.450	0.011
MA vs EA	Siphonaria	0.732	10.713	-9.981	-17.645	-2.887	0.045
MA vs EA	Monodonta	18.938	3.213	15.725	1.013	33.980	0.139
MA vs EA	Tentidonax	0.000	6.317	-6.317	-18.317	0.000	0.180
MA vs EA	Littoraria	19.537	27.220	-7.683	-33.768	18.530	0.402
MA vs OF	Monodonta	18.938	10.513	8.426	-15.510	31.076	0.236
MA vs OF	Nodilittorina	1.989	9.774	-7.785	-19.241	2.143	0.724

Table S6. Overall comparisons of six macrobenthic alpha-diversity indices among the four habitat types.

Metric	Preferred test	Preferred statistic	Preferred P	Significance
Observed richness (Sobs)	Kruskal-Wallis	7.670	0.053	ns
Chao1	Kruskal-Wallis	9.146	0.027	*
ACE	Kruskal-Wallis	8.078	0.044	*
Shannon	Kruskal-Wallis	3.284	0.350	ns
Simpson (1-D)	Kruskal-Wallis	1.029	0.794	ns
Pielou	Kruskal-Wallis	0.315	0.957	ns

Table S7. Descriptive statistics of macrobenthic alpha-diversity indices within each habitat type.

Metric	Group	n	Mean	SD	Median	Q1	Q3
Observed richness (Sobs)	MA	5	17.000	3.082	17.000	17.000	19.000
Observed richness (Sobs)	OF	5	11.200	2.168	12.000	9.000	12.000
Observed richness (Sobs)	EA	5	15.400	1.817	15.000	15.000	16.000
Observed richness (Sobs)	SA	7	12.714	4.424	11.000	9.500	15.500
Chao1	MA	5	30.566	13.995	29.569	23.264	37.003
Chao1	OF	5	13.186	2.859	12.497	11.968	15.984
Chao1	EA	5	29.834	25.766	19.193	18.968	20.218
Chao1	SA	7	14.489	7.045	11.000	10.076	17.074
ACE	MA	5	28.876	11.380	28.308	27.068	31.484
ACE	OF	5	13.839	3.658	12.500	12.146	14.845
ACE	EA	5	25.434	17.684	18.373	17.714	19.138
ACE	SA	7	14.924	7.760	11.145	10.182	17.218
Shannon	MA	5	2.153	0.239	2.199	1.990	2.319
Shannon	OF	5	1.879	0.250	1.855	1.807	2.067
Shannon	EA	5	2.100	0.446	2.117	1.818	2.268
Shannon	SA	7	1.878	0.349	1.907	1.591	2.134
Simpson (1-D)	MA	5	0.807	0.080	0.821	0.748	0.872
Simpson (1-D)	OF	5	0.783	0.064	0.786	0.756	0.823
Simpson (1-D)	EA	5	0.806	0.118	0.839	0.787	0.860
Simpson (1-D)	SA	7	0.769	0.103	0.808	0.707	0.845
Pielou	MA	5	0.769	0.123	0.747	0.664	0.855
Pielou	OF	5	0.782	0.075	0.783	0.727	0.844
Pielou	EA	5	0.768	0.143	0.782	0.709	0.838
Pielou	SA	7	0.750	0.079	0.753	0.688	0.797

Table S8. Pairwise comparisons of macrobenthic alpha-diversity indices among habitat types.

Metric	Group1	Group2	Median1	Median2	BH adjusted p	Cliffs delta	Significance
Observed richness (Sobs)	MA	OF	17.000	12.000	0.100	0.840	ns
Observed richness (Sobs)	MA	EA	17.000	15.000	0.352	0.440	ns
Observed richness (Sobs)	MA	SA	17.000	11.000	0.237	0.571	ns
Observed richness (Sobs)	OF	EA	12.000	15.000	0.100	-0.920	ns
Observed richness (Sobs)	OF	SA	12.000	11.000	0.868	-0.086	ns
Observed richness (Sobs)	EA	SA	15.000	11.000	0.352	0.429	ns
Chao1	MA	OF	29.569	12.497	0.063	0.840	ns
Chao1	MA	EA	29.569	19.193	0.657	0.280	ns
Chao1	MA	SA	29.569	11.000	0.063	0.829	ns
Chao1	OF	EA	12.497	19.193	0.063	-0.840	ns
Chao1	OF	SA	12.497	11.000	0.755	0.143	ns
Chao1	EA	SA	19.193	11.000	0.223	0.543	ns
ACE	MA	OF	28.308	12.500	0.095	0.840	ns
ACE	MA	EA	28.308	18.373	0.657	0.280	ns
ACE	MA	SA	28.308	11.145	0.095	0.771	ns
ACE	OF	EA	12.500	18.373	0.190	-0.680	ns
ACE	OF	SA	12.500	11.145	0.755	0.143	ns
ACE	EA	SA	18.373	11.145	0.223	0.543	ns
Shannon	MA	OF	2.199	1.855	0.606	0.600	ns
Shannon	MA	EA	2.199	2.117	0.829	0.200	ns
Shannon	MA	SA	2.199	1.907	0.606	0.486	ns
Shannon	OF	EA	1.855	2.117	0.648	-0.360	ns
Shannon	OF	SA	1.855	1.907	0.876	-0.086	ns
Shannon	EA	SA	2.117	1.907	0.648	0.314	ns

Table S9. Spearman correlations between macrobenthic alpha-diversity indices and environmental variables.

Metric	Variable	Spearman rho	BH adjusted p	Significance
ACE	NH4-N	0.564	0.207	ns
Chao1	NH4-N	0.521	0.427	ns
Chao1	GS_Mode	-0.257	0.742	ns
Chao1	GS_Mean	-0.204	0.742	ns
Chao1	GS_D25	-0.196	0.742	ns
Chao1	GS_D10	-0.278	0.742	ns
Chao1	GS_D50	-0.239	0.742	ns
Chao1	GS_Median	-0.239	0.742	ns
Chao1	depthCV	0.245	0.742	ns
Chao1	S	-0.277	0.742	ns
Chao1	TN	0.220	0.742	ns
Chao1	NO3-N	0.223	0.742	ns
Chao1	NO2-N	0.356	0.742	ns
Chao1	pH	0.233	0.742	ns
Chao1	T	-0.219	0.742	ns
Chao1	GS_CV	0.312	0.742	ns
Chao1	GS_D90	-0.196	0.742	ns
Chao1	GS_D75	-0.209	0.742	ns
ACE	GS_Mode	-0.257	0.753	ns
ACE	GS_Median	-0.222	0.753	ns
ACE	GS_D25	-0.195	0.753	ns
ACE	GS_D10	-0.285	0.753	ns
ACE	GS_D75	-0.205	0.753	ns
ACE	GS_CV	0.326	0.753	ns

Table S10. Overall comparisons of six functional-diversity indices among the four habitat types.

Metric	Preferred test	Preferred statistic	Preferred P	Significance
FRic	Kruskal-Wallis	12.3122	0.0064	**
FEve	Kruskal-Wallis	3.3544	0.3401	ns
FDiv	Kruskal-Wallis	2.4925	0.4766	ns
RaoQ	Kruskal-Wallis	3.1443	0.3699	ns
FDis	Kruskal-Wallis	3.8626	0.2767	ns
FRed	Kruskal-Wallis	6.3005	0.0979	ns

Table S11. Descriptive statistics of functional-diversity indices within each habitat type.

Metric	Group	n	Mean	SD	Median	Q1	Q3
FRic	MA	5	0.1075	0.0144	0.1090	0.0984	0.1206
FRic	OF	5	0.0718	0.0260	0.0668	0.0643	0.0840
FRic	EA	5	0.0461	0.0470	0.0195	0.0142	0.0959
FRic	SA	7	0.0243	0.0213	0.0207	0.0093	0.0349
FEve	MA	5	0.4692	0.0905	0.4942	0.4134	0.5250
FEve	OF	5	0.5186	0.1504	0.4979	0.4536	0.5553
FEve	EA	5	0.3558	0.1333	0.3661	0.2330	0.4223
FEve	SA	7	0.4420	0.0565	0.4422	0.4212	0.4809
FDiv	MA	5	0.7566	0.0788	0.7793	0.6816	0.7896
FDiv	OF	5	0.8047	0.1469	0.8141	0.7007	0.9353
FDiv	EA	5	0.7285	0.1164	0.7971	0.6829	0.8048
FDiv	SA	7	0.6935	0.0737	0.7060	0.6482	0.7204
RaoQ	MA	5	0.5189	0.0936	0.4617	0.4484	0.5915
RaoQ	OF	5	0.4920	0.0807	0.4944	0.4635	0.4970
RaoQ	EA	5	0.4329	0.0924	0.4798	0.3659	0.4822
RaoQ	SA	7	0.4176	0.0731	0.4519	0.3538	0.4654
FDis	MA	5	0.3331	0.0793	0.3091	0.2757	0.4061
FDis	OF	5	0.3095	0.0835	0.3159	0.2759	0.3643
FDis	EA	5	0.2669	0.1000	0.2742	0.1882	0.2916
FDis	SA	7	0.2566	0.0325	0.2586	0.2400	0.2794
FRed	MA	5	0.3590	0.0713	0.3645	0.3353	0.3826
FRed	OF	5	0.3681	0.1144	0.3427	0.3301	0.4249
FRed	EA	5	0.4638	0.0739	0.4297	0.4283	0.4992
FRed	SA	7	0.4574	0.0543	0.4487	0.4322	0.4509

Table S12. Matching between observed macrobenthic taxa and taxa used as sources of functional-trait information.

Metric	Group1	Group2	Median1	Median2	BH adjusted p	Cliffs delta	Significance
FRic	MA	OF	0.1090	0.0668	0.0635	0.8400	ns
FRic	MA	EA	0.1090	0.0195	0.0833	0.7600	ns
FRic	MA	SA	0.1090	0.0207	0.0152	1.0000	*
FRic	OF	EA	0.0668	0.0195	0.5048	0.3600	ns
FRic	OF	SA	0.0668	0.0207	0.0350	0.9143	*
FRic	EA	SA	0.0195	0.0207	0.6389	0.2000	ns
FEve	MA	OF	0.4942	0.4979	0.8413	-0.1200	ns
FEve	MA	EA	0.4942	0.3661	0.6190	0.4400	ns
FEve	MA	SA	0.4942	0.4422	0.6364	0.2571	ns
FEve	OF	EA	0.4979	0.3661	0.6190	0.6000	ns
FEve	OF	SA	0.4979	0.4422	0.6364	0.3143	ns
FEve	EA	SA	0.3661	0.4422	0.6190	-0.4286	ns
FDiv	MA	OF	0.7793	0.8141	0.7667	-0.2800	ns
FDiv	MA	EA	0.7793	0.7971	0.8413	-0.1200	ns
FDiv	MA	SA	0.7793	0.7060	0.7667	0.3143	ns
FDiv	OF	EA	0.8141	0.7971	0.7667	0.4400	ns
FDiv	OF	SA	0.8141	0.7060	0.7667	0.4857	ns
FDiv	EA	SA	0.7971	0.7060	0.7667	0.2000	ns
RaoQ	MA	OF	0.4617	0.4944	1.0000	-0.0400	ns
RaoQ	MA	EA	0.4617	0.4798	0.6571	0.2800	ns
RaoQ	MA	SA	0.4617	0.4519	0.6571	0.4286	ns
RaoQ	OF	EA	0.4944	0.4798	0.6571	0.3600	ns
RaoQ	OF	SA	0.4944	0.4519	0.6571	0.5429	ns
RaoQ	EA	SA	0.4798	0.4519	0.6571	0.2571	ns

Table S13. Pairwise comparisons of functional-diversity indices among the four habitat types.

Observed taxon	Trait source taxon	Match type
Alpheus distinguendus	*Alpheus distinguendus*	Exact
Amphibalanus amphitrite	*Balanus amphitrite*	Proxy
Ampithoe valida	*Ampithoe valida*	Exact
Anadara broughtonii	*Scapharca broughtonii*	Proxy
Anomalodiscus squamosus	*Anomalodiscus squamosus*	Exact
Arca boucardi	*Arca boucardi*	Exact
Arcuatula senhousia	*Arcuatula senhousia*	Exact
Arenicola brasiliensis	*Arenicola brasiliensis*	Exact
Balanoglossus sp.	*Balanoglossus sp.*	Exact
Balanus albicostatus	*Balanus albicostatus*	Exact
Brachidontes variabilis	*Brachidontes variabilis*	Exact
Cantharidus sp.	*Cantharus cecillei*	Proxy
Capitulum mitella	*Capitulum mitella*	Exact
Carcinoplax sp.	*Carcinoplax sp.*	Exact
Cellana toreuma	*Cellana toreuma*	Exact
Chion dysoni	*Chion dysoni*	Exact
Chion semigranosus	*Chion dysoni*	Proxy
Circe scripta	*Circe scripta*	Exact
Crassostrea ariakensis	*Magallana ariakensis*	Proxy
Cyclina sinensis	*Cyclina sinensis*	Exact
Cyclosunetta menstrualis	*Cyclosunetta menstrualis*	Exact
Donax cuneatus	*Tentidonax kiusiuensis*	Proxy
Epixanthus frontalis	*Epixanthus frontalis*	Exact
Gaetice depressus	*Gaetice depressus*	Exact

Table S14. Site-level distances to habitat-group centroids in multivariate community space.

Site	Group	Distance to centroid
S01	MA	0.4284367517128605
S02	MA	0.5427848243189399
S03	MA	0.3536595736862714
S04	MA	0.3452871511480595
S05	MA	0.4423588024439039
S06	OF	0.5945306568726967
S07	OF	0.5406019490991152
S08	OF	0.47091565610440195
S09	OF	0.5316558502598013
S10	OF	0.4686193645375157
S11	EA	0.36150405282030545
S12	EA	0.2305598935406008
S13	EA	0.2645490342674811
S14	EA	0.4010911661205331
S15	EA	0.15082891097767917
S16	SA	0.33751952146314396
S17	SA	0.3725534061214859
S18	SA	0.1443800620694038
S19	SA	0.2814600867099969
S20	SA	0.08179999572753381
S21	SA	0.14769549636161972
S22	SA	0.1493135944005086

Table S15. Pairwise comparisons of distances to group centroids among the four habitat types.

Group 1	Group 2	U	p	p adj BH	Significance
MA	OF	4	0.09523809523809523	0.11428571428571428	ns
MA	EA	21	0.09523809523809523	0.11428571428571428	ns
MA	SA	33	0.010101010101010102	0.020202020202020204	*
OF	EA	25	0.007936507936507936	0.020202020202020204	*
OF	SA	35	0.0025252525252525255	0.015151515151515152	*
EA	SA	25	0.26767676767676774	0.26767676767676774	ns

Table S16. Site scores from the two-dimensional NMDS ordination based on Bray–Curtis dissimilarity.

Site	Group	NMDS1	NMDS2
S01	MA	0.2488750813360762	0.19297157555568908
S02	MA	-0.458759609	-0.121247407
S03	MA	0.5112111597030855	-0.704672804
S04	MA	0.2238757148229744	-0.953221095
S05	MA	-0.269560573	-0.932534886
S06	OF	-0.444607907	-1.04494164
S07	OF	1.0320607570677534	-0.756899188
S08	OF	0.41279980303374764	-1.131570622
S09	OF	-0.197119704	0.5300532265453584
S10	OF	-0.087922629	0.32419079527905215
S11	EA	0.13244167644808408	0.7299516579370856
S12	EA	-0.229579205	0.3713771391598874
S13	EA	0.10468220068027957	0.33488769995443113
S14	EA	-0.198320169	-0.003396509
S15	EA	-0.200878341	0.26759648212627934
S16	SA	0.10747056295876538	0.2028971036308272
S17	SA	0.20702490949803476	0.47410981800766205
S18	SA	-0.108631851	0.5323560607578444
S19	SA	-0.246520017	0.5525065403909265
S20	SA	-0.121258349	0.3261068449706958
S21	SA	-0.265867409	0.41962968953347857
S22	SA	-0.151416104	0.3898495163910445

Table S17. Site scores from the principal coordinate analysis based on Jaccard dissimilarity.

Site	Group	PCoA1	PCoA2
S01	MA	0.039352888725629774	0.2764684932562038
S02	MA	-0.103872732	0.07829553142459272
S03	MA	-0.424613892	0.022298023942435036
S04	MA	-0.517069374	-0.007722891
S05	MA	-0.466956449	-0.225404323
S06	OF	-0.520023906	-0.185668237
S07	OF	-0.480670004	0.1393115994127724
S08	OF	-0.500872474	0.016330310825433746
S09	OF	0.32899546599333257	-0.179031648
S10	OF	0.2568779882121697	-0.059929377
S11	EA	0.21280133913289054	0.24358326554022247
S12	EA	0.2346114476270721	-0.101520309
S13	EA	0.1694074072551616	0.28848880369160246
S14	EA	0.017673415727500925	-0.161661513
S15	EA	0.19759366013333224	-0.079242507
S16	SA	0.07888977977860716	0.2713175606525633
S17	SA	0.1596736184080097	0.3161614132430753
S18	SA	0.28380329807526605	-0.1208213
S19	SA	0.3120980529394342	-0.22629904
S20	SA	0.22588346398590617	-0.038368784
S21	SA	0.2363722227808273	-0.126986518
S22	SA	0.2600447811728421	-0.139598554

Table S18. Summary statistics of within- and between-habitat beta-diversity components.

Metric	Pair_type	Mean	Median	Std	Count
Bray-Curtis	Between	0.7298642730245734	0.7727495107632094	0.22035697648143704	180
Bray-Curtis	Within	0.6341366699693626	0.6101694915254238	0.2243271646054133	51
Nestedness	Between	0.06477001628763916	0.04761904761904767	0.05996882819549953	180
Nestedness	Within	0.06246899236708148	0.0431034482758621	0.06507109235934223	51
Turnover	Between	0.6234282505038439	0.6666666666666666	0.22898888228225675	180
Turnover	Within	0.5549233043678 5	0.5555555555555556	0.1989586075547 6247	51

Table S19. Top environmental variables from univariate screening.

Variable	Univariate R^2	Permutation p	Significance
depth	0.1971	0.0033	**
TN	0.1901	0.0033	**
SIO3-Si	0.1542	0.0033	**
T	0.1446	0.0067	**
NO2-N	0.1248	0.0067	**
NO3-N	0.1836	0.0100	*
height	0.1265	0.0133	*
Latitude	0.1200	0.0167	*

Table S20. Constrained ordination and PERMANOVA summary.

Model	R^2	Adjusted R^2	Permutation p	Significance
RDA	0.4257	0.0723	0.1650	ns
dbRDA	0.4097	0.0464	0.2780	ns
PERMANOVA_group	0.2546	nan	0.0190	*

Table S21. Final VIF values.

**Variable**	**VIF**
Latitude	6.5450
SIO3-Si	3.9256
T	3.7485
GS_SD	3.6531
NO2-N	3.5145
northward	3.3208
GS_Mode	3.2359
depth	3.1487

Table S22. Forward-selection path.

Step	Candidate	Incremental R^2	Cumulative R^2	Cumulative adjusted R^2	Permutation p
1	depth	0.1971	0.1971	0.1570	0.0033
2	T	0.0474	0.2445	0.1650	0.2633

Table S23. Random-forest variable importance.

Variable	Importance	Permutation importance mean	Permutation importance SD
depth	0.1140	0.1140	0.0203
T	0.0904	0.0904	0.0159
GS_SD	0.0851	0.0851	0.0119
GS_Mode	0.0797	0.0797	0.0168
Latitude	0.0737	0.0737	0.0159
NO2-N	0.0656	0.0656	0.0107
SIO3-Si	0.0650	0.0650	0.0128
northward	0.0302	0.0302	0.0086

Table S24. LOOCV classification performance.

Model	Balanced accuracy	Macro-F1	Accuracy	Cohen kappa
Balanced random forest	0.5286	0.4955	0.5455	0.3785
Balanced logistic	0.5571	0.5472	0.5455	0.3923

Table S25. Sloan neutral model summary.

Group	No. samples	No. taxa	Migration rate m	R2	Within percent	Above percent	Below percent
MA	5.000	53.000	0.182	-0.044	77.358	16.981	5.660
OF	5.000	35.000	0.040	-0.424	68.571	31.429	0.000
EA	5.000	44.000	0.191	0.501	95.455	0.000	4.545
SA	7.000	31.000	0.109	0.475	74.194	22.581	3.226

Table S26. Distance-decay regression summary.

Distance type	Group	No. pairs	Slope	Intercept	R^2	p
Geographic distance	MA	10.000	-0.033	0.332	0.611	0.008
Geographic distance	OF	10.000	-0.023	0.203	0.023	0.679
Geographic distance	EA	10.000	-0.014	0.468	0.068	0.468
Geographic distance	SA	21.000	-0.012	0.625	0.131	0.107
Environmental similarity	MA	10.000	0.027	0.332	0.205	0.189
Environmental similarity	OF	10.000	-0.017	0.060	0.009	0.789
Environmental similarity	EA	10.000	0.018	0.543	0.101	0.370
Environmental similarity	SA	21.000	-0.027	0.347	0.088	0.190

Table S27. Group-level assembly summary.

Group	Mean shared taxa proportion	Mean Levins Bst	Mean βNTI proxy	Dominant process
MA	0.180	0.096	-0.180	Undominated
OF	0.174	0.101	0.331	Undominated
EA	0.240	0.133	0.605	Homogenizing dispersal
SA	0.332	0.243	0.634	Homogenizing dispersal

Table S28. Ecological-process proportions by group.

Group	Ecological process	Count	Proportion	Total pairs
MA	Dispersal limitation	0.000	0.000	10.000
MA	Heterogeneous selection	0.000	0.000	10.000
MA	Homogeneous selection	1.000	0.100	10.000
MA	Homogenizing dispersal	3.000	0.300	10.000
MA	Undominated	6.000	0.600	10.000
OF	Dispersal limitation	0.000	0.000	10.000
OF	Heterogeneous selection	1.000	0.100	10.000
OF	Homogeneous selection	1.000	0.100	10.000
OF	Homogenizing dispersal	2.000	0.200	10.000
OF	Undominated	6.000	0.600	10.000
EA	Dispersal limitation	0.000	0.000	10.000
EA	Heterogeneous selection	2.000	0.200	10.000
EA	Homogeneous selection	0.000	0.000	10.000
EA	Homogenizing dispersal	8.000	0.800	10.000
EA	Undominated	0.000	0.000	10.000
SA	Dispersal limitation	0.000	0.000	21.000
SA	Heterogeneous selection	3.000	0.143	21.000
SA	Homogeneous selection	0.000	0.000	21.000
SA	Homogenizing dispersal	18.000	0.857	21.000
SA	Undominated	0.000	0.000	21.000

Table S29. Pairwise tests for shared taxa proportions.

Group1	Group2	n1	n2	Mean1	Mean2	U	Raw p	BH adjusted p	Significance
MA	OF	10	10	0.180	0.174	61.000	0.427	0.496	ns
MA	EA	10	10	0.180	0.240	40.500	0.496	0.496	ns
MA	SA	10	21	0.180	0.332	29.000	0.001	0.008	**
OF	EA	10	10	0.174	0.240	35.000	0.273	0.409	ns
OF	SA	10	21	0.174	0.332	40.500	0.007	0.020	*
EA	SA	10	21	0.240	0.332	71.000	0.157	0.313	ns

Table S30. Pairwise tests for Levins niche breadth.

Group 1	Group 2	n1	n2	Mean1	Mean2	U	Raw p	BH adjusted p	Significance
MA	OF	53	35	0.096	0.101	866.500	0.556	0.673	ns
MA	EA	53	44	0.096	0.133	1096.000	0.561	0.673	ns
MA	SA	53	31	0.096	0.243	446.000	0.000	0.001	**
OF	EA	35	44	0.101	0.133	773.000	0.978	0.978	ns
OF	SA	35	31	0.101	0.243	352.000	0.011	0.023	*
EA	SA	44	31	0.133	0.243	441.500	0.007	0.020	*

Table S31. betaNTI-class proportions by group.

Group	βNTI class	Count	Proportion	Total pairs
MA	βNTI<-2	1.000	0.100	10.000
MA	-2≤βNTI≤2	9.000	0.900	10.000
MA	βNTI>2	0.000	0.000	10.000
OF	βNTI<-2	1.000	0.100	10.000
OF	-2≤βNTI≤2	8.000	0.800	10.000
OF	βNTI>2	1.000	0.100	10.000
EA	βNTI<-2	0.000	0.000	10.000
EA	-2≤βNTI≤2	8.000	0.800	10.000
EA	βNTI>2	2.000	0.200	10.000
SA	βNTI<-2	0.000	0.000	21.000
SA	-2≤βNTI≤2	18.000	0.857	21.000
SA	βNTI>2	3.000	0.143	21.000

Table S32. Example rows from pairwise betaNTI-style and RCbray results.

Site 1	Site 2	Group 1	Group 2	Pair group	βNTI taxonomic proxy	βNTI class	RCbray	Ecological process
S01	S02	MA	MA	MA	-1.223	-2≤βNTI≤2	-0.974	Homogenizing dispersal
S01	S03	MA	MA	MA	-0.209	-2≤βNTI≤2	-0.894	Undominated
S01	S04	MA	MA	MA	0.268	-2≤βNTI≤2	-0.946	Undominated
S01	S05	MA	MA	MA	-2.579	βNTI<-2	-0.814	Homogeneous selection
S01	S06	MA	OF	Between	-2.357	βNTI<-2	-0.669	Homogeneous selection
S01	S07	MA	OF	Between	0.131	-2≤βNTI≤2	-0.661	Undominated
S01	S08	MA	OF	Between	0.247	-2≤βNTI≤2	0.006	Undominated
S01	S09	MA	OF	Between	-0.025	-2≤βNTI≤2	-0.984	Homogenizing dispersal
S01	S10	MA	OF	Between	-0.358	-2≤βNTI≤2	-1.000	Homogenizing dispersal
S01	S11	MA	EA	Between	0.532	-2≤βNTI≤2	-0.946	Undominated

Note: betaNTI and process partitioning were calculated using a taxonomy-derived distance matrix because no sequence-based phylogeny was available in the uploaded files. For strict phylogenetic betaNTI/iCAMP analysis, a rooted phylogenetic tree or representative sequences should be provided.

Table S33. Topological structure and stability metrics of macrobenthic co-occurrence networks among the four habitat types.

Group	MA	OF	EA	SA
Nodes	19	15	17	24
Edges	24	36	22	81
Avg_Degree	2.526315789	4.8	2.588235294	6.75
Avg_Path_Length	1.857142857	1.027777778	2.218181818	2.177489177
Clustering_Coeff	0.666666667	0.583333333	0.452941176	0.617255892
Density	0.140350877	0.342857143	0.161764706	0.293478261
Diameter	3	2	5	5
Modularity	0.670908907	0.055714636	0.547818095	0.274004049
Communities	6	6	5	4
Positive_Edges	12	22	14	68
Negative_Edges	12	14	8	13
Vulnerability	0.116666667	0.00312989	0.080921633	0.017726344
Robustness	0.420204082	0.49962963	0.395082645	0.43322314
AVD	0.06122449	0.108024691	0.031404959	0.015741834
Positive_Cohesion_Mean	0.574705512	0.429479241	0.3613655	0.733813872
Negative_Cohesion_Mean	0.73515049	0.425637604	0.714412203	0.470701409
NegPos_Ratio_Mean	0.569412112	0.604279754	0.689712182	0.387606627

Table S34. Overall comparisons of co-occurrence network stability metrics among habitat types.

Metric	Kruskal H	p	Significance
Vulnerability	5.715076228	0.126325866	ns
Robustness	12.76101695	0.005182984	**
AVD	9.291360813	0.02565772	*
NegPos_Ratio	11.00022586	0.011724654	*

Table S35. Pairwise comparisons of co-occurrence network stability metrics among habitat types.

Metric	Group1	Group2	n1	n2	U	Raw_p	BH_p
Vulnerability	MA	OF	5	5	20	0.150793651	0.301587302
Vulnerability	MA	EA	5	5	19	0.222222222	0.333333333
Vulnerability	MA	SA	5	7	29	0.073232323	0.301587302
Vulnerability	OF	EA	5	5	7	0.30952381	0.371428571
Vulnerability	OF	SA	5	7	7	0.106060606	0.301587302
Vulnerability	EA	SA	5	7	17	1	1
Robustness	MA	OF	5	5	0	0.007936508	0.023809524
Robustness	MA	EA	5	5	6	0.208690443	0.250428532
Robustness	MA	SA	5	7	24	0.343434343	0.343434343
Robustness	OF	EA	5	5	22	0.059327061	0.110295033
Robustness	OF	SA	5	7	35	0.002525253	0.015151515
Robustness	EA	SA	5	7	29	0.073530022	0.110295033
AVD	MA	OF	5	5	14	0.841269841	1
AVD	MA	EA	5	5	12	1	1
AVD	MA	SA	5	7	34	0.005050505	0.03030303
AVD	OF	EA	5	5	11	0.841269841	1
AVD	OF	SA	5	7	28	0.106060606	0.212121212
AVD	EA	SA	5	7	33	0.01010101	0.03030303
NegPos_Ratio	MA	OF	5	5	13	1	1
NegPos_Ratio	MA	EA	5	5	9	0.547619048	0.657142857
NegPos_Ratio	MA	SA	5	7	34	0.005050505	0.015151515
NegPos_Ratio	OF	EA	5	5	7	0.30952381	0.464285714
NegPos_Ratio	OF	SA	5	7	28	0.106060606	0.212121212
NegPos_Ratio	EA	SA	5	7	35	0.002525253	0.015151515

Table S36. Standardized path coefficients of the final core assembly and stability-extension PLS-SEM models.

Model	Predictor label	Endogenous label	Path beta	p	CI_low	CI_high	R^2	Significance
Core_assembly_model	Habitat heterogeneity	Environmental filtering	0.591	0.004	0.329	0.822	0.349	**
Core_assembly_model	Environmental filtering	β-diversity turnover	-0.728	0.000	-1.024	-0.470	0.531	***
Core_assembly_model	Environmental filtering	Community assembly	0.743	0.002	0.390	1.096	0.632	**
Core_assembly_model	β-diversity turnover	Community assembly	-0.069	0.738	-0.384	0.245	0.632	ns
Stability_extension_model	Habitat heterogeneity	Environmental filtering	0.591	0.004	0.398	0.828	0.349	**
Stability_extension_model	Environmental filtering	β-diversity turnover	-0.728	0.000	-1.108	-0.486	0.531	***
Stability_extension_model	β-diversity turnover	Community assembly	-0.610	0.003	-0.928	-0.377	0.373	**
Stability_extension_model	β-diversity turnover	Network stability	0.593	0.022	0.299	1.093	0.322	*
Stability_extension_model	Community assembly	Network stability	0.044	0.856	-0.397	0.618	0.322	ns

Table S37. Comparison of candidate structural equation models based on AICc and explained variance.

Model	sum_AICc	mean_adjR2	n_equations
M4_env_beta_directs	220.053	0.407	4
M3_beta_direct_stability	226.093	0.354	4
M1_beta_assembly_stability	229.582	0.305	4
M2_tax_func_beta_to_assembly	350.710	0.281	6
M5_diversity_direct_stability	352.762	0.288	6

Table S38. Model fit and leave-one-out cross-validation performance of the final PLS-SEM models.

Model	n_paths	sum_AICc	mean_R2	mean_AdjR2	GOF	Mean_communality	Mean_R2
Core_assembly_model	4	-31.466	0.504	0.472	0.623	0.770	0.504
Stability_extension_model	5	-23.640	0.393	0.354	0.527	0.706	0.393
Diversity_parallel_model	8	-23.890	0.324	0.281	0.462	0.660	0.324

Model	Endogenous	LOOCV_R2	LOOCV_RMSE	n_predicted
Core_assembly_model	ENV	0.245	0.869	22
Core_assembly_model	BETA	0.423	0.760	22
Core_assembly_model	ASSEMBLY	0.516	0.696	22
Stability_extension_model	ENV	0.245	0.869	22
Stability_extension_model	BETA	0.423	0.760	22
Stability_extension_model	ASSEMBLY	0.257	0.862	22
Stability_extension_model	STABILITY	0.097	0.950	22
Diversity_parallel_model	ENV	0.245	0.869	22
Diversity_parallel_model	TAX	-0.264	1.124	22
Diversity_parallel_model	FUNC	0.313	0.829	22
Diversity_parallel_model	BETA	0.423	0.760	22
Diversity_parallel_model	ASSEMBLY	0.285	0.846	22
Diversity_parallel_model	STABILITY	-0.071	1.035	22

Table S39. Measurement-model reliability and validity statistics for the latent constructs.

Construct code	Construct	No. indicators	Cronbach alpha	Composite reliability	AVE
HH	Habitat heterogeneity	1	nan	nan	1.000
ENV	Environmental filtering	10	-1.466	0.930	0.578
TAX	Taxonomic diversity	6	0.701	0.850	0.523
FUNC	Functional diversity	6	0.033	0.876	0.569
BETA	β-diversity turnover	2	0.957	0.979	0.959
ASSEMBLY	Community assembly	5	-0.433	0.853	0.543
STABILITY	Network stability	4	0.233	0.753	0.448

Table S40. Bootstrapped indirect effects in the core assembly and stability-extension models.

Model	Effect	Boot_mean	CI_low	CI_high	CI_includes_zero
Core_assembly_model	HH→ENV→BETA	-0.442	-0.708	-0.176	False
Core_assembly_model	ENV→BETA→ASSEMBLY	0.078	-0.168	0.456	True
Core_assembly_model	HH→ENV→ASSEMBLY	0.435	0.150	0.782	False
Core_assembly_model	HH→ENV→BETA→ASSEMBLY	0.037	-0.150	0.248	True
Stability_extension_model	ENV→BETA→STABILITY	-0.436	-0.888	-0.096	False
Stability_extension_model	BETA→ASSEMBLY→STABILITY	-0.058	-0.456	0.220	True

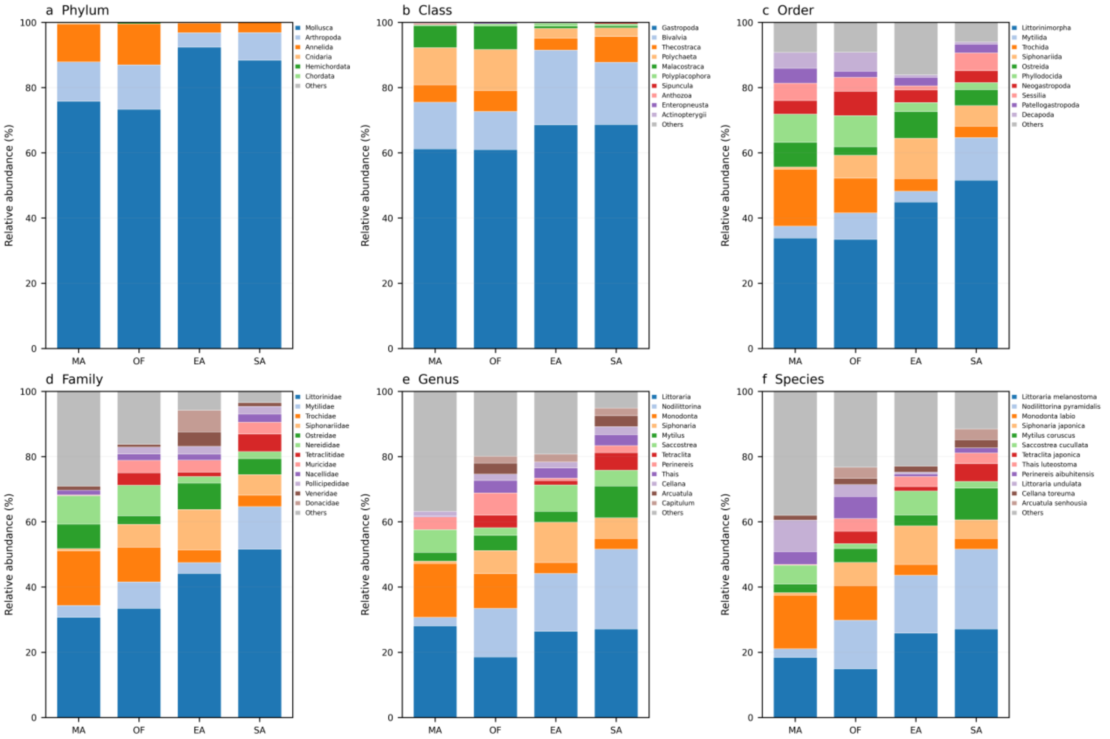

Figure S1. Taxonomic composition of macrobenthic communities among the four habitat types. Stacked bars show the relative abundance of dominant taxa at the (a) phylum, (b) class, (c) order, (d) family, (e) genus and (f) species levels. Taxa with relatively low abundances were grouped as "Others." MA, muddy sediment area; OF, oyster farming area; EA, eastern rocky reef area; SA, southern rocky reef area.

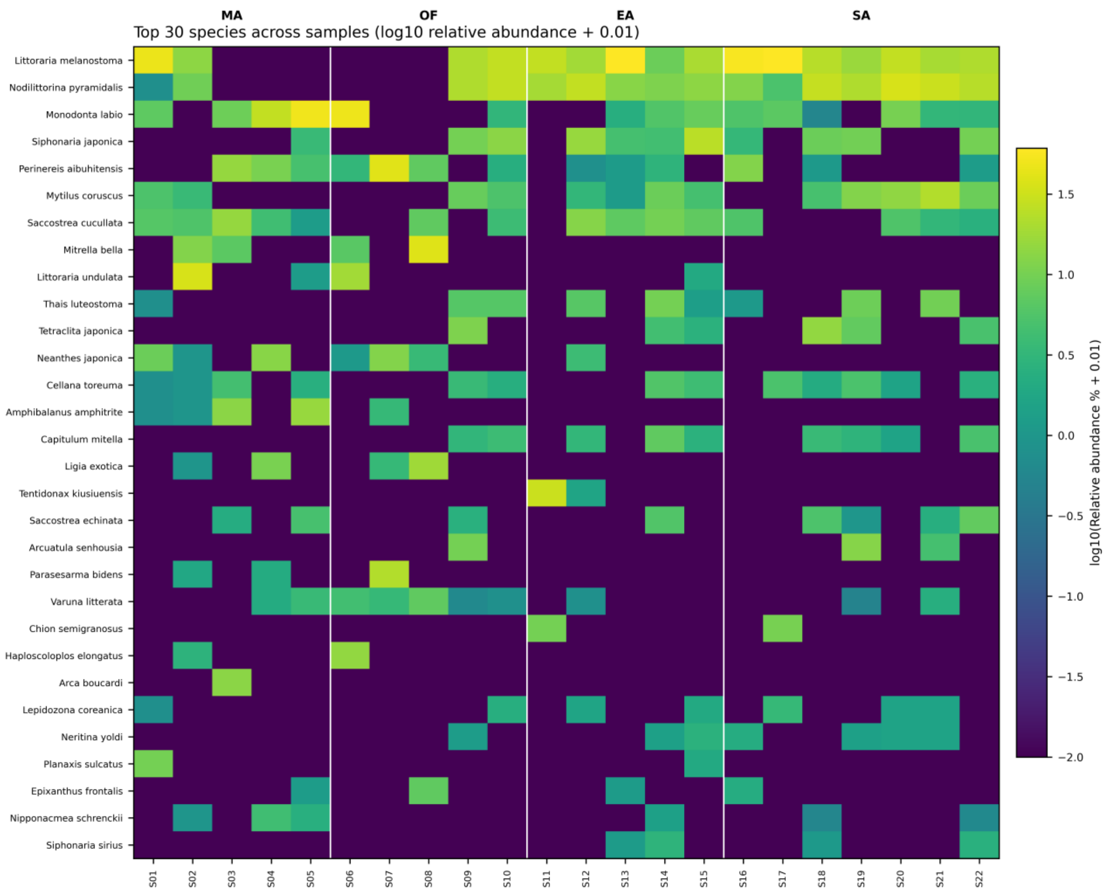

Figure S2. Distribution of the 30 most abundant macrobenthic species across sampling sites. The heatmap shows log-transformed relative abundance values [log10(relative abundance [%] + 0.01)]. Sampling sites are arranged according to the four habitat types, and white vertical lines indicate habitat boundaries.

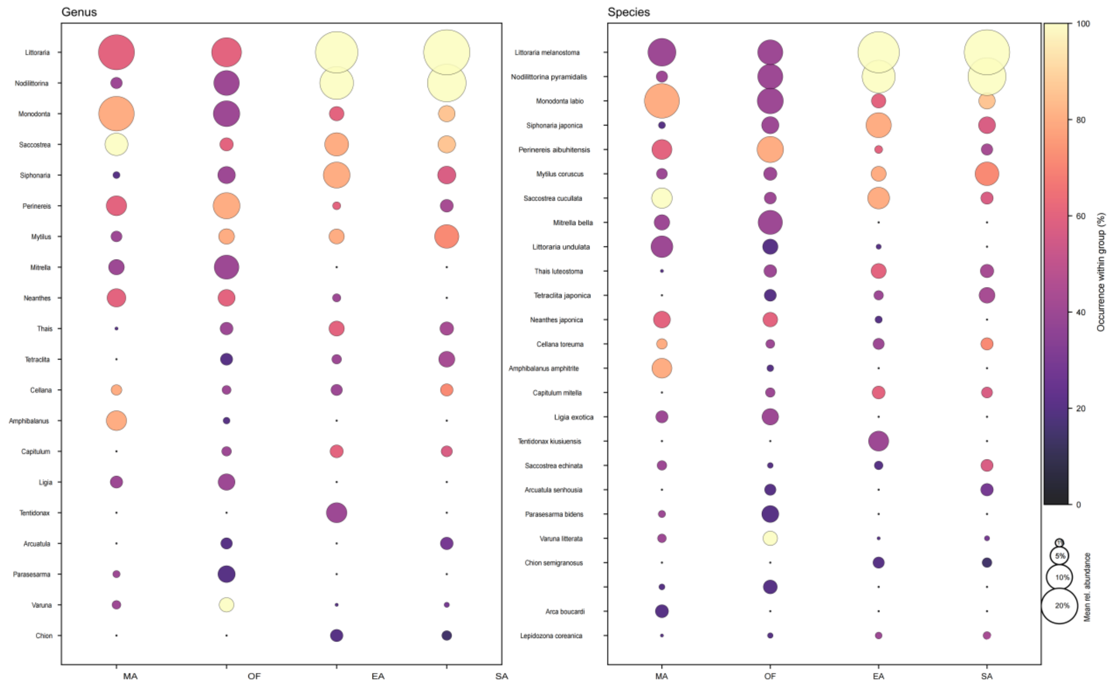

Figure S3. Distribution patterns of dominant macrobenthic genera and species among the four habitat types. Bubble size represents mean relative abundance, whereas bubble color indicates the occurrence frequency of each taxon within each habitat type. The left and right panels show genus- and species-level patterns, respectively.

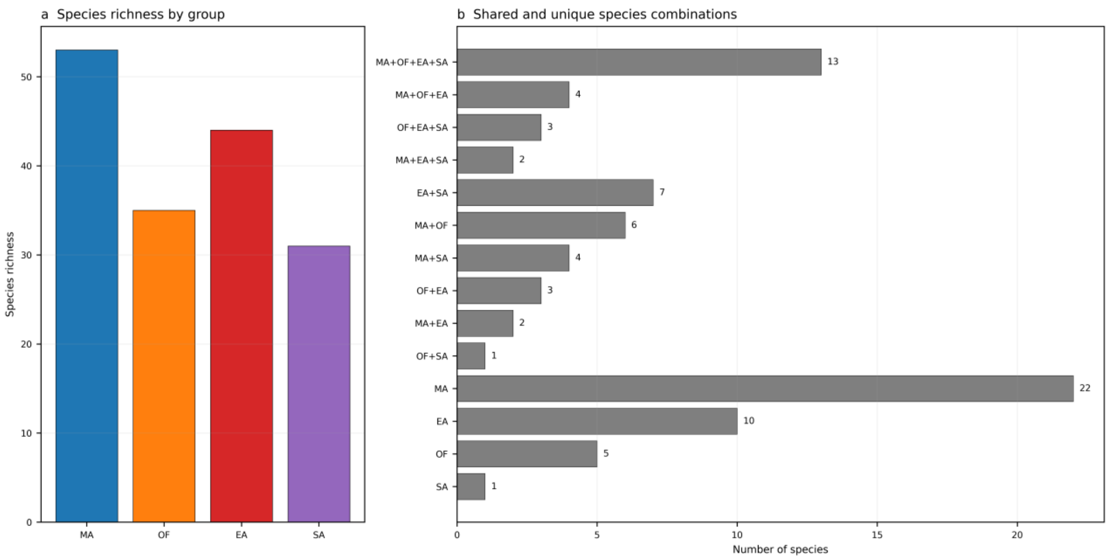

Figure S4. Species richness and species-sharing patterns among the four habitat types. (a) Total species richness recorded in each habitat type. (b) Numbers of unique and shared species associated with different combinations of habitat types.

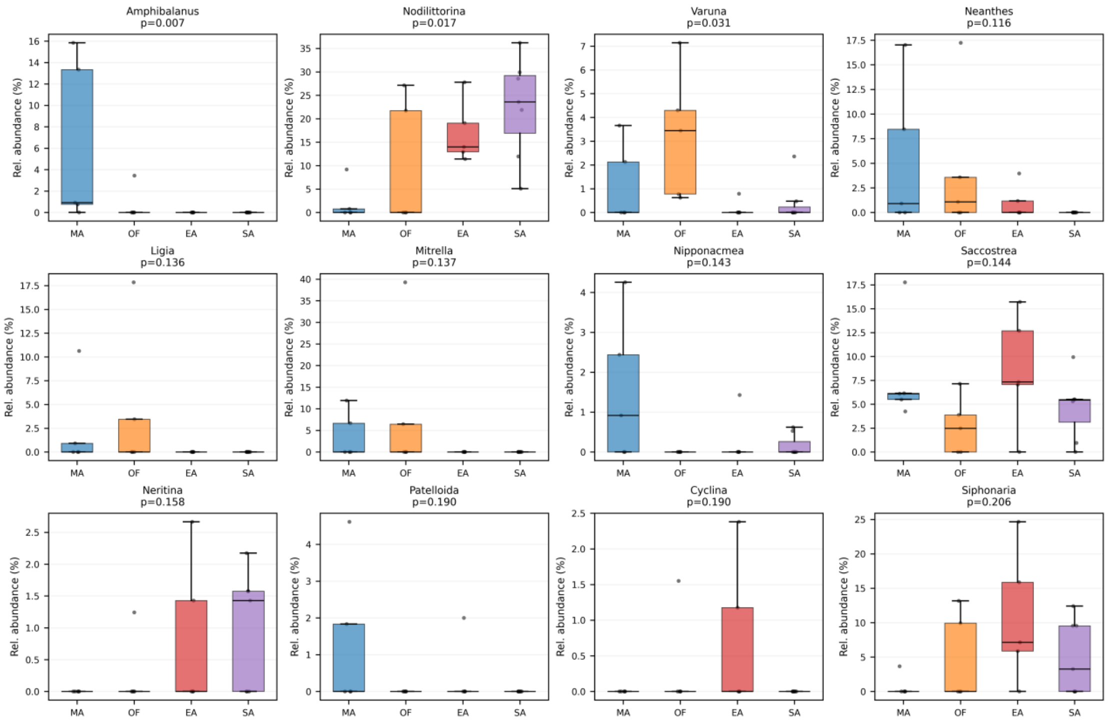

Figure S5. Relative abundance distributions of selected macrobenthic genera among the four habitat types. Boxes represent the interquartile range, horizontal lines indicate medians, whiskers show the data range and points represent individual sampling sites. The displayed p-values were obtained using Kruskal–Wallis tests.

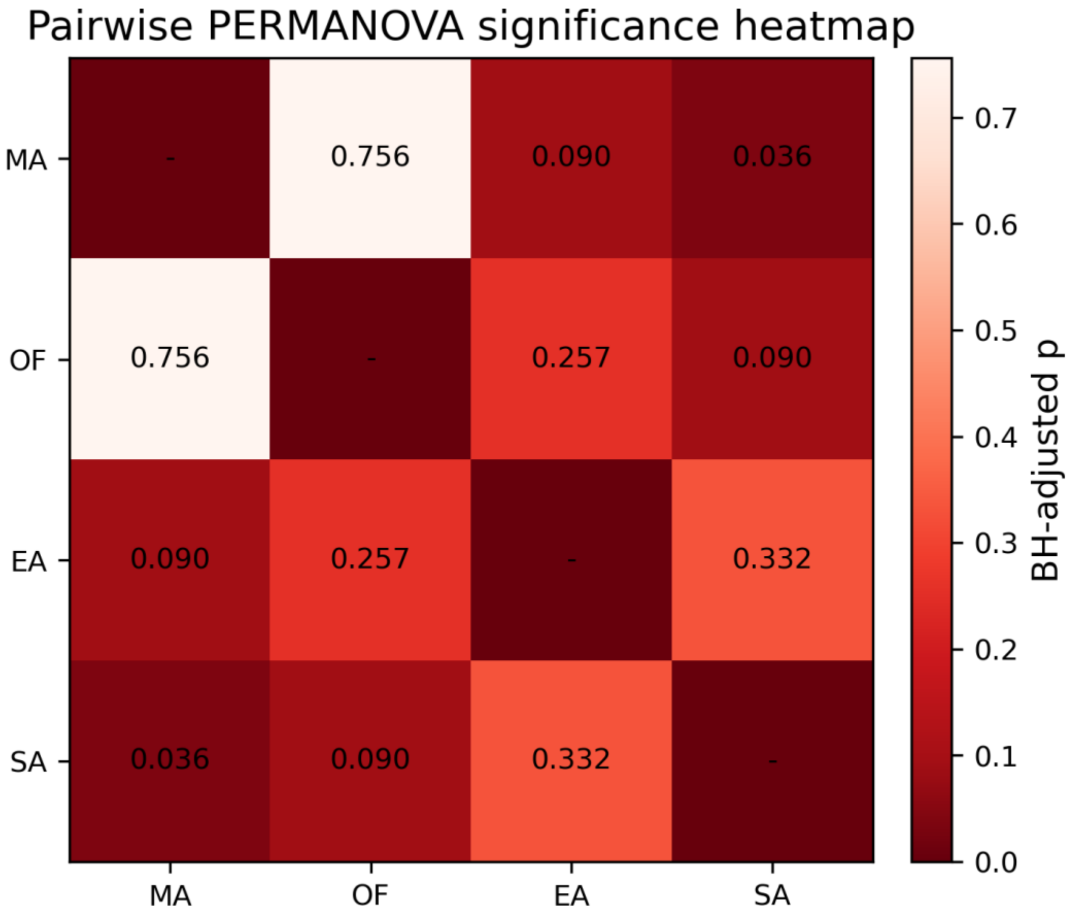

Figure S6. Pairwise PERMANOVA comparisons of macrobenthic community composition among the four habitat types. The heatmap shows Benjamini–Hochberg-adjusted p-values for pairwise PERMANOVA tests based on community dissimilarity. MA, muddy sediment area; OF, oyster farming area; EA, eastern rocky reef area; SA, southern rocky reef area.

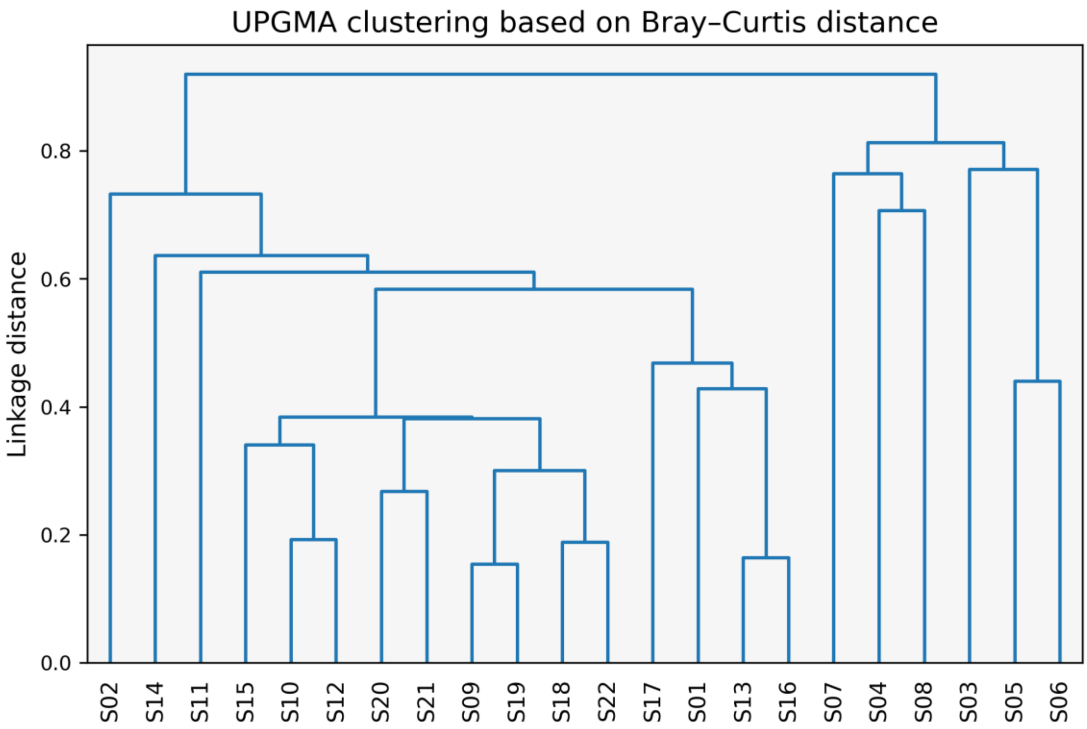

Figure S7. UPGMA clustering of sampling sites based on Bray–Curtis dissimilarity. The dendrogram illustrates similarities in macrobenthic community composition among the 22 sampling sites. Lower linkage distances indicate greater similarity between samples.

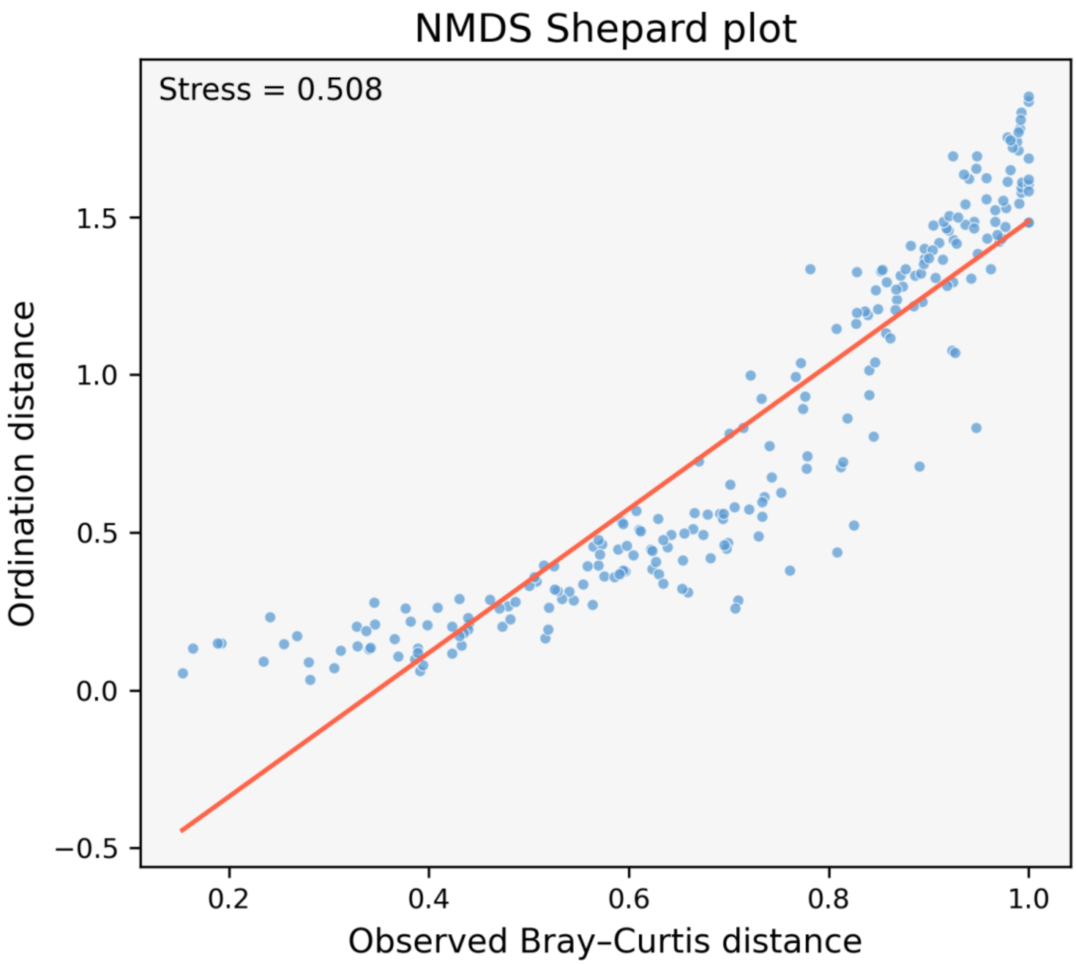

Figure S8. Shepard plot for the two-dimensional NMDS ordination based on Bray–Curtis dissimilarity. Points represent the relationship between observed Bray–Curtis dissimilarities and fitted ordination distances. The stress value indicates the goodness of fit of the NMDS configuration.

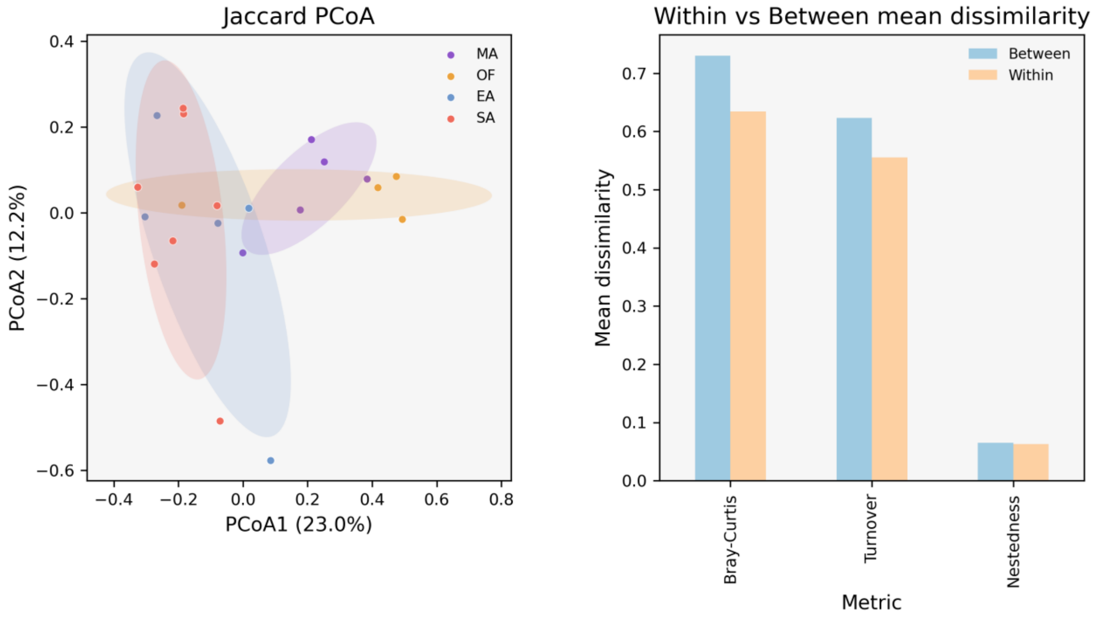

Figure S9. Beta-diversity patterns among the four habitat types. (a) Principal coordinate analysis based on Jaccard dissimilarity, with shaded ellipses representing the distribution of samples within each habitat type. (b) Mean within- and between-habitat Bray–Curtis dissimilarity, species turnover and nestedness components.

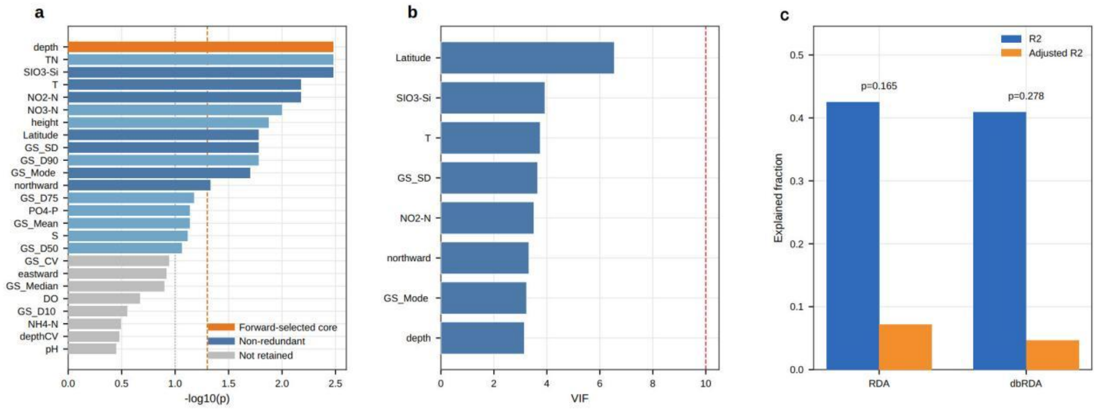

Figure S10. Environmental variable screening, collinearity filtering and constrained ordination model summary. (a) Univariate group-wise significance screening of environmental variables based on their associations with macrobenthic community beta-diversity structure. Orange bars indicate forward-selected core variables, blue bars indicate variables retained after collinearity filtering, and grey bars indicate variables not retained. Dashed vertical lines indicate screening thresholds. (b) Variance inflation factor (VIF) values of the final non-redundant environmental variable set, with the dashed red line indicating the VIF threshold of 10. (c) Summary of constrained ordination models showing the explanatory fractions of RDA and dbRDA, including unadjusted R^2 and adjusted R^2 values, with permutation-based p values shown above the bars.

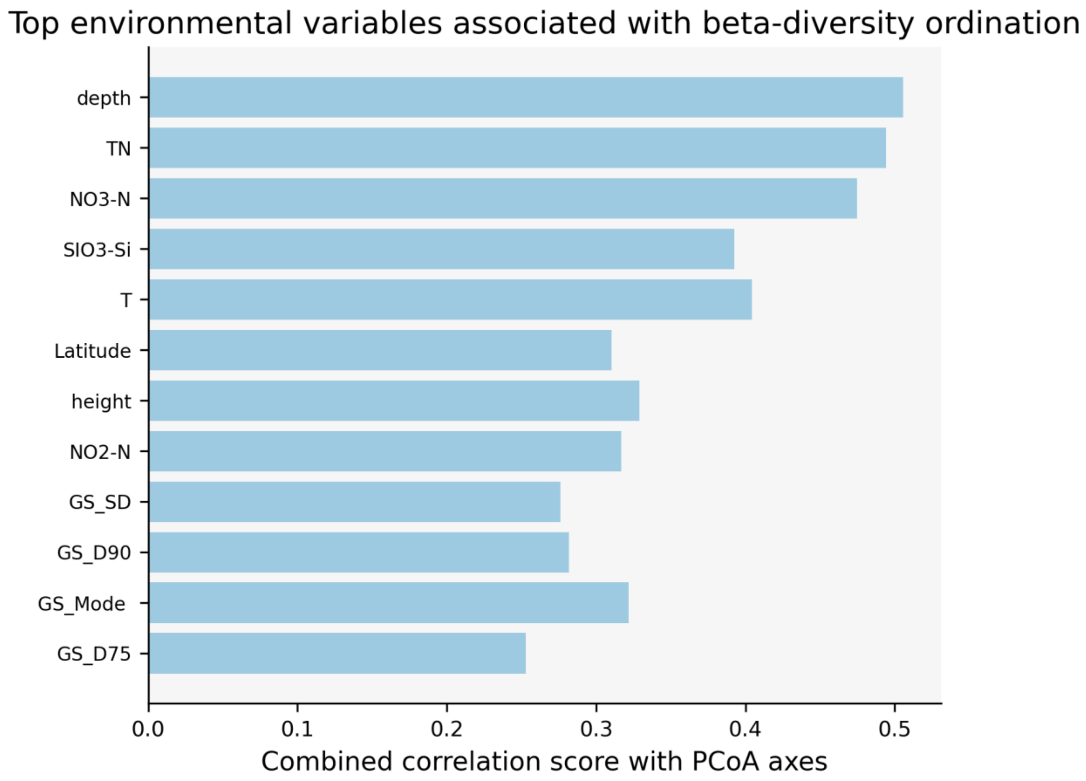

Figure S11. Environmental variables most strongly associated with macrobenthic beta-diversity ordination. Bars represent the combined correlation scores of environmental variables with the principal coordinate axes. Higher scores indicate stronger associations with variation in community composition.

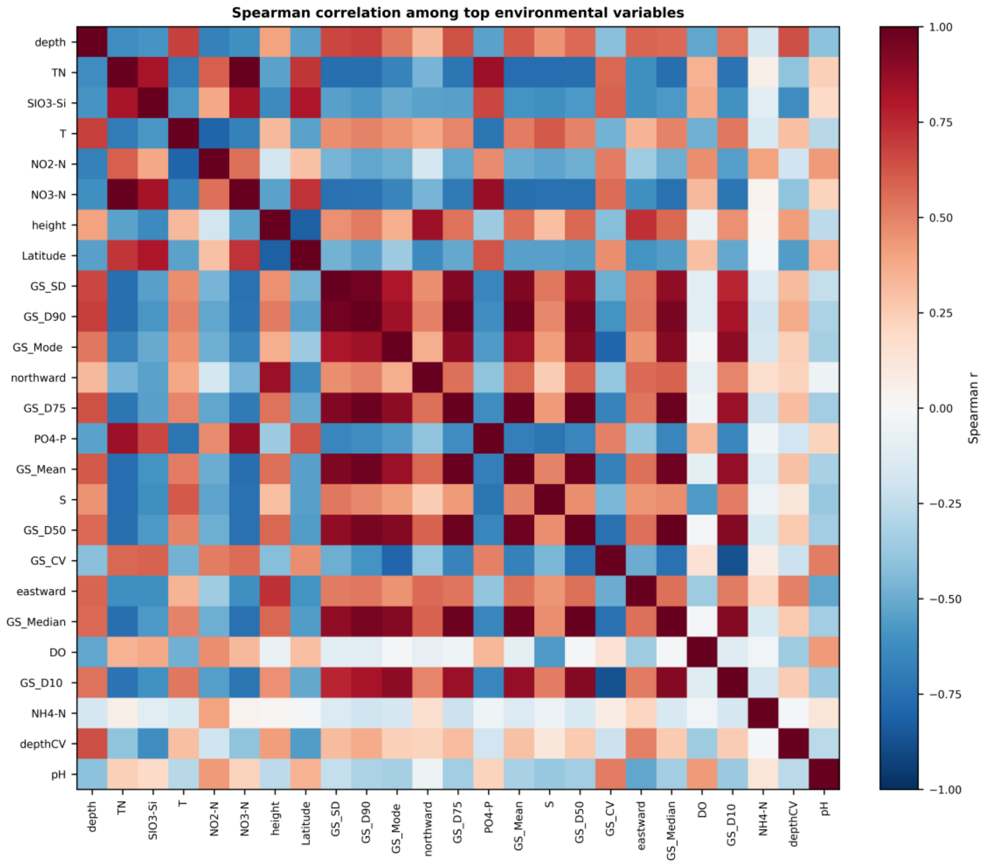

Figure S12. Spearman correlation matrix among candidate environmental variables. Colors indicate the direction and magnitude of Spearman's correlation coefficients, ranging from −1 to 1.

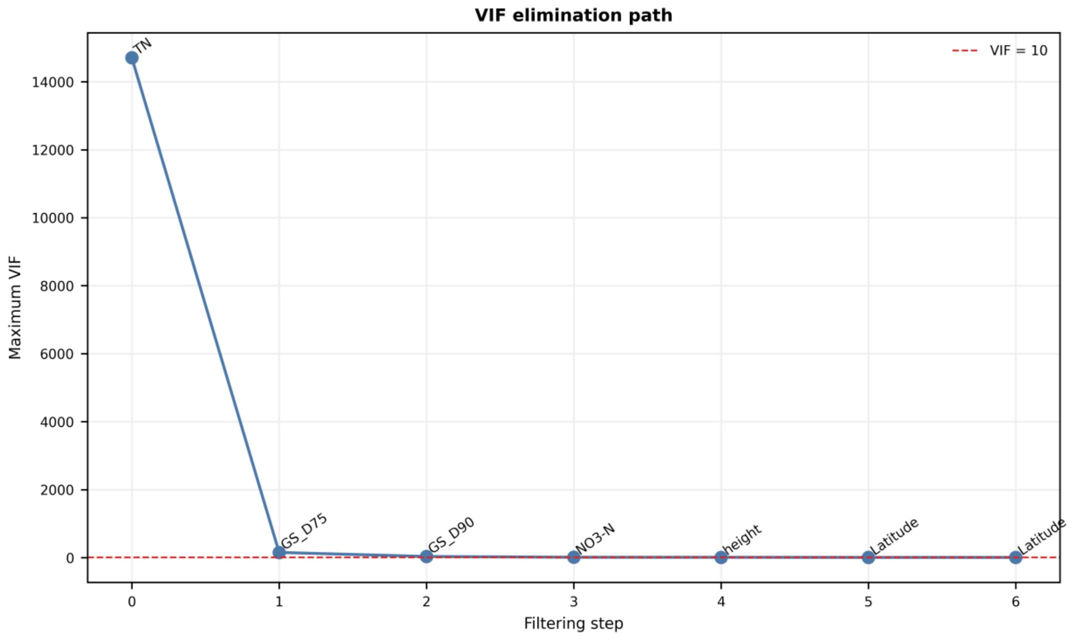

Figure S13. Sequential elimination of environmental variables based on variance inflation factors. The dashed line indicates the VIF threshold of 10, and labels show the variable removed at each filtering step.

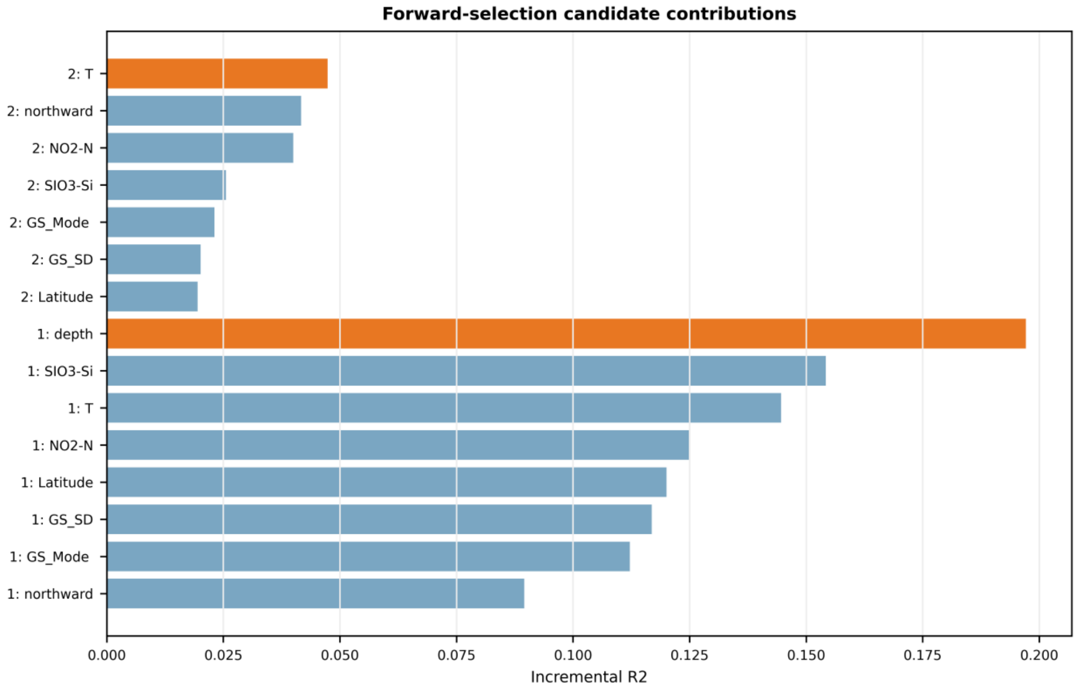

Figure S14. Incremental contributions of environmental variables during forward selection. Bars show the additional proportion of community variation explained by each candidate variable at successive selection steps; selected variables are highlighted.

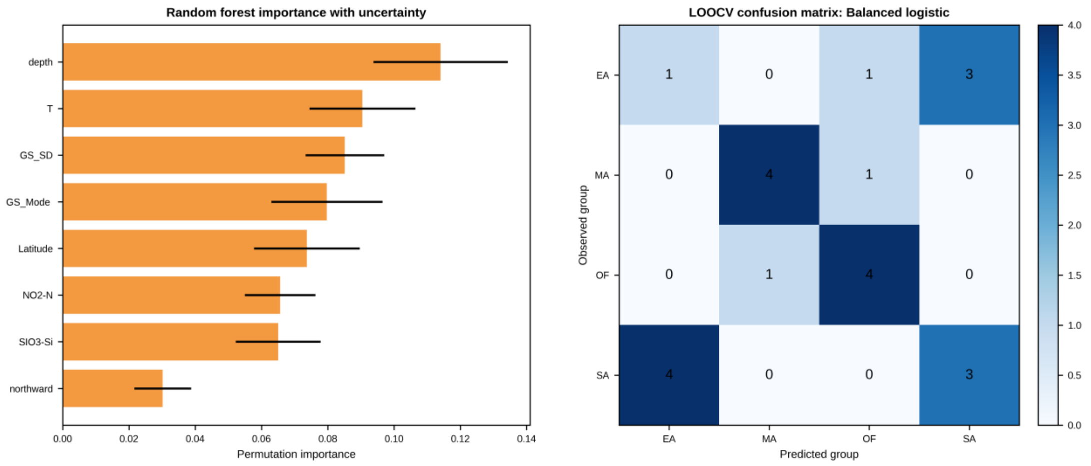

(a) **(b)**

Figure S15. Environmental variable importance and habitat-classification performance. (a) Random-forest permutation importance of retained environmental predictors, with error bars representing uncertainty. (b) Leave-one-out cross-validation confusion matrix for the balanced logistic regression model.

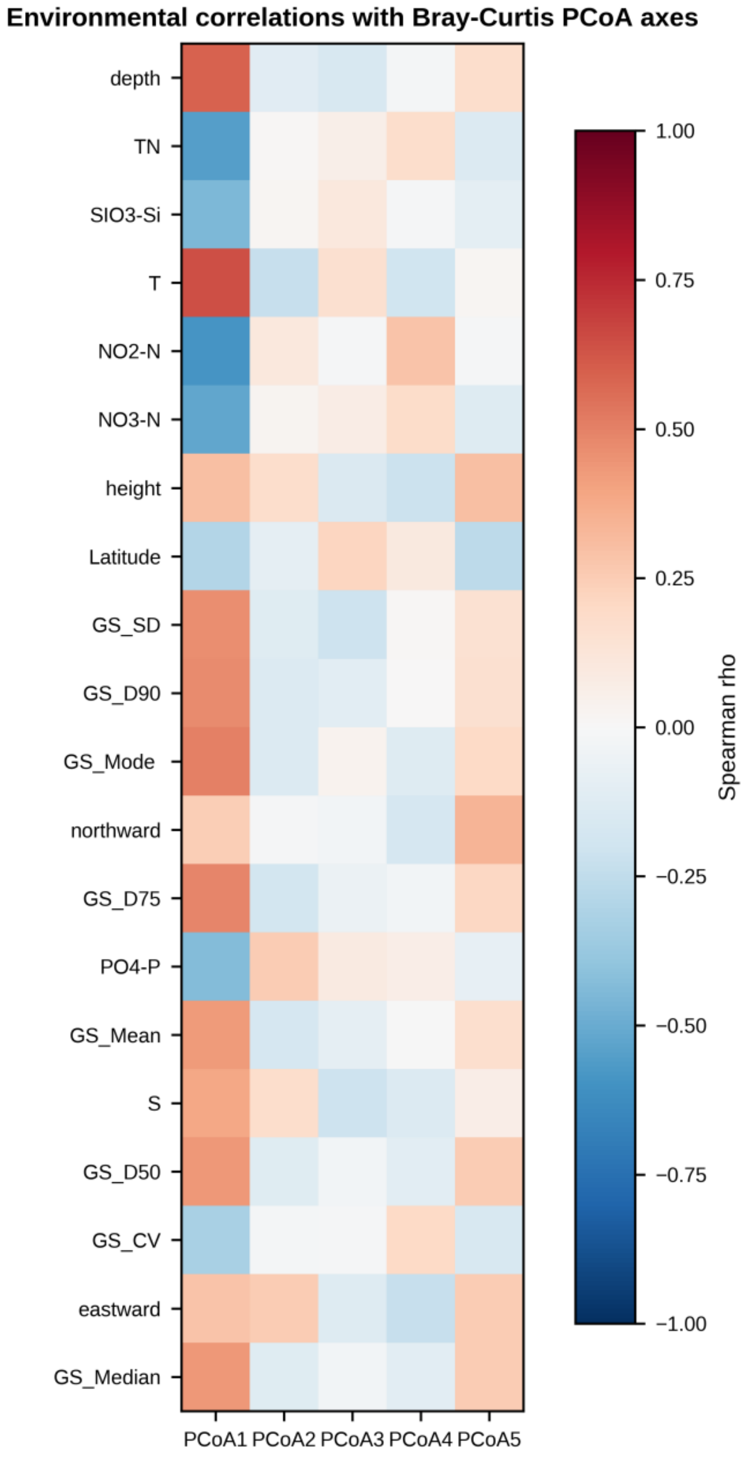

Figure S16. Correlations between environmental variables and Bray–Curtis PCoA axes. The heatmap shows Spearman's correlation coefficients between environmental variables and the first five PCoA axes; red and blue indicate positive and negative correlations, respectively.

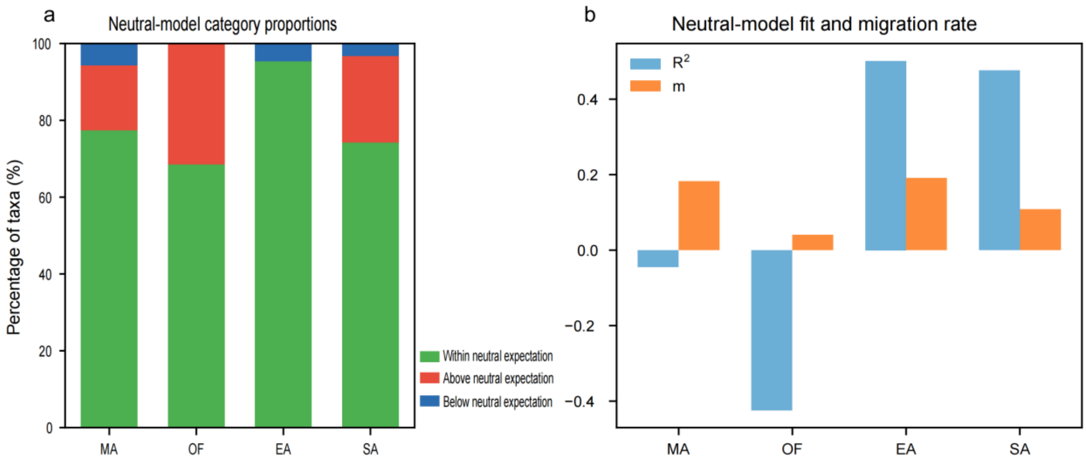

Figure S17. Neutral-model category proportions and group-level neutral-model statistics.

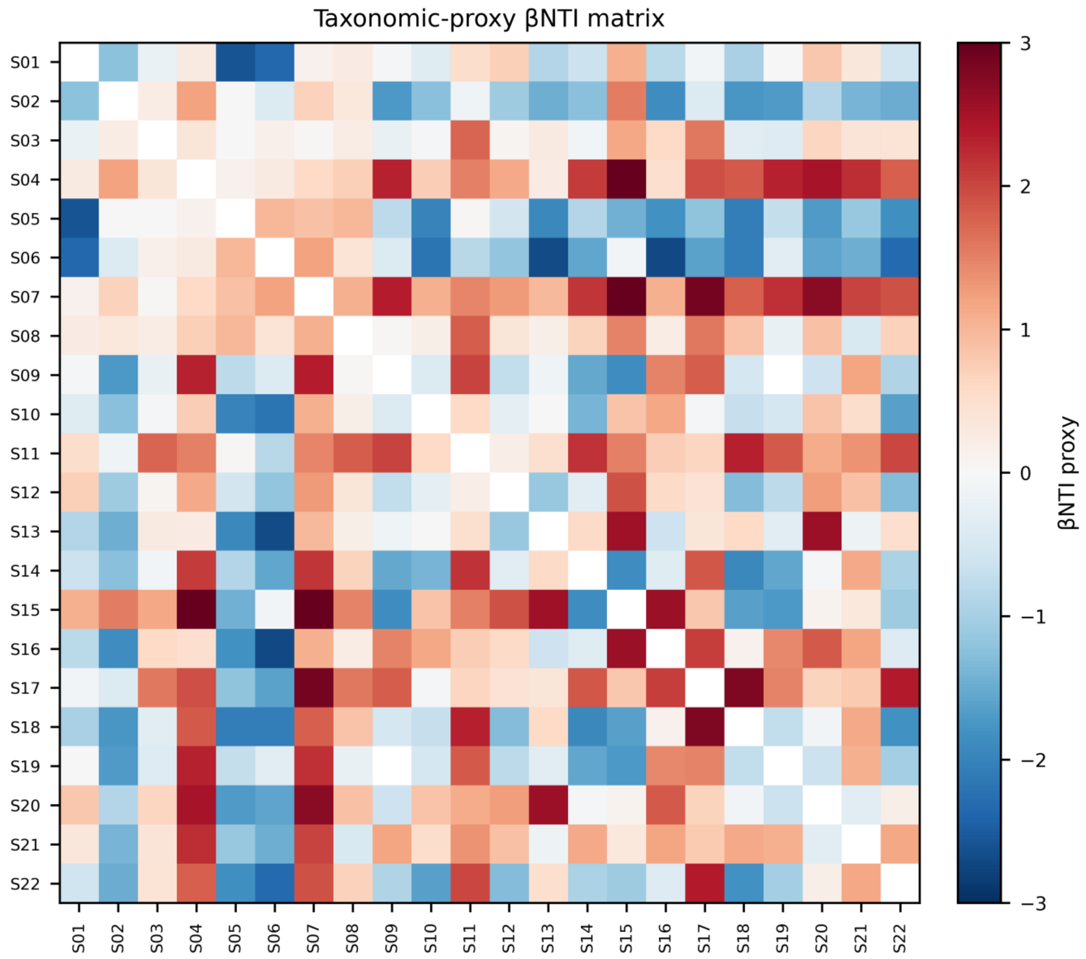

Figure S18. Taxonomic-proxy betaNTI heatmap among sites.

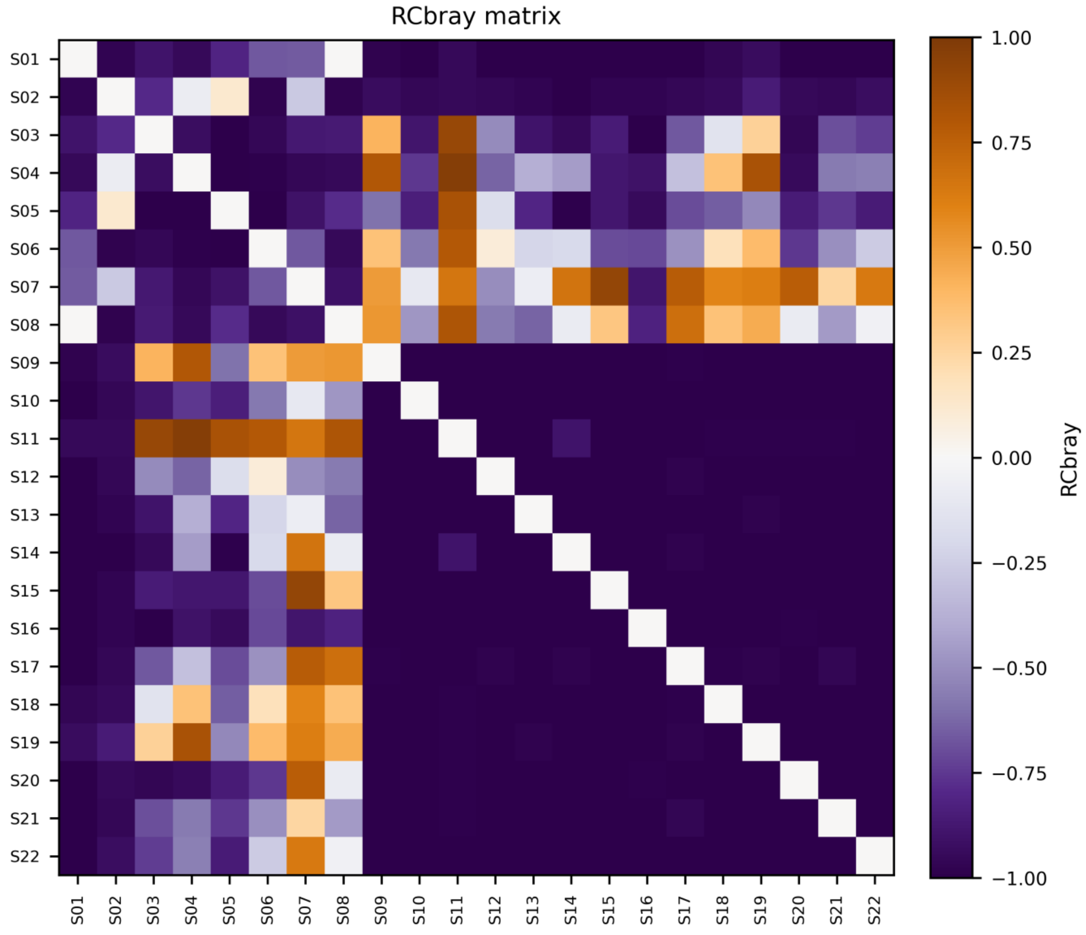

Figure S19. RCbray heatmap among sites.

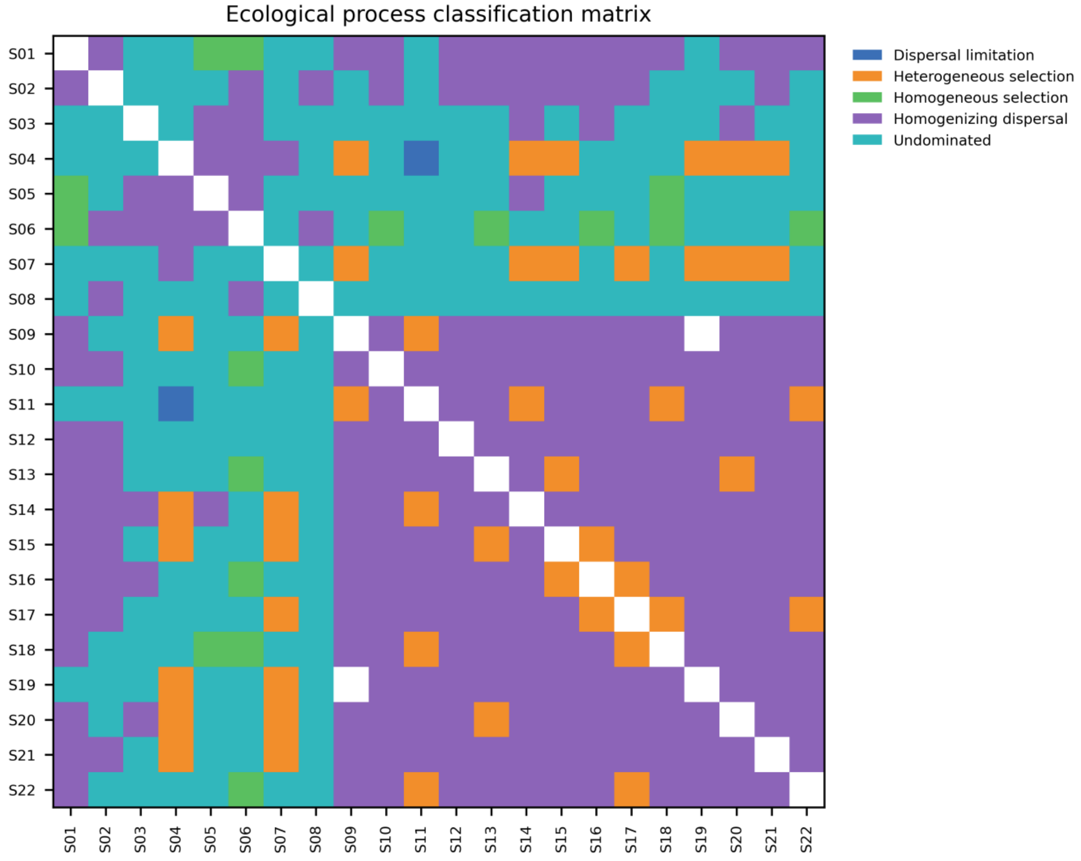

Figure S20. Ecological process classification matrix.

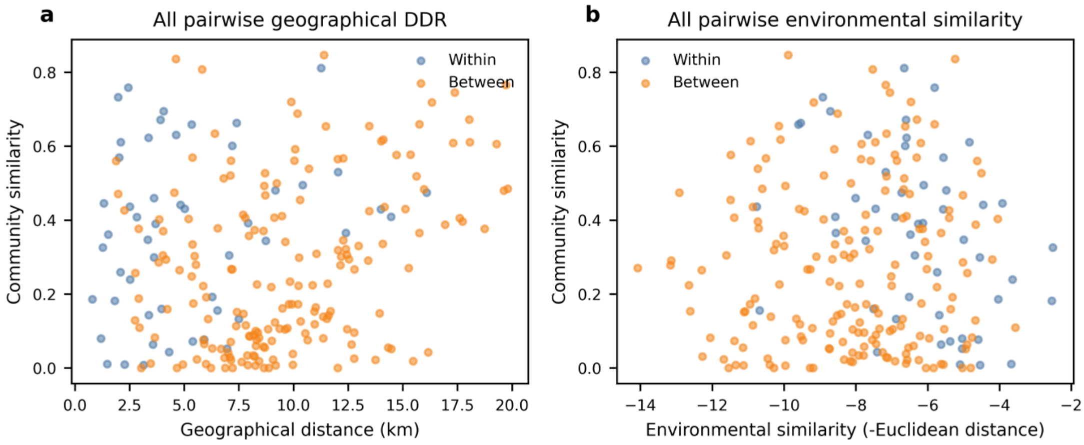

Figure S21. All-pair geographical distance-decay and environmental-similarity relationships.

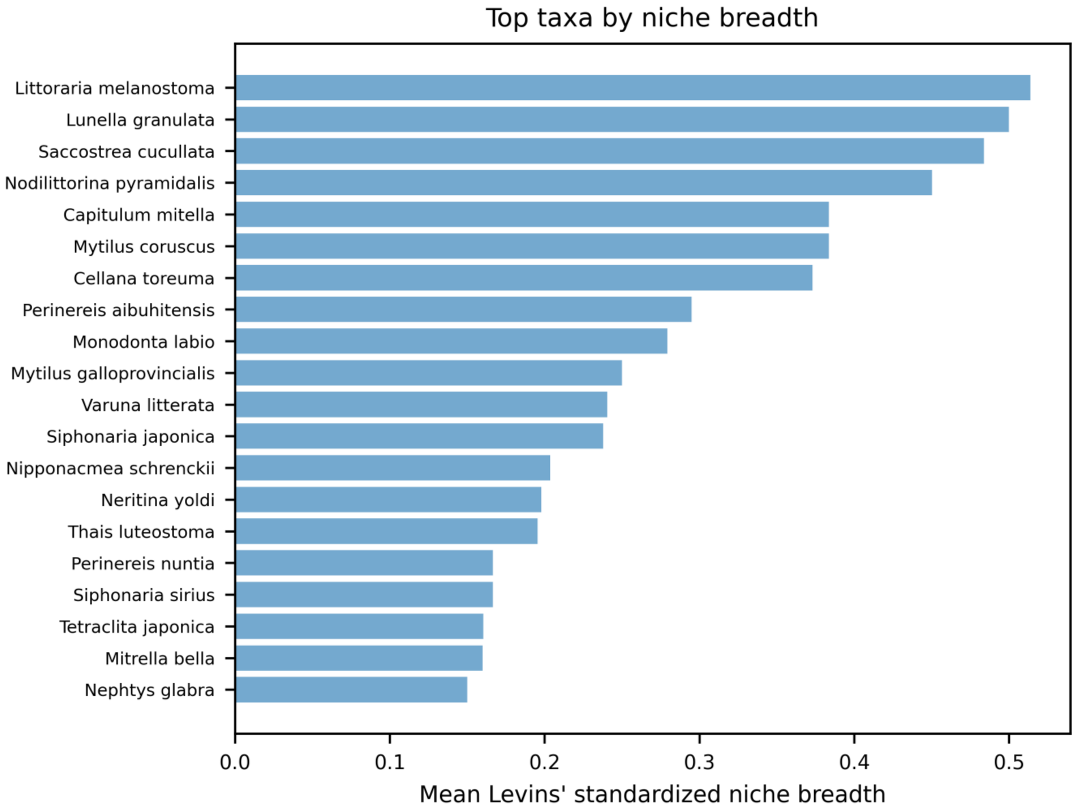

Figure S22. Top taxa ranked by mean Levins' standardized niche breadth.

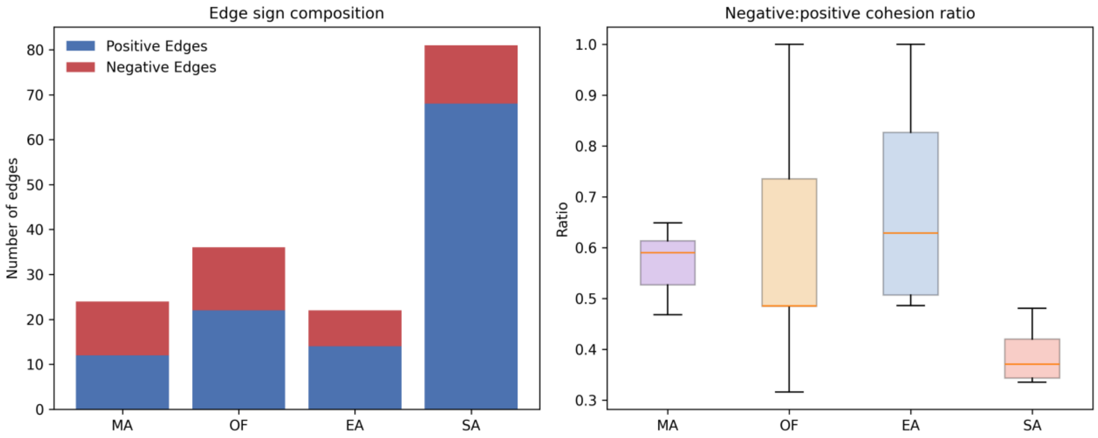

Figure S23. Edge-sign composition and cohesion balance of macrobenthic co-occurrence networks among habitat types. (a) Numbers of positive and negative associations in each network. (b) Distribution of the negative-to-positive cohesion ratio among habitat types.

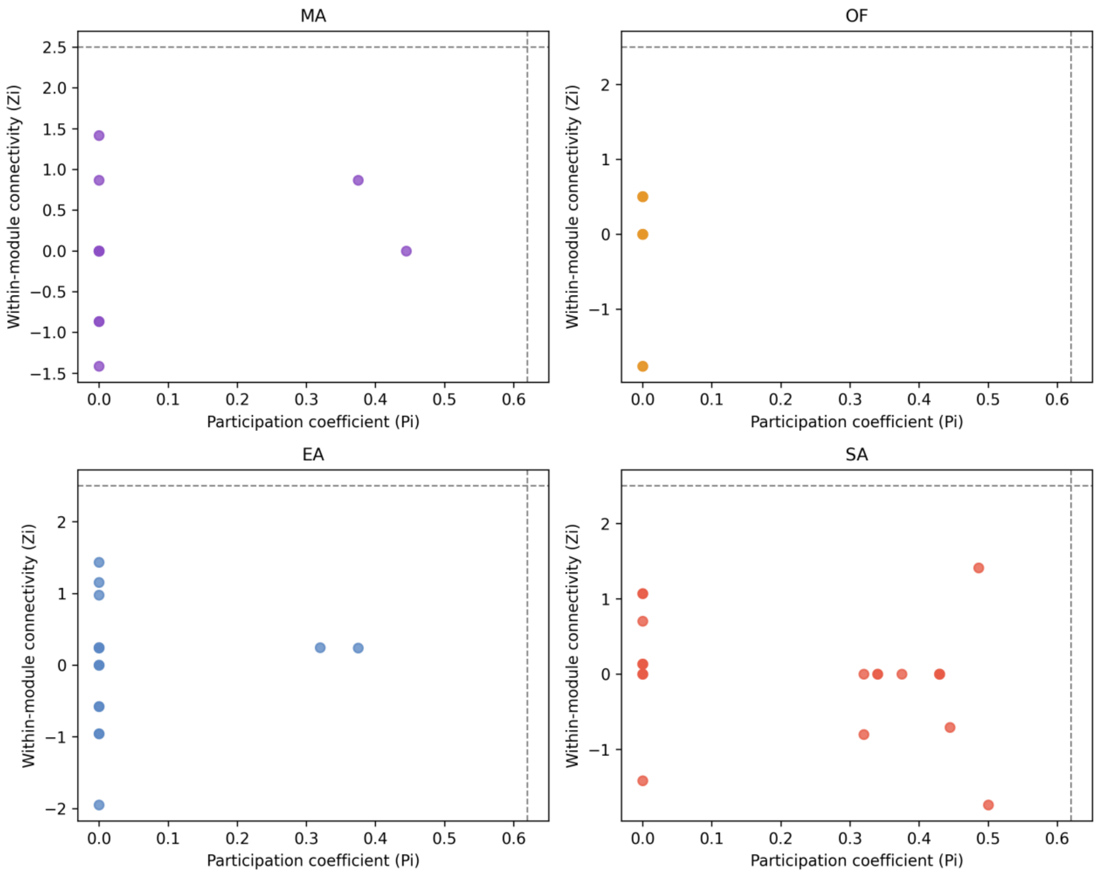

Figure S24. Node topological roles in macrobenthic co-occurrence networks among habitat types. Node roles were characterized using within-module connectivity (Zi) and participation coefficient (Pi). Dashed lines indicate the thresholds used to distinguish peripheral nodes, connectors, module hubs and network hubs.

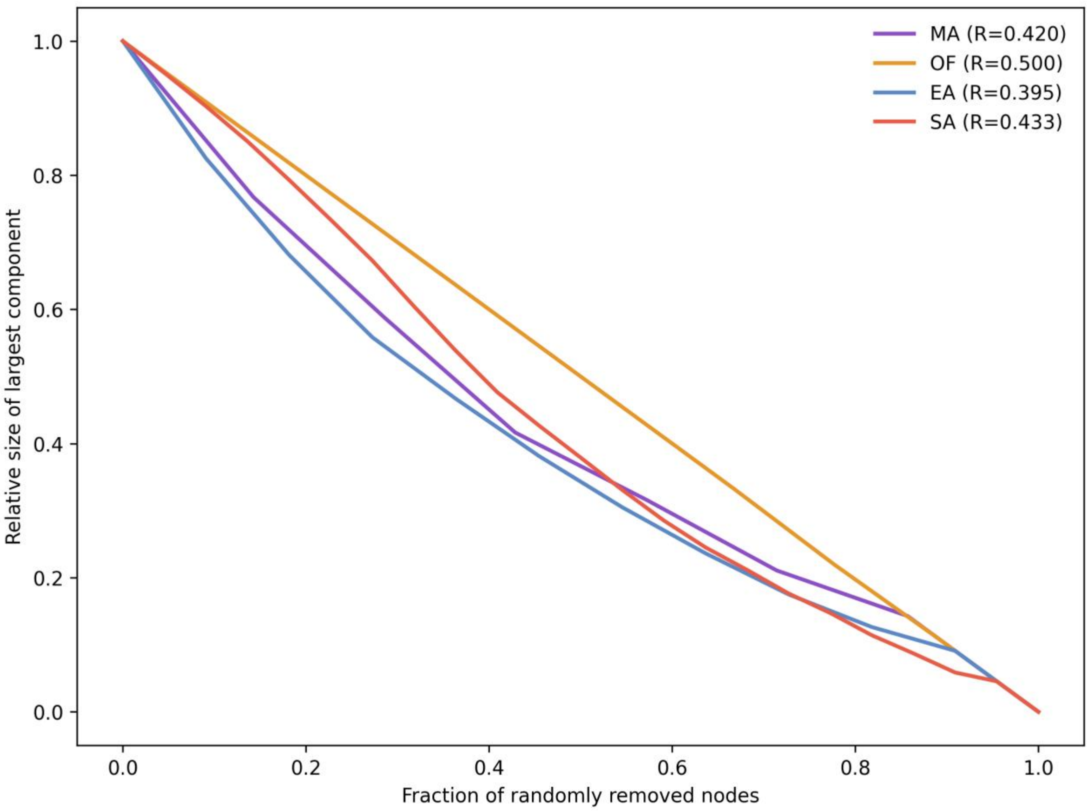

Figure S25. Robustness of macrobenthic co-occurrence networks under random node removal. Curves show the relative size of the largest connected component as increasing proportions of nodes were randomly removed. R represents network robustness.

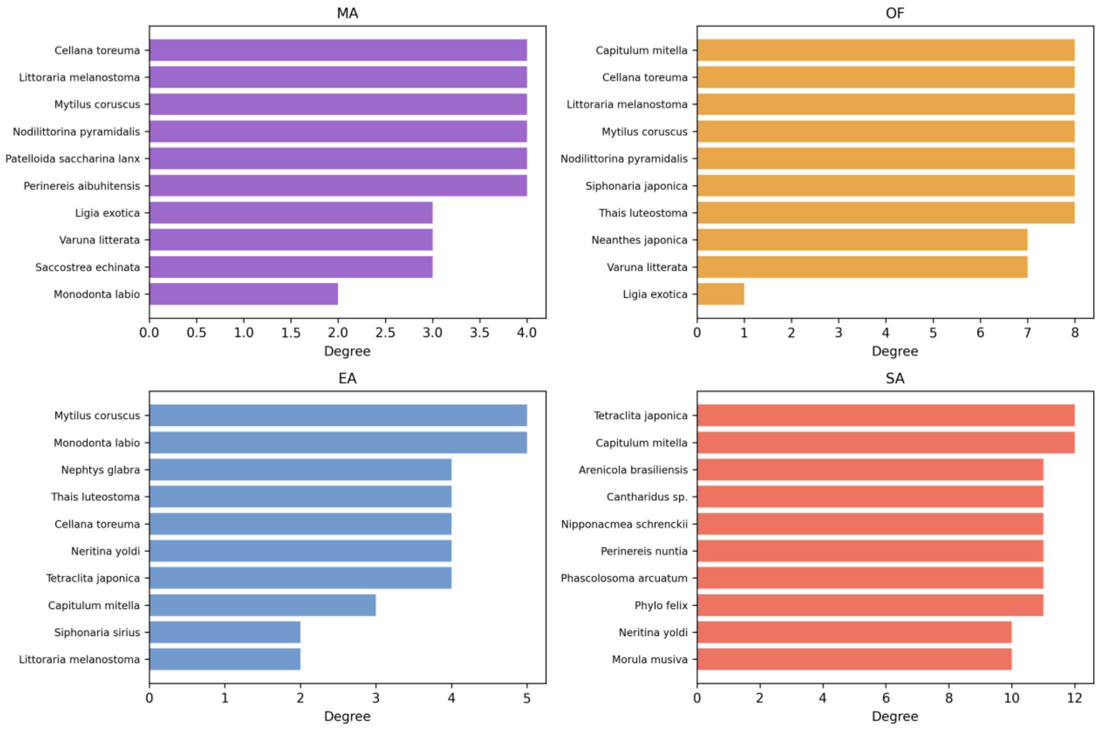

Figure S26. Taxa with the highest node degrees in the co-occurrence network of each habitat type. Bars represent the number of direct associations connected to each taxon.

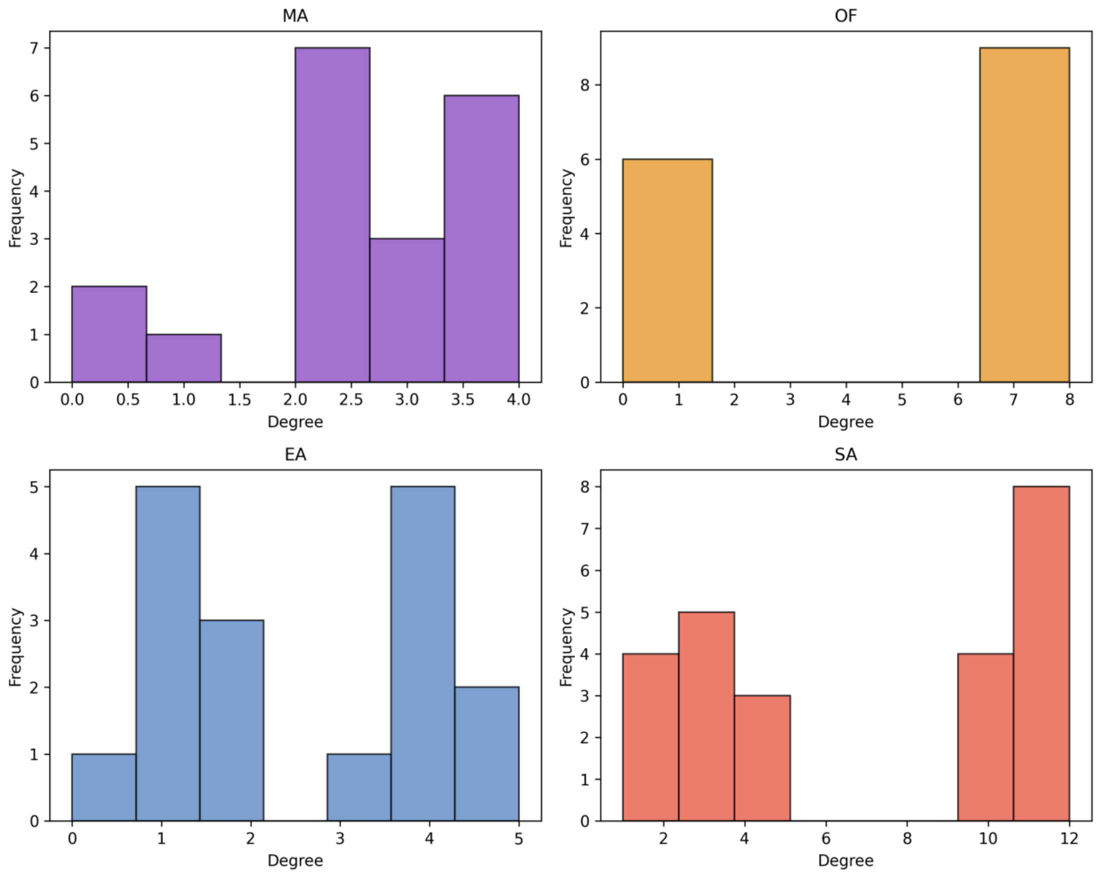

Figure S27. Degree-frequency distributions of macrobenthic co-occurrence networks among habitat types. Histograms show the frequency distribution of node degree within the MA, OF, EA and SA networks.

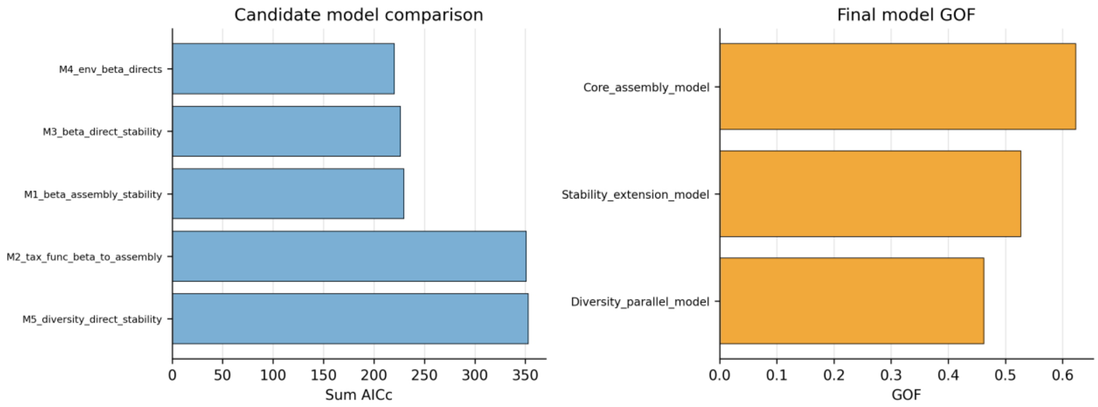

Figure S28. Comparison and goodness of fit of candidate PLS-SEM models. (a) Sum of corrected Akaike information criterion values for the candidate models. Lower AICc values indicate stronger relative model support. (b) Goodness-of-fit values for the core assembly, stability-extension and diversity-parallel models.

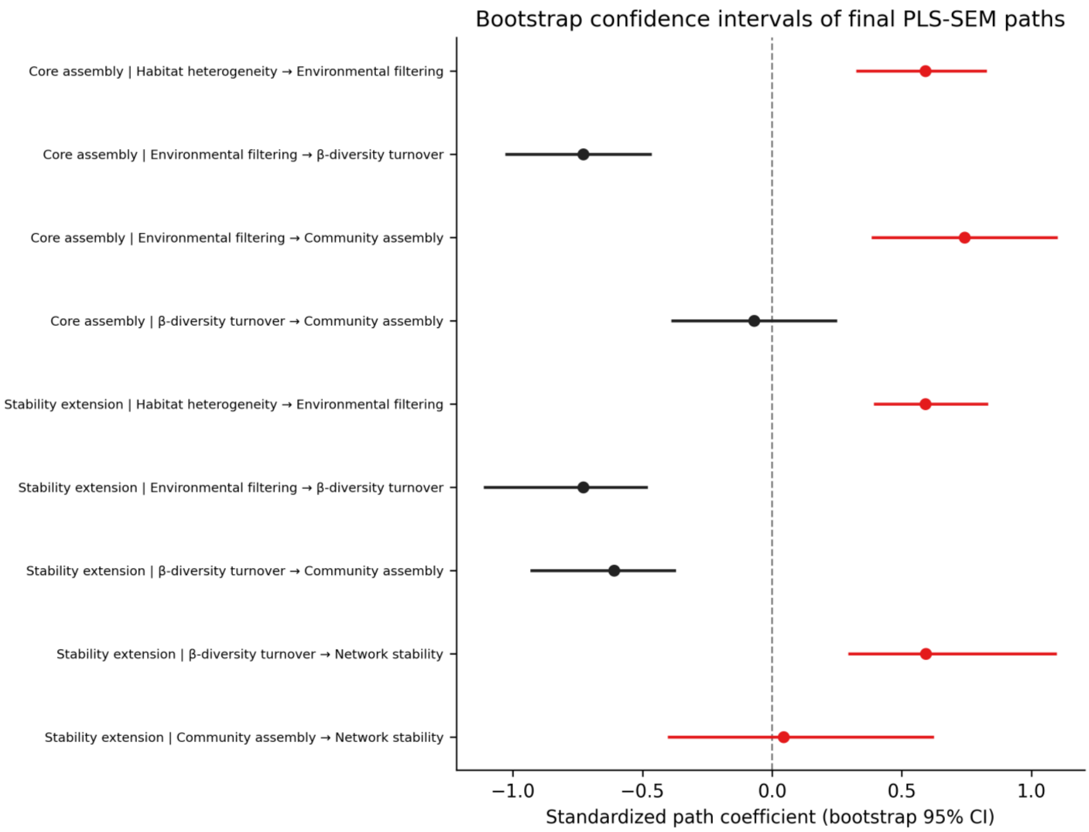

Figure S29. Bootstrap confidence intervals for standardized paths in the final PLS-SEM models. Points represent standardized path coefficients, and horizontal lines show bootstrap 95% confidence intervals. Intervals not overlapping zero indicate statistically supported pathways.

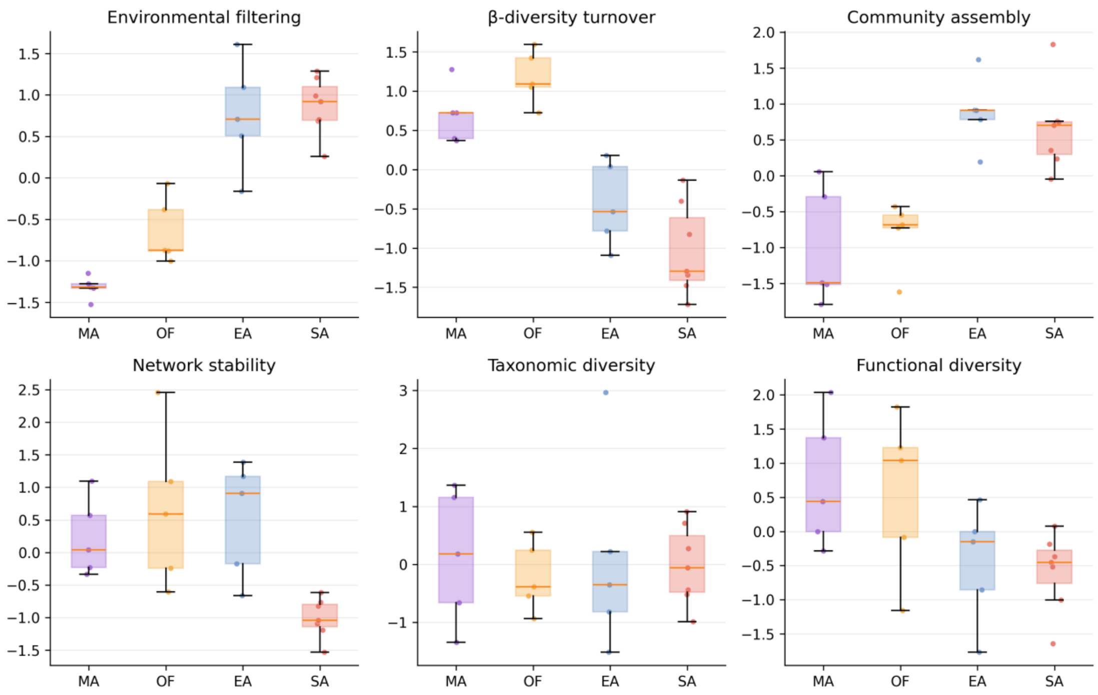

Figure S30. Distributions of standardized latent-variable scores among the four habitat types. Boxplots show environmental filtering, β-diversity turnover, community assembly, network stability, taxonomic diversity and functional diversity scores. Boxes represent interquartile ranges, horizontal lines indicate medians and points denote individual sites.

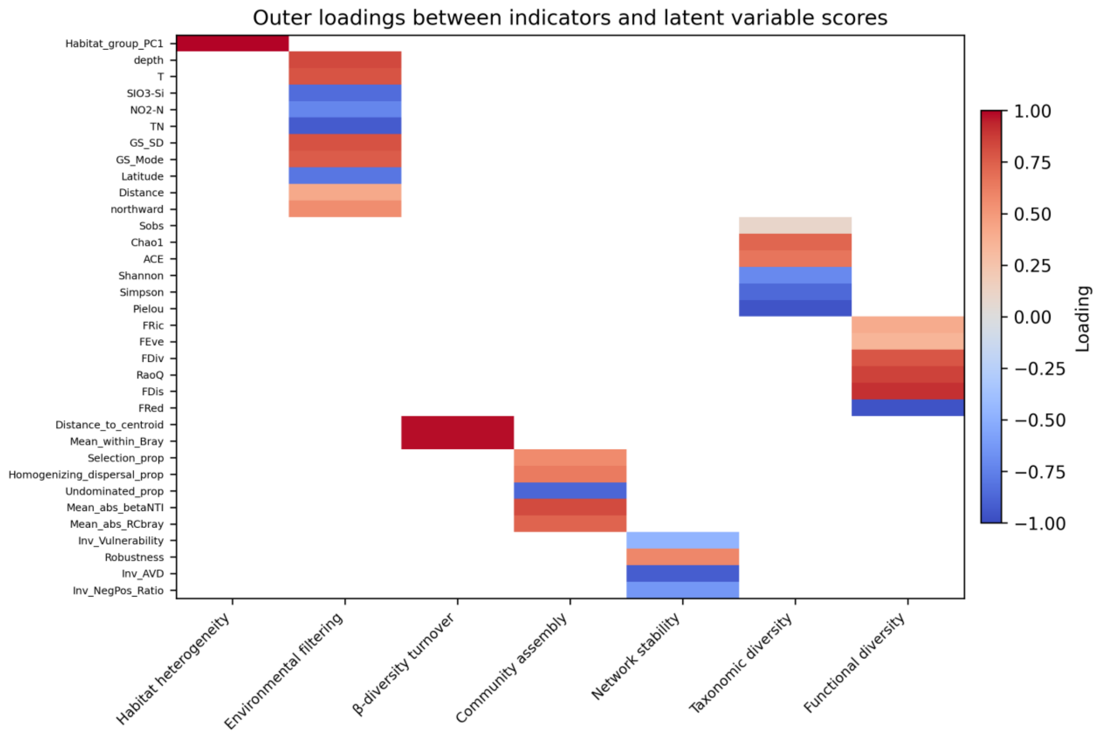

Figure S31. Outer loadings of observed indicators on latent constructs in the PLS-SEM measurement model. The heatmap shows the direction and magnitude of indicator loadings for habitat heterogeneity, environmental filtering, β-diversity turnover, community assembly, network stability, taxonomic diversity and functional diversity.

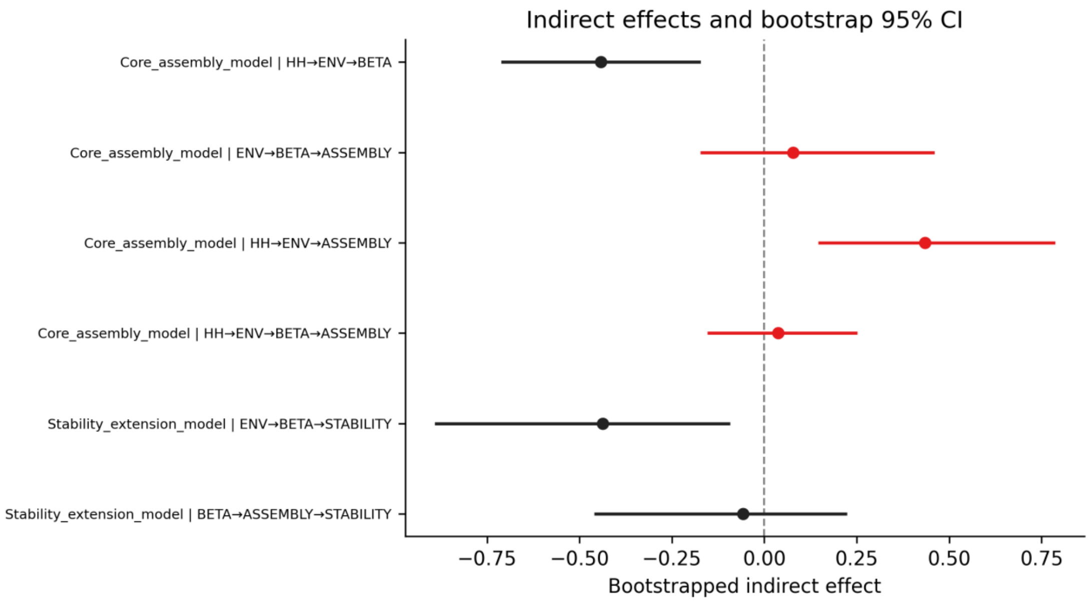

Figure S32. Bootstrapped indirect effects in the final PLS-SEM models. Points represent estimated indirect effects, and horizontal lines indicate bootstrap 95% confidence intervals. Confidence intervals excluding zero identify statistically supported indirect pathways.
